# The metabolic potential of *Escherichia coli* BL21 in defined and rich medium

**DOI:** 10.1186/1475-2859-13-45

**Published:** 2014-03-23

**Authors:** Zhaopeng Li, Manfred Nimtz, Ursula Rinas

**Affiliations:** 1 Helmholtz Centre for Infection Research, Inhoffenstraße 7, D-38124 Braunschweig, Germany; 2 Leibniz University of Hannover, Technical Chemistry–Life Science, Callinstraße 5, D-30167 Hannover, Germany

**Keywords:** *Escherichia coli*, Growth rate control, Metabolic balance, Overflow metabolism, Proteome, Stationary phase response, Transcriptional control, Two-dimensional gel electrophoresis

## Abstract

**Background:**

The proteome reflects the available cellular machinery to deal with nutrients and environmental challenges. The most common *E. coli* strain BL21 growing in different, commonly employed media was evaluated using a detailed quantitative proteome analysis.

**Results:**

The presence of preformed biomass precursor molecules in rich media such as Luria Bertani supported rapid growth concomitant to acetate formation and apparently unbalanced abundances of central metabolic pathway enzymes, e.g. high levels of lower glycolytic pathway enzymes as well as pyruvate dehydrogenase, and low levels of TCA cycle and high levels of the acetate forming enzymes Pta and AckA. The proteome of cells growing exponentially in glucose-supplemented mineral salt medium was dominated by enzymes of amino acid synthesis pathways, contained more balanced abundances of central metabolic pathway enzymes, and a lower portion of ribosomal and other translational proteins. Entry into stationary phase led to a reconstruction of the bacterial proteome by increasing e.g. the portion of proteins required for scavenging rare nutrients and general cell protection. This proteomic reconstruction during entry into stationary phase was more noticeable in cells growing in rich medium as they have a greater reservoir of recyclable proteins from the translational machinery.

**Conclusions:**

The proteomic comparison of cells growing exponentially in different media reflected the antagonistic and competitive regulation of central metabolic pathways through the global transcriptional regulators Cra, Crp, and ArcA. For example, the proteome of cells growing exponentially in rich medium was consistent with a dominating role of phosphorylated ArcA most likely a result from limitations in reoxidizing reduced quinones in the respiratory chain under these growth conditions. The proteomic alterations of exponentially growing cells into stationary phase cells were consistent with stringent-like and stationary phase responses and a dominating control through DksA-ppGpp and RpoS.

## Background

*Escherichia coli* is still the most utilized bacterial workhorse for the production of biomolecules, in particular the strain BL21 is the most employed host for the production of recombinant proteins. *E. coli* BL21 is considered a very robust strain compared to *E. coli* K12 strains as it produces less acetate [1,2]. Acetate, produced as major by-product during fast growth in carbon excess conditions, negatively effects the production of proteins and other biomolecules.

For rapid and convenient lab-scale production of recombinant proteins, cells are usually grown in rich medium such as Luria Bertani or Terrific broth. However, for large-scale recombinant protein production, defined mineral salt media are generally preferred as they allow the implementation of fed-batch based high-cell density cultivations [3]. *E. coli* is not only used as host for recombinant protein production but is also gaining increasing importance in synthetic biology for the production of heterologous low molecular weight compounds [4-6] and also for overproduction of homologous metabolites such as amino acids [7] or for the utilization as a whole cell biocatalyst employed in biotransformation processes [8]. Thus, a more profound comprehension of the metabolic potential of exponentially growing or stationary phase and resting cells is of considerable importance to understand and improve production processes but also for the pure knowledge gain to better understand bacterial physiology.

Based on a proteomic study we present here for the first time a detailed description of the metabolic potential of *E. coli* BL21 during growth in defined and rich medium. The quantitative proteome analysis was not only done for exponentially growing cells but also for stationary phase cells to analyze the metabolic capabilities as well as the adaptation potential to changing environmental conditions.

## Results

**Figure 1:**
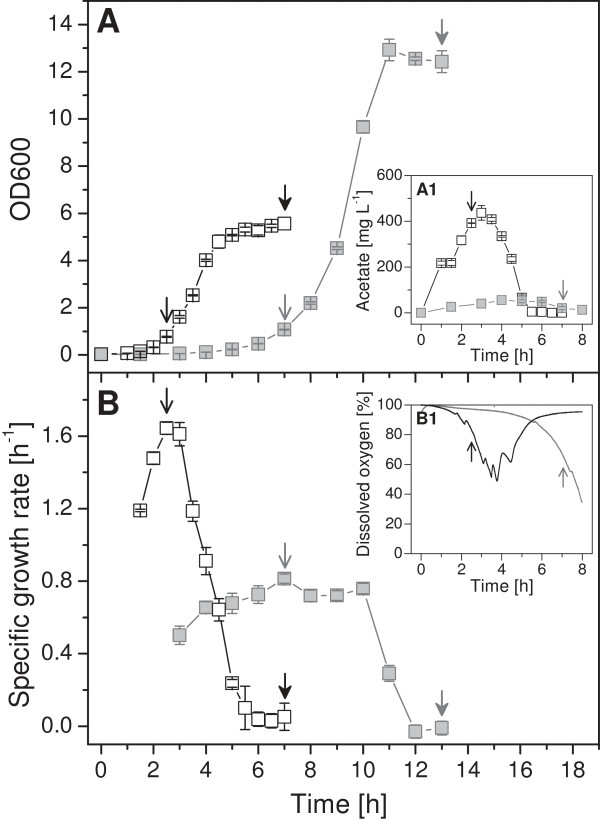
**Growth properties of *E. coli *BL21 in defined and rich medium.** Cells were grown in glucose supplemented mineral salt medium (DNB, gray symbols, gray solid line and arrows) and rich medium (LB, open symbols, black solid line and arrows) at 37°C. Samples for proteome analysis were taken at the time-points indicated: exponential phase (open arrow) and stationary phase (stealth arrow). Cell growth (OD600), specific growth rate, acetate and dissolved oxygen concentrations are given.

**Figure 2:**
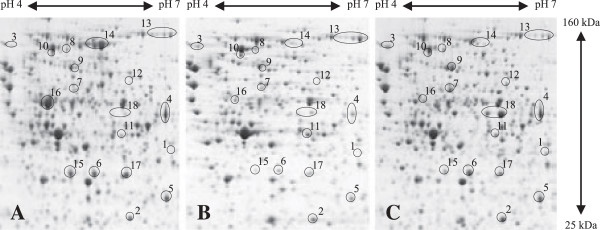
**Proteomic fingerprint of cells growing in defined and rich medium.** Cells growing at exponential phase in glucose supplemented mineral salt medium **(A)** and in LB medium at exponential **(B)** and stationary phases **(C)** were analyzed by 2D gel electrophoresis. Images of gel sections indicating representative metabolic enzymes are shown: upper glycolysis, FbaB 1; lower glycolysis, GpmA 2, PpsA 3; TCA cycle: GltA 4, SucD 5, Mdh 6; anaplerotic reactions, PckA 7, MaeB 8; by-product metabolism, Acs 9, Pta 10, AckA 11, PoxB 12, AdhE 13; amino acid biosynthesis, MetE 14, IlvE 15, IlvC 16, CysK 17, and amino acid degradation, TnaA 18. Sampling time-points are indicated in Figure 1. The entire gel images representing the proteome of cells grown in defined and rich (LB and TB) media at exponential and stationary phases indicating all identified proteins are given in the Additional file 1: Figures S1-S6.

**Figure 3:**
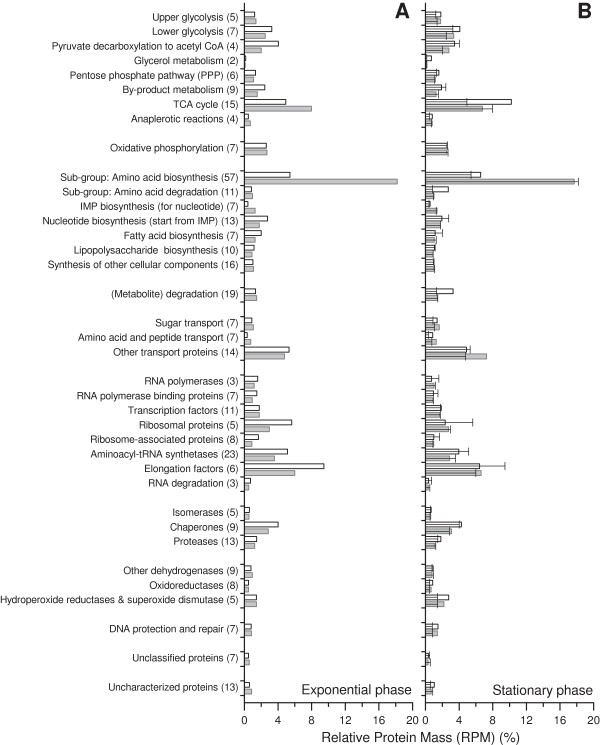
**Functional quantitative proteomic overview of cells growing in defined and rich medium.** Summary of proteome data of *E. coli* BL21 grown at exponential **(A)** and stationary phases (**B**, abundances at exponential phase are indicated in **B** also by solid lines for comparison) in defined (gray bars) and rich (open bars) media. Numbers in brackets after each functional category are the number of proteins that were identified within this category. An average of protein abundances from cells grown in LB and TB media was calculated to represent the proteome of cells grown in rich medium. The entire data sets are given in Additional file 1: Table S1.

During growth in rich medium such as the commonly employed media Luria Bertani or Terrific Broth cells do not need to synthesize most of the precursor molecules (e.g. amino acids) as they are already present in the medium. Thus, cells can spend more resources to produce the macromolecules required for their own proliferation and can grow more rapidly compared to cells growing in defined mineral salt medium (Figure 1). Remarkably, *E. coli* BL21 also secretes low but detectable levels of acetate during rapid growth in rich medium even in the absence of oxygen limiting conditions (Figure 1). For a better understanding of the bacterial physiology of *E. coli* BL21 under these different conditions of nutrient availability a detailed comparative analysis of the bacterial proteome was carried out. A first brief visual examination revealed a less complex nature of the proteome during rapid growth in rich medium compared to the slower growth in the glucose supplemented mineral salt medium (Figure 2). Most notably, enzymes required for amino acid biosynthesis are virtually absent during rapid growth in rich medium (Figure 2). A lumped quantitative analysis of the abundance levels of all identified proteins in cells from exponential and stationary phase in defined and rich media is given in Figure 3 and in more detail visualized in Additional file 1: Figures S1-S9 with corresponding values shown in the Additional file 1: Table S1 (values with standard deviation also in Additional file 2: Table S5).

### Central (carbon) metabolism

**Figure 4:**
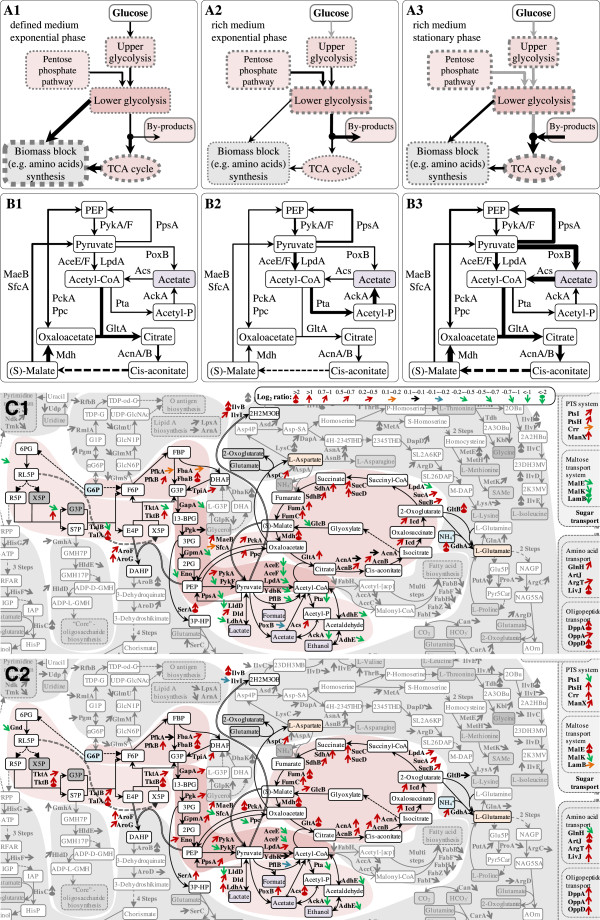
**Central metabolic pathway proteome at different growth conditions.** Comparative abundances of (lumped) central metabolic pathway enzymes of *E. coli* BL21 grown in defined medium at exponential phase **(A1 and B1)** and in rich medium at exponential **(A2 and B2)** and stationary phases **(A3 and B3)**. The thickness of the arrows and dotted borders represents the relative (lumped) abundances of enzymes belonging to the pathways at these growth conditions. A detailed direct comparison of pathway regulation at the proteomic level at different growth conditions (**C1**, defined versus rich media at exponential phase and **C2**, stationary versus exponential phase in rich medium) is given in a pathway map using different color coded arrows for the representation of the comparative Log_2_ ratios of the single enzyme abundances [code given in **C1**, **C1**: Log_2_(defined/rich), **C2**: Log_2_(stationary/exponential)]. An average of single enzyme abundances from cells grown in LB and TB media was calculated to represent the single enzyme abundance in cells grown in rich medium. The entire comparative proteomic (pathway) data are visualized in Additional file 1: Figures S7-S9 and the corresponding values are given in the Additional file 1: Table S1. The list of abbreviations is found at the end of the Additional file 1: Figure S9.

The comparative proteome analysis of cells growing exponentially in defined and rich medium with special attention to enzymes of the central metabolic pathways revealed a complex picture corroborating a “suboptimal” coupling of catabolic and anabolic pathways especially during rapid growth in rich medium also for *E. coli* BL21 (Figure 4).

#### Glycolysis, pyruvate dehydrogenase, and pentose phosphate pathway

Enzymes of the upper glycolytic pathway are present in slightly higher amounts in cells growing in defined medium with glucose as carbon substrate compared to the cells growing in rich medium (Figures 3 and 4). However, the opposite is observed for the lower glycolytic pathway; enzymes belonging to this part of the glycolysis and also the subunits of the pyruvate dehydrogenase complex, the enzyme connecting the glycolytic pathway and the tricarbocyclic acid cycle, are present in significantly lower amounts in cells growing in defined medium (Figures 3 and 4). Also, most identified enzymes belonging to the pentose phosphate pathway (e.g. Gnd, TktA, TalB, and Eda) are found in lower amounts in cells growing in defined medium (Figures 3 and 4). Thus, the higher amounts of enzymes of the lower glycolytic and pentose phosphate pathways as well as of pyruvate dehydrogenase during rapid growth in rich medium point to an increased capacity for carbon flux leading towards connecting pathways (e.g. tricarbocyclic acid cycle) under these growth conditions.

#### Tricarbocyclic acid (TCA) cycle and metabolism of acetate and fermentative by-products

In contrast to the enzymes of the lower glycolytic and the pentose phosphate pathways, the enzymes of the TCA cycle accumulate to higher levels in cells growing in defined medium, respectively, to lower levels in cells growing in rich medium (Figures 3 and 4). Thus, larger amounts of enzymes of the lower glycolytic and pentose phosphate pathways as well as of pyruvate dehydrogenase and lower amounts of TCA cycle enzymes during rapid growth in rich medium point here to a potential bottleneck leading to off-pathway carbon flux at this junction and to acetate formation. In this line, enzymes catalyzing the formation of acetate from acetyl-CoA (Pta and AckA) are present at elevated level in cells growing exponentially in rich medium (Figure 4). The enzymes responsible for acetate formation directly from pyruvate (PoxB) and acetate uptake through acetyl-CoA (Acs) are neither in defined nor in rich medium present in large amounts (Figure 4, Additional file 1: Table S1). Thus, acetate uptake through Acs but also acetate formation through PoxB does not seem to play a significant role during exponential growth in both media. Interestingly, cells growing exponentially in rich medium also exhibit higher levels of AdhE (Figure 4, Additional file 1: Table S1), the enzyme catalyzing the anaerobic formation of ethanol from acetyl-CoA [9-11], despite the absence of cell external anaerobic or even oxygen limiting conditions (Figure 1) confirming the bottleneck at the junction of the pyruvate dehydrogenase and the TCA cycle, or more general, at the pyruvate node.

#### Anaplerotic reactions

Enzymes catalyzing anaplerotic reactions between glycolysis and TCA cycle intermediates in both directions (e.g. Ppc, PckA, MaeB) accumulate to slightly higher amounts in cells growing exponentially in defined medium (Figures 3 and 4) also pointing to a more balanced (flux) connection between both pathways under these growth conditions. On the contrary, lower amounts of anaplerotic and TCA cycle enzymes and higher amounts of lower glycolytic and pentose phosphate pathway enzymes as well as higher amounts of the pyruvate dehydrogenase are in line with the elevated flux towards acetate observed during rapid growth in rich medium.

#### Respiratory energy generation

The majority of NADH is generated in the TCA cycle and reoxidized through electron transfer to oxidized quinones catalyzed by NADH dehydrogenases. Concurrently exported protons are reimported driving energy generation through ATPases. Identified proteins belonging to both types of enzymes (e.g. NuoB/C/G/F and AtpA/D/H) do not constitute a large part of the bacterial proteome and do not show significant changes in response to nutrient availability (Figure 3, Additional file 1: Table S1) supporting previous assumptions that growth rates of *E. coli* under aerobic conditions are limited by respiration and the concomitant rate of ATP generation through oxidative phosphorylation [12].

#### Amino acid biosynthesis and degradation

The most prominent differences in the proteome of cells growing exponentially in rich and glucose supplemented mineral salt medium are related to enzymes catalyzing the formation of amino acids from glucose and ammonia (and sulfate). Most striking is the exceptional strong abundance of MetE and IlvC, both proteins accumulating to 3.6 and 2.8%, respectively, of the relative protein mass (RPM) of *E. coli* BL21 growing in defined medium (Figure 2, Additional file 1: Table S1). Altogether, identified enzymes involved in amino acid synthesis (57 proteins) constitute almost 20% of the relative protein mass of cells growing in defined medium but accumulate only to 6% in the proteome of cells growing exponentially in rich medium (Figure 3). Thus, a large fraction of resources is directed to synthesize the enzymes required for amino acid synthesis during growth in glucose supplemented mineral salt medium while these resources can be redirected to other tasks in cells growing rapidly in rich medium.

In contrast, some enzymes required for amino acid salvage, e.g. enzymes required for the degradation of arginine, threonine, and proline (AstD, Tdh, Kbl, and PutA, respectively) are present in higher amounts during rapid growth in rich medium (Additional file 1: Table S1) suggesting that some amino acids are not only used for protein synthesis but also serve as precursors for other biomass building blocks or are even fed into central catabolic pathways.

For the detailed analysis of the abundance levels of the identified enzymes of the central carbon metabolism please refer to Additional file 1: Table S1.

### Transport systems

Substrates need to be transported into cells prior to their catabolic breakdown or employment for anabolic purposes. The most important transport system for carbohydrates, in particular glucose, is the phosphotransferase system (PTS). All identified enzymes of the PTS (e.g. PtsIH, Crr, and ManX) are present in larger amounts in cells growing in defined medium with glucose as carbon substrate compared to the cells growing in rich medium (Figure 4). In contrast, the proteins for maltose transport (MalEK and LamB) accumulate to higher levels in cells growing rapidly in rich medium (Figure 4). Interestingly, proteins involved in amino acid and peptide transport are present in larger amounts in cells growing in defined medium which does not contain amino acid and peptide supplements (Figure 4). The unspecific transport channels of the outer membrane (OmpA/F, OmpC absent in BL21 [13-15]) are present in similar amounts during exponential growth in all media (Additional file 1: Table S1).

For the detailed analysis of the abundance levels of the identified proteins involved in transport please refer to Additional file 1: Table S1.

### Protein synthesis and folding

**Figure 5:**
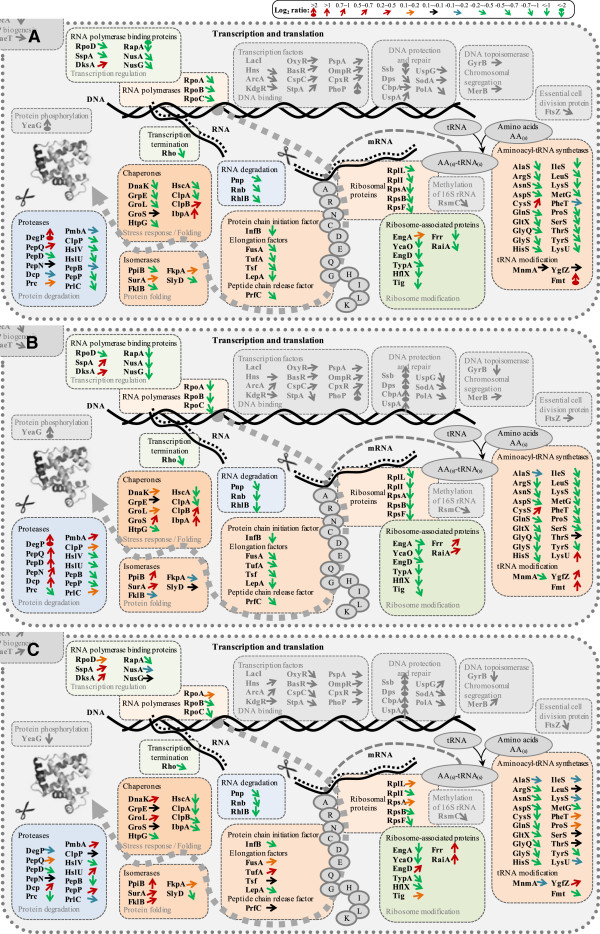
**The transcription, translation, and protein folding machinery: proteomic comparison at different growth conditions.** A detailed direct comparison of the transcription, translation, and protein folding machinery at the proteomic level at different growth conditions (**A**, defined versus rich media at exponential phase; **B**, stationary versus exponential phase in rich medium; and **C**, stationary versus exponential phase in defined medium) is given using different color coded arrows for the representation of the comparative Log_2_ ratios of single protein abundances [code on top, **A**: Log_2_(defined/rich), **B**: Log_2_(stationary/exponential)_rich_, **C**: Log_2_(stationary/exponential)_defined_]. An average of single protein abundances from cells grown in LB and TB media was calculated to represent the single protein abundance in cells grown in rich medium. The entire comparative proteomic (pathway) data are visualized in Additional file 1: Figures S7-S9 and the corresponding values are given in the Additional file 1: Table S1. The list of abbreviations is found at the end of the Additional file 1: Figure S9.

Proteins and enzymes involved in transcription, translation, and folding form a substantial part of the bacterial proteome under both growth conditions, however, they accumulate to larger quantities in cells growing exponentially in rich medium compared to the cells growing in defined medium (Figures 3 and 5, Additional file 1: Table S1). In particular, the fraction of proteins and enzymes directly related to translation, e.g. ribosomal proteins, aminoacyl-tRNA synthetases and elongation factors, is considerably higher in cells growing rapidly in rich medium (~20% versus ~12% of the relative protein mass, Figure 3 and Additional file 1: Table S1). Enzymes involved in transcription and most heat shock proteins chaperoning protein folding also accumulate to higher levels during exponential growth in rich medium but not as pronounced as those proteins directly involved in translation (Figure 3). However, chaperones of the heat shock protein family involved in aggregate dissolution (e.g. IbpA [16,17] and ClpB [18,19]) are present in lower amounts during growth in rich medium (Figure 5, Additional file 1: Table S1) reflecting a different level of regulation as for the majority of heat shock proteins. Other enzymes catalyzing protein folding reactions (e.g. peptidyl prolyl *cis-trans* isomerases) are present in relatively low amounts during exponential growth in both media (Additional file 1: Table S1) suggesting that these folding reactions could represent potential bottlenecks on the path to correctly folded protein chains.

For the detailed analysis of the abundance levels of the identified enzymes/proteins involved in protein synthesis and folding please refer to Additional file 1: Table S1.

### Stationary phase response

When entering into stationary phase, cells growing in rich and glucose supplemented mineral salt medium exhibit a different behavior, apparent through a gradual or a sudden decline of the growth rate (Figure 1), respectively, which is also reflected in different alterations of the cellular proteome (Figures 2 and 3, Additional file 1: Figure S10, Table S1). The proteome of cells growing in defined medium does not reveal big changes during entry into stationary phase (Figure 3, Additional file 1: Figure S10, Table S1), however, the proteome of cells growing in rich medium exhibits a more drastic change in composition (Figures 2 and 3, Additional file 1: Figure S10, Table S1).

#### Central (carbon) metabolism

The enzymes of the upper and lower glycolytic pathway, the pentose phosphate pathway and the amino acid synthesis pathways reveal a slight to moderate increase and the enzymes of the TCA cycle increase strongly in quantity during entry into stationary phase in rich medium (Figures 3 and 4). The pyruvate dehydrogenase, the multimeric enzyme connecting the glycolytic pathway and the TCA cycle, however, reveals a contrary behavior. AceE and AceF, subunits of pyruvate dehydrogenase complex, decrease considerably in quantity (Figure 4). Simultaneously, enzymes catalyzing other enzymatic conversions of pyruvate, e.g. from pyruvate to PEP (PpsA) or even to acetate (PoxB) and lactate (Dld and LdhA), increase in quantity (Figure 4). In addition, elevated amounts of Acs, the enzyme responsible for acetate utilization, and anaplerotic enzymes such as the glyoxylate shunt enzyme GlcB, and the enzymes catalyzing anaplerotic reactions between glycolysis and TCA cycle such as PckA and MaeB are observed (Figure 4). These findings show that during entry into stationary phase cells are getting attuned to metabolize acetate (*via* Acs) in agreement with the observed reutilization of acetate (Figure 1). A lower abundance of Pta and AckA in stationary phase cells (Figure 4) corroborates the previous finding that the Pta-AckA pathway is not utilized for acetate uptake but serves catabolic purposes leading to acetate secretion under carbon excess conditions [20,21]. Increased levels of PoxB and Acs and reduced levels of the pyruvate dehydrogenase in stationary phase cells (Figure 4) also suggest that a substantial fraction of a potential carbon flux from the glycolytic pathway to the TCA cycle may not pass through pyruvate dehydrogenase but through the PoxB and Acs dependent route.

#### Amino acid biosynthesis, transport systems, and salvage pathways

Enzymes of the amino acid biosynthesis pathways increase slightly on average during entry into stationary phase in rich medium but clearly do not reach the quantities found during growth in glucose-supplemented defined medium (Figures 2, 3 and 4, Additional file 1: Table S1). Most proteins of the PTS system, but also those involved in maltose, amino acid, and peptide transport, increase in quantity during entry into stationary phase in cells grown in rich medium (Figure 4, Additional file 1: Table S1). Also, the unspecifc transport channels of the outer membrane (OmpA/F) slightly increase in quantity (Additional file 1: Table S1) facilitating the influx of remaining substrates and preparing cells for potential future feast conditions. Moreover, enzymes utilized for amino acid degradation (e.g. for the degradation of tryptophan, TnaA; arginine, AstA/B/D; and threonine, Tdh and Kbl) increase in abundance during entry into stationary phase (Additional file 1: Table S1). In general, enzymes required for the uptake and degradation of less favorable substrates increase to a higher level during stationary phase.

#### Protein synthesis, degradation, folding, and stress responses

Proteins involved in transcription and translation show a strong decrease during entry into stationary phase in cells growing in rich medium (Figures 3 and 5, Additional file 1: Table S1). Most prominent is the severe decline of ribosomal proteins but also aminoacyl-tRNA-synthetases and enzymes involved in peptide chain elongation and RNA degradation decrease strongly in abundance. In contrast, heat shock proteins involved in chaperoning protein folding increase modestly and the disaggregation chaperones, ClpB and IbpA, as well as proteolytic enzymes increase strongly during entry into stationary phase in rich medium (Figure 5, Additional file 1: Table S1). These findings clearly emphasize that during entry into stationary phase cellular resources are redirected from proliferation to preservation by increasing the cellular potential to remove aggregated or otherwise damaged proteins by disaggregation and degradation and to reutilize released amino acids for the generation of protective enzymes. In this line, enzymes involved in the elimination of reactive oxygen species (e.g. SodB, KatG), DNA protection (e.g. Ssb, Dps, CbpA), and general stress responses (UspA) also show a significant increase during entry into stationary phase (Additional file 1: Table S1). Altogether, the changes observed during entry into stationary phase in cells growing in rich medium in the abundance of enzymes related to central carbon metabolism, transport systems and salvage pathways, protein synthesis and folding as well as stress responses are also observed for cells entering stationary phase during growth in glucose-supplemented mineral salt medium but not as pronounced (Figure 3 and 5, Additional file 1: Table S1). During the slower exponential growth in the defined medium cells already exhibit a more “stationary-phase-like” phenotype compared to the rapidly growing cells in rich medium and thus, the reconstruction of the proteome during entry into stationary phase during growth in glucose-supplemented mineral salt medium is not that prominent (Additional file 1: Figure S10).

For the detailed analysis of the abundance levels of all identified proteins in cells from exponential and stationary phase in defined and rich media please refer to Additional file 1: Table S1.

## Discussion

Our studies illustrate that the most significant alterations in the proteome of *E. coli* BL21 at the different growth conditions studied are related to the enzymes of the central metabolic pathways, amino acid biosynthesis, the translational machinery, and general cell protection (Table 1).

**Table 1:** Compendium of quantitative proteomic changes at different growth conditions

**Group**^**1**^		**Hunger**	**Feast**	**Famine**	**Transcriptional regulation**^**2**^		
		**Mineral salt medium at exponential phase**	**Rich medium at exponential phase**	**Rich medium at stationary phase**			
**Protein**^**1**^	**Function**^**1**^	**Relative Protein Mass (RPM)**^**1 **^**-%**			**Activation**	**Repression**	**Sigma factor**
**Upper glycolysis**							
FbaB	FBP → DHAP + G3P	**0.03**	0.00	**0.08**		**Cra**	**RpoS**
TpiA	DHAP → G3P	**0.26**	0.21	**0.39**		**Cra**	**RpoS**, RpoD
**Lower glycolysis**							
GapA	G3P → 1,3-BPG	0.70	**0.97**	**1.23**	**CRP-cAMP**	Cra	**RpoS**, RpoD
GpmA	3PG → 2PG	0.27	**0.38**	**0.62**		Fur	**RpoS**, RpoD
**Pyruvate dehydrogenase**							
AceE	Pyruvate → Acetyl-CoA + CO_2_	0.82	**2.15**	1.49	**CRP-cAMP**	Cra, **PdhR**	RpoS, RpoD
AceF		0.34	**0.82**	0.58	**CRP-cAMP**	Cra, **PdhR**	RpoS, RpoD
**Acetate metabolism**							
Acs	Acetate → Acetyl-CoA	0.10	0.06	**0.35**	CRP-cAMP	**Fis**	**RpoS**, RpoD
PoxB	Pyruvate → Acetate + CO_2_	0.02	0.02	**0.08**			**RpoS**, RpoD
Pta	Acetyl-CoA → Acetyl-Phosphate	0.14	**0.35**	0.17	**ArcA-P**		
AckA	Acetyl-Phosphate → Acetate	0.12	**0.36**	0.15	**ArcA-P**		RpoH
**TCA cycle**							
GltA	Oxaloacetate + Acetyl-CoA → Citrate	**1.12**	0.29	**1.25**	CRP-cAMP	**ArcA-P**	RpoD
AcnB	Citrate → Isocitrate	**0.88**	0.60	**1.06**	CRP-cAMP	**ArcA-P**, Fis	RpoD
SucD	Succinyl-CoA → Succinate	**0.49**	0.22	**0.60**	CRP-cAMP	**ArcA-P**	**RpoS**, RpoD
SdhA	Succinate → Fumarate	**0.64**	0.26	**0.59**	CRP-cAMP	**ArcA-P**	RpoD
FumA	Fumarate → (S)-Malate	**0.07**	0.03	**0.10**	CRP-cAMP	**ArcA-P**	RpoD
**Amino acid biosynthesis**							
IlvC	Branched-chain amino acids biosynthesis	**2.83**	0.16	0.13		**Leucine**^3^	RpoD
MetE	Methionine biosynthesis	**3.60**	0.10	0.16		**Methionine**^3^	
CysK	Cysteine biosynthesis	**0.67**	0.19	0.45		**Cysteine**^3^	RpoD
ThrB	Threonine biosynthesis	**0.08**	0.03	0.06	**DksA-ppGpp**		RpoD
HisC	Histidine biosynthesis	**0.09**	0.00	0.04	**DksA-ppGpp**		RpoD
**RNA polymerases**							
RpoA	RNA polymerase, core enzyme	0.51	**0.74**	0.37		**DksA-ppGpp**	RpoD
**Ribosomal proteins**							
RplL	50S ribosomal subunit protein	0.70	**0.95**	0.53		**DksA-ppGpp**	
RpsA	30S ribosomal subunit protein	0.86	**2.11**	0.88		**DksA-ppGpp**	RpoD
**Elongation factors**							
FusA	Elongation factor G	1.49	**2.18**	1.39		**DksA-ppGpp**	RpoD, RpoE
TufA	Elongation factor Tu (EF-Tu)	3.51	**5.66**	3.98		**DksA-ppGpp**	RpoD, RpoE
**Amino acid degradation**							
AstA	Arginine (to succinate) degradation	0.03	0.00	**0.10**			**RpoS**
AstB		0.03	0.03	**0.06**			**RpoS**
**(Metabolite) degradation**							
GabD	4-Aminobutyrate (GABA) degradation	0.10	0.09	**0.17**	CRP-cAMP		**RpoS**
GabT		0.00	0.00	**0.10**	CRP-cAMP		**RpoS**
TreA	Trehalose degradation	0.06	0.03	**0.05**			**RpoS**
**Hydroperoxide reductases and superoxide dismutase**							
Tpx	Antioxidant under anaerobic condition	0.08	0.06	**0.17**		**ArcA-P**	RpoD
SodB	Superoxide dismutase	0.14	0.12	**0.91**		**CRP-cAMP**	RpoD
**DNA protection and repair**							
Dps	Protection of the DNA from damage	0.03	0.05	**0.65**		**Fis**	**RpoS**
UspA	A member of the RecA-dependent DNA protection and repair system	0.11	0.07	**0.20**		**FadR**	RpoD

^1^Functional classification is mostly according to EcoCyc database (http://ecocyc.org/) and confirmed by KEGG database (http://www.genome.jp/kegg/). Only examples from important groups are given the detailed analysis of the abundance levels of all identified proteins is found in Additional file 1: Table S1 or Additional file 2: Table S5 for the entire data set including standard deviation. The RPM (%) of up-regulated proteins in bold letters for better recognition.

^2^The information of transcriptional regulation is from RegulonDB Version 7.0 (http://regulondb.ccg.unam.mx). According to the differences of each protein’s Relative Protein Mass at hunger, feast and famine conditions, the most (feasible) dominating transcription and sigma factors controlling the expression of the corresponding gene are given in bold letters.

^3^Feed-back inhibition of amino acid biosynthesis (enzymes) by the end products of the corresponding pathways.

### Antagonistic regulation of central metabolic pathways and acetate formation

**Figure 6:**
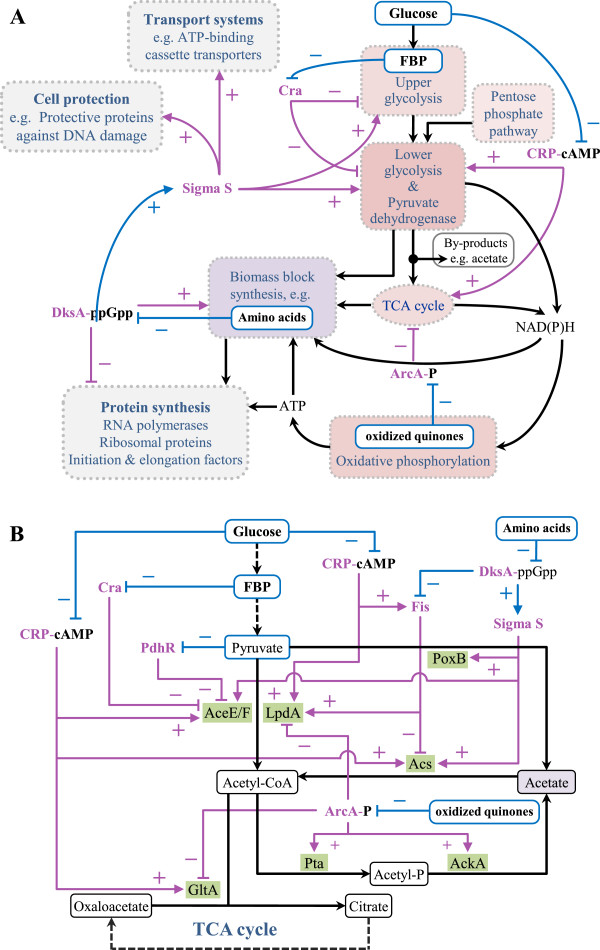
**Simplified model of transcriptional regulation of central metabolic pathways and cellular activities.** General overview **(A)** and details of the acetate pathway **(B)**. The flow of metabolites including energy currency metabolites such as NAD(P)H and ATP is indicated in black. Pathway regulation by transcription and sigma factors is indicated in pink: activation (+, line ending arrow) and inhibition (−, line ending bar). Regulation of transcription and sigma factors by metabolites and low molecular weight signal molecules is indicated in blue: activation/stabilisation (+, line ending arrow) and deactivation (−, line ending bar). The list of abbreviations is found at the end of the Additional file 1: Figure S9. The construction of this simplified model of transcriptional regulation is based on data found in RegulonDB (http://regulondb.ccg.unam.mx/) [58].

Although studied for decades, acetate formation is still a puzzling peculiarity of *E. coli* carbon metabolism. *E. coli* is a “Crabtree-positive” bacterium secreting acetate not only under conditions of oxygen limitation but also under “carbon excess” conditions presumably as a result of an unbalanced coupling of catabolic and anabolic pathways. Many transcriptional factors are involved in the regulation of central metabolic pathways (Figure 6, Additional file 1: Table S2), but the predominant ones are the catabolite repressor activator (Cra or FruR) [22], the cAMP receptor protein (CRP) [23], and the aerobic respiration control protein (ArcA) [24,25]. These global transcription factors are known to have opposing effects on the expression of metabolic pathway genes. For example, Cra causes repression of the majority of glycolytic pathway genes [26] as well as the genes of the pyruvate dehydrogenase complex [22] (Figure 6, Additional file 1: Table S2). Inactivation of Cra is caused by high levels of fructose-1-phosphate (F1P) or fructose-1,6-bisphosphate (FBP) [27,28] leading, most likely, to elevated levels of the respective enzymes. On the other hand, high levels of glucose are known to cause low CRP-cAMP levels [29] and, thus, expression of genes dependent on CRP-cAMP, e.g. the first gene of the lower glycolytic pathway, namely *gapA*[30]*,* and the genes of the pyruvate dehydrogenase complex [31,32] will be low. Thus, the corresponding enzymes may exhibit opposing changes in their abundance than the majority of glycolytic pathway enzymes when cells are subjected to a change in growth conditions. CRP-cAMP does not only activate *gapA* and the genes of the pyruvate dehydrogenase complex but also the genes of the TCA cycle [33-36] which are additionally controlled by ArcA [25] (Figure 6, Additional file 1: Table S2). ArcA control on TCA cycle genes is mediated by the pool of oxidized quinones; a high level of oxidized quinones prevents phosphorylation of ArcA which in turn relieves repression of TCA cycle genes [24]. Opposite, a high level of reduced quinones leads to phosphorylation of ArcA and resultant repression of TCA cycle genes. Thus, limitations in the respiratory pathway, in particular in the reoxidation of quinones by terminal oxidases, and high rates of NAD(P)H generation may shift the equilibrium to reduced quinones and may cause a downregulation of the TCA cycle genes and a resulting decrease in the abundance of TCA cycle enzymes.

According to our study, the formation of acetate, or more general, the observed regulation of central metabolic pathways, appears to result mainly from the opposing effects of these three different transcription factors. During exponential growth in glucose-supplemented mineral salt medium high levels of upper glycolytic and low levels of lower glycolytic pathway enzymes as well as low levels of the enzymes of the pyruvate dehydrogenase complex are observed. The opposite is true for exponential growth in rich medium, under these conditions upper glycolytic pathway enzymes show a slight decrease while lower glycolytic pathway enzymes and the enzymes of the pyruvate dehydrogenase complex strongly increase in abundance. Thus, during exponential growth in rich medium a repressing influence of Cra on the lower glycolytic pathway as well as on the genes of the pyruvate dehydrogenase complex might be outcompeted by an activating influence of CRP-cAMP leading to increased levels of the corresponding enzymes, e.g. to an increased level of the pyruvate dehydrogenase complex (Figure 4, Table 1). On the other hand decreased levels of TCA cycle enzymes during rapid growth in rich medium indicate that the activating influence of CRP-cAMP on the TCA cycle genes is outcompeted by a repressing influence of high levels of phosphorylated ArcA (ArcA-P) (Figure 4, Table 1, Additional file 1: Table S2). Thus, the dominating role of ArcA-P over CRP-cAMP most likely leads to the observed unbalance at the junction of the glycolytic pathway and the TCA cycle in cells growing rapidly in rich medium (Figure 4, Table 1). In summary, the observed differences in the abundance of enzymes of the central metabolic pathways can be explained by the antagonistic and competitive pathway regulation through these three global transcriptional regulators, Cra, CRP-cAMP, and ArcA-P: Cra inhibits the expression of genes of the upper and lower glycolytic pathway and the genes of the pyruvate dehydrogenase complex, CRP-cAMP activates the expression of genes of the lower glycolytic pathway (e.g. *gapA*), the pyruvate dehydrogenase complex, and the TCA cycle, and ArcA-P inhibits the expression of TCA cycle genes (Figure 6, Additional file 1: Table S2). In nutrient excess conditions, ArcA-P appears to be the dominating transcription factor leading to the well-known bottleneck at the pyruvate node, the junction between the glycolytic pathway and the TCA cycle, and the observed off-pathway carbon flux towards acetate (Figures 1 and 4).

### More details about the pyruvate node and acetate dilemma

Starting from pyruvate, the formation of acetate can occur via two different routes (Figure 6B). The most important route involves the pyruvate dehydrogenase catalyzed generation of acetyl-CoA followed by the formation of acetyl-phosphate and, finally, acetate, involving the two enzymes phosphotransacetylase (Pta) and acetate kinase (AckA), the Pta-AckA pathway [20]. Regulation of genes encoding the enzymes of the pyruvate dehydrogenase is complex and involves many factors (Figure 6B, Additional file 1: Table S2). In addition to regulation through Cra and CRP-cAMP, the pyruvate dehydrogenase transcriptional repressor (PdhR) mediated repression of *aceEF* is inhibited by elevated concentrations of pyruvate [31,37] (Figure 6B, Additional file 1: Table S2) presumably leading to increased levels of pyruvate dehydrogenase. Little is known about the regulation of the following Pta-AckA pathway, amongst others, transcriptional control seems to be mainly exerted through Fnr [38] (no functional *fnr* gene in *E. coli* BL21(DE3) [39]) but also, at least moderately, through ArcA-(P) [38,40] (Figure 6B). The other, though minor route towards acetate is directly from pyruvate catalyzed by pyruvate oxidase B (PoxB) [21,41]. The Pta-AckA pathway is the dominating pathway in the exponential growth phase, and the PoxB pathway dominates in the stationary phase [42]. PoxB is a unidirectional acting enzyme [43,44], but the Pta-AckA pathway can, in principle, operate in both directions [20]. Acetate uptake, however, occurs almost exclusively through acetyl coenzyme A synthetase (Acs) [20,45]. *Acs* expression is controlled by many factors [46] (Figure 6B) including Fis, a nucleoid associated protein [47,48] which accumulates to high levels during rapid growth in rich medium [49,50]. Fis is the most important factor negatively controlling *acs* expression during exponential growth [46]. It has been suggested that acetate accumulation at high growth rates occurs mainly as a result of the reduced assimilation capacity due to the downregulation of Acs [51]. On the other hand, increased *acs* expression occurs during entry into stationary phase [52] and, moreover, increased *acs* expression as well as elevated Acs levels are observed at low growth rates in continuous culture experiments [51,53].

During rapid growth in rich medium we observed a significant increase in the abundance of the subunits of the pyruvate dehydrogenase complex (AceEF) which does not match with the more moderate increase observed for the lower glycolytic pathway enzymes (Figure 4, Table 1, Additional file 1: Table S1). Most likely, in addition to the CRP-cAMP dependent activation elevated levels of intracellular pyruvate additionally contribute through the inhibition of PdhR to elevated expression of the genes of the pyruvate dehydrogenase complex, respectively to an increased abundance of the corresponding subunits. These findings suggest that in rich medium carbon overflow does not only cause limitations at the end of the catabolic pathways, e.g. in reoxidizing reduced quinones, but also upstream, e.g. at the end of the glycolytic pathway by accumulation of pyruvate. Remarkably, cells do not activate pyruvate catabolism through PoxB but try to enhance the capacity for pyruvate catabolism through pyruvate dehydrogenase during rapid growth in rich medium (Figure 4B, Table 1). Thus, formation of acetate from pyruvate obviously involves the pyruvate dehydrogenase catalyzed reaction and the Pta-AckA pathway and not the PoxB dependent route. The observed low levels of TCA cycle enzymes as well as the strong abundance of Pta and AckA under these growth conditions (Figure 4, Table 1, Additional file 1: Table S1) pave the route towards acetate formation and are consistent with a dominating role of the transcriptional regulator ArcA-P (Figure 6). Thus, our results strongly suggest that acetate formation – even in the low acetate-producing strain BL21 – is caused by limitations in the respiratory chain, e.g. a limited capacity to reoxidize reduced quinones. The observed almost neglectable amounts of Acs during rapid growth in rich medium (Figures 2 and 4, Table 1, Additional file 1: Table S1), presumably caused by high levels of Fis, additionally contribute to the observed extracellular accumulation of acetate (Figure 1). Another important issue to consider is the high level of freely available amino acids in rich medium as they additionally contribute to the overload on the lower glycolytic pathway and the pyruvate node because there is no need for flux diversion towards amino acid biosynthesis. Indeed, all enzymes which catalyze the first reactions of amino acid biosynthesis and the majority of the following enzymes starting from glycolytic pathway as well as form TCA cycle intermediates are strongly down-regulated during exponential growth in rich medium (Table 1, Additional file 1: Figure S7, Table S1). Thus, acetate formation during rapid growth in rich medium is additionally aggravated through the availability of free amino acids.

### Stringent-like and stationary phase responses

Absence or low levels of free amino acids are known to provoke a stringent-like response by elevating the amounts of the signaling molecule(s) (p)ppGpp [54-56]. (p)ppGpp binds to RNA polymerase (RNAP) and in conjunction with the RNAP binding protein DksA downregulates the expression of genes encoding ribosomal proteins [57] and other enzymes and proteins involved in protein synthesis [57,58] (Figure 6, Additional file 1: Table S3). Moreover, ribosomal RNA and ribosomal proteins are also being actively degraded during entry into stationary phase [59-61]. ppGpp exerted control is proposed to occur in a gradual manner: freely available amino acids result in low ppGpp concentrations (feast conditions), no free amino acids available but opportunity for amino acid biosynthesis (hunger conditions) lead to intermediate ppGpp concentrations, and no free amino acids and no opportunity for amino acid biosynthesis (famine conditions) result in the accumulation of high levels of ppGpp [56]. Conditions between feast and famine, e.g. hunger provoking conditions are also known to initiate the induction of biosynthetic pathways [56]. The induction of some pathways involved in amino acid biosynthesis by DksA-ppGpp has been shown (e.g. *thrB*, *hisC*) [62-64], although the majority of amino acid biosynthesis pathways are controlled by complex feedback inhibition mechanisms, including the transcriptional regulation of *metE*[65], *ilvC*[66-68], and *cysK*[69-71]. ppGpp bound to DksA also induces the expression of the stationary phase sigma factor (RpoS or s^σ^) [72], however, the induction of the RpoS regulon requires high ppGpp concentrations, namely famine conditions [56]. RpoS controls the expression of many stationary phase genes including the genes encoding transport proteins for better nutrient scavenging [73,74] and degradation of less favorable substrates [75-79] as well as genes of the oxidative stress response [56] and other genes related to general cell protection [80] (Additional file 1: Table S4). RpoS also exerts positive control on the expression of metabolic pathway genes, namely the genes of the lower and upper glycolytic pathway [56,81] and, importantly, also on *acs*[52,81,82] and *poxB*[56,81,83] (Figure 6, Additional file 1: Table S4). Moreover, *acs* expression receives additional amplification during entry into stationary phase as the negative control of Fis on *acs* in exponentially growing cells is reversed through the negative control of Fis through DksA-ppGpp [84] (Figure 6, Additional file 1: Table S3).

In the experimental conditions investigated in this study, feast, hunger, and famine conditions are encountered during exponential growth in rich medium, exponential growth in glucose supplemented defined mineral salt medium, and in stationary phase, respectively, and reflected in the observed alterations of the bacterial proteome of *E. coli* BL21 (Table 1). Proteins of the translational machinery reach their highest abundance during rapid growth in rich medium, intermediate levels at exponential growth in glucose-supplemented mineral salt medium, and lowest levels during stationary phase (Figures 3 and 5, Table 1, Additional file 1: Table S1). The high levels of enzymes of the amino acid synthesis pathway found during growth on glucose-supplemented defined mineral salt medium (Figures 2 and 3, Table 1, Additional file 1: Table S1) are in line with hunger conditions known to provoke the induction of biosynthetic pathways. However, the exceptionally high levels of MetE and IlvC found as part of the *E. coli* BL21 proteome during growth on glucose supplemented defined mineral salt medium (Figure 2A and Table 1) are presumably mainly caused by feedback derepression of amino acid synthesis pathways. The RpoS controlled response is most obviously reflected in the alterations of the proteome of *E. coli* BL21 after transition from exponential growth to stationary phase in rich medium. For example, the observed increase in proteins devoted to degradation of less favorable substrates (e.g. AstA/B, GabD/T and TreA) and general cell protection (e.g. DpS) (Table 1, Additional file 1: Figures S8 and S9, Table S1) is consistent with the expected RpoS dependent alterations of the bacterial proteome of stationary phase cells. In addition, the control of central metabolic pathways through RpoS becomes also evident through the general increase in the abundance of glycolytic pathway enzymes but also in the increased levels of PoxB and Acs in stationary phase cells (Figures 2, 4 and 6, Table 1, Additional file 1: Table S1) and the concurrent uptake of acetate during stationary phase (Figure 1). However, there are also other proteins important for stationary phase survival of *E. coli* with increasing abundance during stationary phase in rich medium whose genes are not controlled by RpoS but by other factors (e.g. SodB [32], UspA [85], Tpx [86], SsB [87]) (Table 1, Additional file 1: Figure S8, Table S1). Interestingly, the proteomic reconstruction during entry into stationary phase is more prominent in cells growing in rich medium compared to cells growing in glucose-supplemented mineral salt medium. It seems the large amount of ribosomal proteins and other proteins involved in protein synthesis present during rapid growth in rich medium serve as the major reservoir for recycling to generate the proteins required for starvation survival. The lower abundance of ribosomal proteins in exponentially growing cells in glucose-supplemented mineral salt medium represents a more limited pool for proteomic reconstruction during entry into stationary phase. The large portion of proteins involved in amino acid biosynthesis in exponentially growing cells from defined medium does not serve as recycling reservoir as these proteins do not decrease during entry into stationary phase but slightly increase in quantity.

## Conclusions

In summary the observed changes in the proteome during entry into stationary phase are consistent with stringent-like and stationary phase responses and a dominating control through DksA-ppGpp and RpoS. The proteomic changes during entry into stationary phase are more prominent in rich medium as the cells have access to a larger reservoir of recyclable proteins, e.g. the proteins of the translational machinery. During rapid growth in rich medium ArcA-P appears to be the dominant metabolic pathway controlling transcriptional factor most likely the result of the limited capacity of *E. coli* to reoxidize the reduced quinones leading to the well-known phenomenon of acetate secretion. Certainly, transcriptional regulation is more complex than outlined above but these simplified interpretations adequately address the observed compositional alterations of the *E. coli* BL21 proteome. It seems also that the observed regulatory pattern is a good example of combinatorial control which can be suboptimal for cells under certain growth conditions [88] in particular under laboratory conditions which may substantially deviate from the conditions of the ecological niche in which the cells have originally evolved.

## Materials and methods

### Strain, culture conditions, and basic analytical procedures

*E. coli* BL21 (DE3) (Novagen, Germany) was grown in rich (Luria Bertani or Terrific Broth) or defined mineral salt medium with glucose as carbon source. The composition of the rich media was as follows: Luria Bertani (LB): 10 g L^−1^ tryptone (Becton, Dickinson and Company-BD, USA), 5 g L^−1^ yeast extract (Becton, Dickinson and Company-BD, USA), and 5 g L^−1^ NaCl; Terrific broth (TB): 12 g L^−1^ tryptone (Becton, Dickinson and Company-BD, USA), 24 g L^−1^ yeast extract (Becton, Dickinson and Company-BD, USA), 5 g L^−1^ glycerol, 2.31 g L^−1^ KH_2_PO_4_, and 12.54 g L^−1^ K_2_HPO_4_. The composition of the Defined Non-inducing Broth (DNB) was as follows: 10.91 g L^−1^ glucose, 4 g L^−1^ (NH_4_)_2_HPO_4_, 13.3 g L^−1^ KH_2_PO_4_, 1.55 g L^−1^ citric acid, 0.59 g L^−1^ MgSO_4_, 100.8 mg L^−1^ Fe(III) citrate, 2.1 mg L^−1^ Na_2_MoO_4_.2H_2_O, 2.5 mg L^−1^ CoCl_2_.6H_2_O, 15 mg L^−1^ MnCl_2_.4H_2_O, 1.5 mg L^−1^ CuCl_2_.2H_2_O, 3 mg L^−1^ H_3_BO_3_, 33.8 mg L^−1^ Zn(CH_3_COOH)_2_.2H_2_O, 14.1 mg L^−1^ Titriplex III. The pH of all media was adjusted to pH 6.8 with NaOH prior to sterilization. Details about medium preparation were as described previously [3,89,90]. Cultivations were carried out in duplicate using 2 L Erlenmeyer flasks with three baffles containing 200 ml medium at 37°C and 180 rpm (Multitron Cell, INFORS HT, Switzerland). Cell growth was monitored by measurement of the absorbance at 600 nm (WPA CO8000 Cell Density Meter, Biochrom, UK). Acetate was analyzed using an acetic acid kit (Cat. No. K-ACETRM, Megazyme, Ireland). Cell samples for two-dimensional (2D) gel electrophoresis were immediately centrifuged at 17,000 g and 4°C for 3 min after sampling. After removal of the supernatant, cell pellets were stored at −80°C prior to gel electrophoresis. The profile of the dissolved oxygen concentration was determined in 500 mL shake flasks with integrated pH and oxygen sensors (SFS) using a SFR Shake Flask Reader (PreSens Precision Sensing GmbH, Germany) and the same cultivation conditions as described above (50 mL medium at 37°C and 180 rpm). Offline sampling was omitted in shake flask cultures during dissolved oxygen measurements to prevent perturbation of the dissolved oxygen profile.

### 2D gel electrophoresis

Cell pellets were disrupted using BugBuster™ Protein Extraction Reagent (Novagen, USA) supplemented with rLysozyme and Benzonase according to manufacturer's instructions. Whole cell protein in the BugBuster suspension was precipitated without prior centrifugation as described previously [91]. The protein pellets were solubilized in rehydration solution with IPG buffer (GE Healthcare, UK). About 280 μg of protein were loaded onto Immobiline DryStrip gels of pH 3–10 NL (GE Healthcare, UK). The first-dimension using isoelectric focusing (IEF) and the second dimension using SDS-PAGE (10-15% gradient gel) were carried out using the IPGphor™ Isoelectric Focusing and Hoefer™ DALT systems (GE Healthcare, UK), respectively. The detailed experimental conditions were according to manufacturer's instructions and as described previously [92]. Gels were stained using colloidal Coomassie R250 [93] and analyzed using Proteomweaver™ 4.0 software (Bio-Rad, USA) for protein spot detection, matching, and quantification. For each sample, 2D gels were made in triplicate and the best two gels analyzed using Proteomweaver™ 4.0. Each spot’s intensity was normalized by the whole spots intensity of the same 2D gel. The corresponding average intensity of the spot (or the sum of several spots representing the same protein in case of spot multiplicity) taken from the two duplicate gels was used to determine this protein’s portion (%) of the relative protein mass (RPM).

### Protein identification and classification

Protein spots were identified by matrix-assisted laser desorption ionization time-of-flight mass spectrometry (MALDI-TOF MS). Protein spots were excised manually from 2D gels. After washing, reduction, and alkylation, in-gel digestion was carried out by incubation with sequencing grade porcine trypsin modified by reductive methylation (Promega, USA). Obtained peptides were extracted and purified with reversed-phase C18 ZipTips (Millipore, USA). Desalted peptide solutions were mixed with a saturated matrix solution and spotted onto a 384 MTP target and dried at room temperature. A Bruker Ultraflex time-of-flight mass spectrometer (Bruker Daltonics GmbH, Germany) was employed to obtain peptide mass fingerprints. Details of the protocol are given elsewhere [92,94]. The MASCOT search program (Matrix Science, UK) was used for protein identification using the annotated *E. coli* genome as database (Uniprot: http://www.uniprot.org/). All proteins with a Mowse score greater than 54 were regarded as significant (p < 0.05). The EcoCyc database was mainly used for classification of identified proteins into functional categories (http://ecocyc.org/, [95]). Classification was confirmed using the KEGG database (http://www.genome.jp/kegg/, [96]). The transcriptional regulation of genes encoding identified proteins were mainly acquired from the RegulonDB (http://regulondb.ccg.unam.mx/, [58]) and EcoCyc (http://ecocyc.org/, [95]) databases.

## Competing interests

The authors declare that they have no competing interests.

## Authors’ contributions

ZL did the experimental work, analyzed the data and prepared the first draft of the manuscript. MN contributed to protein identification by Maldi-ToF. UR directed the study and prepared the final manuscript. All authors read and approved the final manuscript.

## Supplementary Material

Additional file 1 **Figures S1-S6.** Images of 2D gels of the proteome of *E. coli* BL21 (DE3) growing in defined and rich media (Luria Bertani and Terrific Broth) at exponential and stationary growth phases. Identified proteins are marked and “clickable” to get access to further protein information (http://www.uniprot.org). **Figures S7-S9**: Detailed comparative scheme of the (proteomic) pathway regulation at different growth conditions. *E. coli* BL21 (DE3) growing in defined medium versus rich medium at exponential phase, at stationary phase versus exponential phase in rich medium, and at stationary phase versus exponential phase in defined medium. **Figure S10**: Comparison of the stationary and exponential phase proteome of *E. coli* BL21 (DE3) in different media. **Table S1**: Quantitative data of individual proteins of *E. coli* BL21 (DE3) growing in defined and rich media (Luria Bertani and Terrific Broth) at exponential and stationary phases. **Table S2**: Transcriptional control of glycolysis and TCA cycle genes. The information was extracted from the RegulonDB database (http://regulondb.ccg.unam.mx/). **Table S3**: DksA-ppGpp controlled genes of *E. coli*. The information was extracted from the RegulonDB (http://regulondb.ccg.unam.mx/) and EcoCyc databases (http://ecocyc.org/). **Table S4**: RpoS controlled genes of *E. coli*. The information was extracted from the RegulonDB (http://regulondb.ccg.unam.mx/) and EcoCyc databases (http://ecocyc.org/). Click here for file

Additional file 2: Table S5 Quantitative data of individual proteins (obtained from duplicate gels showing standard deviation) of *E. coli* BL21 (DE3) growing in defined and rich media (Luria Bertani and Terrific Broth) at exponential and stationary phases. Click here for file
