# Supplementary material for: The metabolic potential of Escherichia coli BL21 in defined and rich medium
Source: Microb Cell Fact. 2014 Mar 23;13:45. doi: 10.1186/1475-2859-13-45 (PMC4021462; doi:10.1186/1475-2859-13-45)
Supplement: Additional file 1 — Figures S1-S6. Images of 2D gels of the proteome of E. coli BL21 (DE3) growing in defined and rich media (Luria Bertani and Terrific Broth) at exponential and stationary growth phases. Identified proteins are marked and “clickable” to get access to further protein information (http://www.uniprot.org). Figures S7-S9: Detailed comparative scheme of the (proteomic) pathway regulation at different growth conditions. E. coli BL21 (DE3) growing in defined medium versus rich medium at exponential phase, at stationary phase versus exponential phase in rich medium, and at stationary phase versus exponential phase in defined medium. Figure S10: Comparison of the stationary and exponential phase proteome of E. coli BL21 (DE3) in different media. Table S1: Quantitative data of individual proteins of E. coli BL21 (DE3) growing in defined and rich media (Luria Bertani and Terrific Broth) at exponential and stationary phases. Table S2: Transcriptional control of glycolysis and TCA cycle genes. The information was extracted from the RegulonDB database (http://regulondb.ccg.unam.mx/). Table S3: DksA-ppGpp controlled genes of E. coli. The information was extracted from the RegulonDB (http://regulondb.ccg.unam.mx/) and EcoCyc databases (http://ecocyc.org/). Table S4: RpoS controlled genes of E. coli. The information was extracted from the RegulonDB (http://regulondb.ccg.unam.mx/) and EcoCyc databases (http://ecocyc.org/). [file 1475-2859-13-45-S1.pdf]

Additional file 1

The metabolic potential of *Escherichia coli* BL21 in defined and rich medium

Zhaopeng Li[1,2], Manfred Nimtz[1] and Ursula Rinas[1,2*]

1 Helmholtz Centre for Infection Research, Braunschweig, Germany

2 Leibniz University of Hannover, Technical Chemistry – Life Science, Hannover, Germany

*Corresponding author. Ursula.Rinas@helmholtz-hzi.de

Table of Contents

Additional Figures

Clickable 2D gels — Page

Figure S1 - 2D gel of *E. coli* BL21(DE3) growing in DNB medium at exponential phase .... [**2**]

Figure S2 - 2D gel of *E. coli* BL21(DE3) growing in DNB medium at stationary phase .... [**3**]

Figure S3 - 2D gel of *E. coli* BL21(DE3) growing in LB medium at exponential phase .... [**4**]

Figure S4 - 2D gel of *E. coli* BL21(DE3) growing in LB medium at stationary phase .... [**5**]

Figure S5 - 2D gel of *E. coli* BL21(DE3) growing in TB medium at exponential phase .... [**6**]

Figure S6 - 2D gel of *E. coli* BL21(DE3) growing in TB medium at stationary phase .... [**7**]

Comparative proteome analysis of *E. coli*

Figure S7 - Exponential phase in defined medium versus exponential phase in rich medium .... [**8**]

Figure S8 - Stationary phase versus exponential phase in rich medium .... [**9**]

Figure S9 - Stationary phase versus exponential phase in defined medium .... [**10**]

Figure captions for Figures S7, S8 and S9 .... **[11]**

Abbreviations used in Figures S7, S8 and S9 .... [**11**]

Figure S10 - Comparison of the stationary and exponential phase proteome of *E. coli* BL21 (DE3) in different media .... [**14**]

Additional Tables

Table S1 - Quantitative data of individual proteins of *E. coli* BL21 (DE3) growing in defined and rich media .... [**15**]

Table S1.1 - Summary of quantitative data of individual proteins of *E. coli* BL21 (DE3) growing in defined and rich media .... [**35**]

Table S2 - Transcriptional control of glycolysis and TCA cycle genes .... [**37**]

Table S3 - DksA-ppGpp controlled genes of *E. coli* .... [**41**]

Table S4 - RpoS controlled genes of *E. coli* .... [**44**]

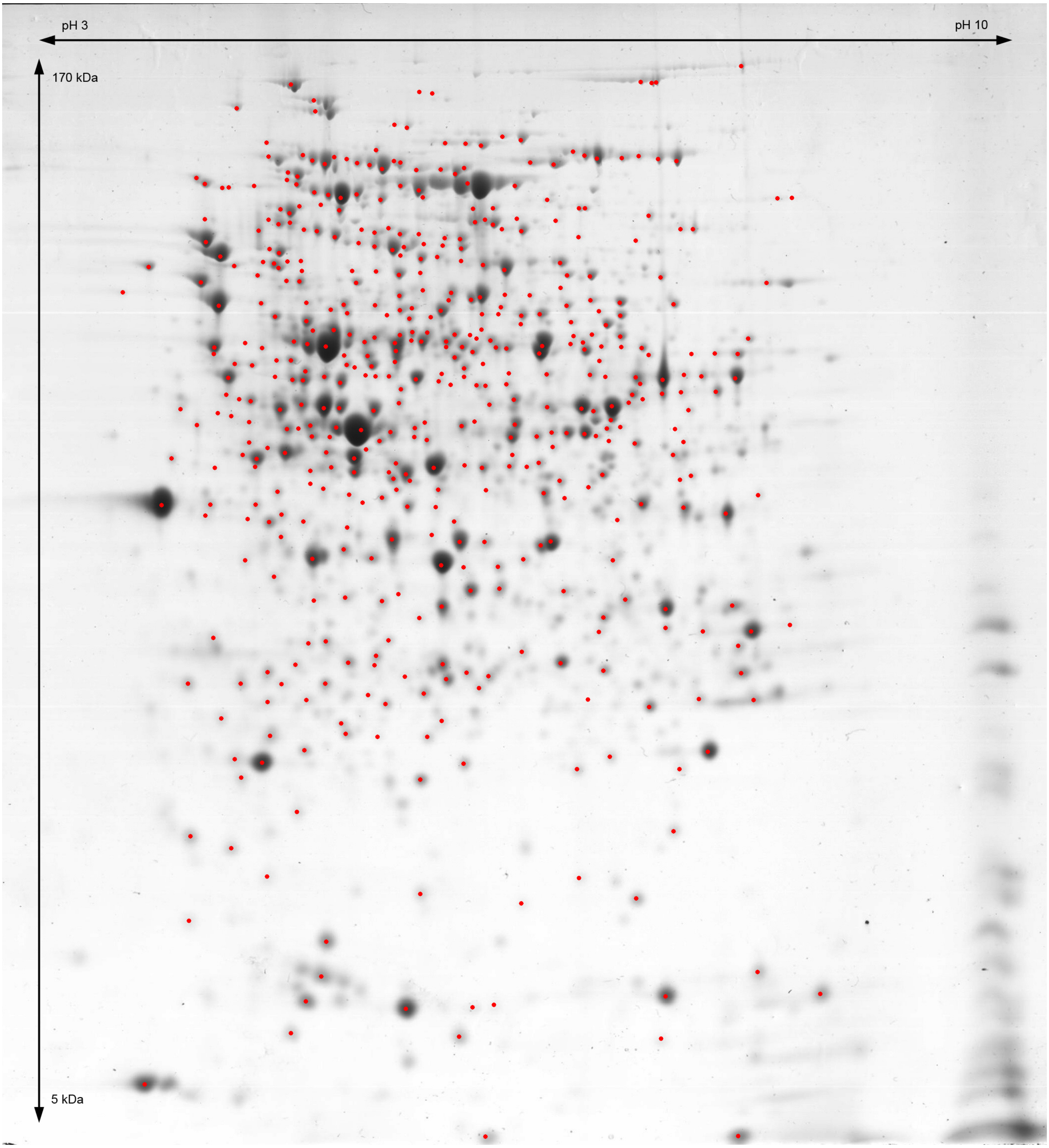

Figure S1 - 2D gel of *E. coli* BL21(DE3) growing in DNB medium at exponential phase

Click on the red spots to achieve detailed information of identified spots (proteins). Cultivation was carried out in shaker flask with baffles at 37 ºC and 200 rpm.

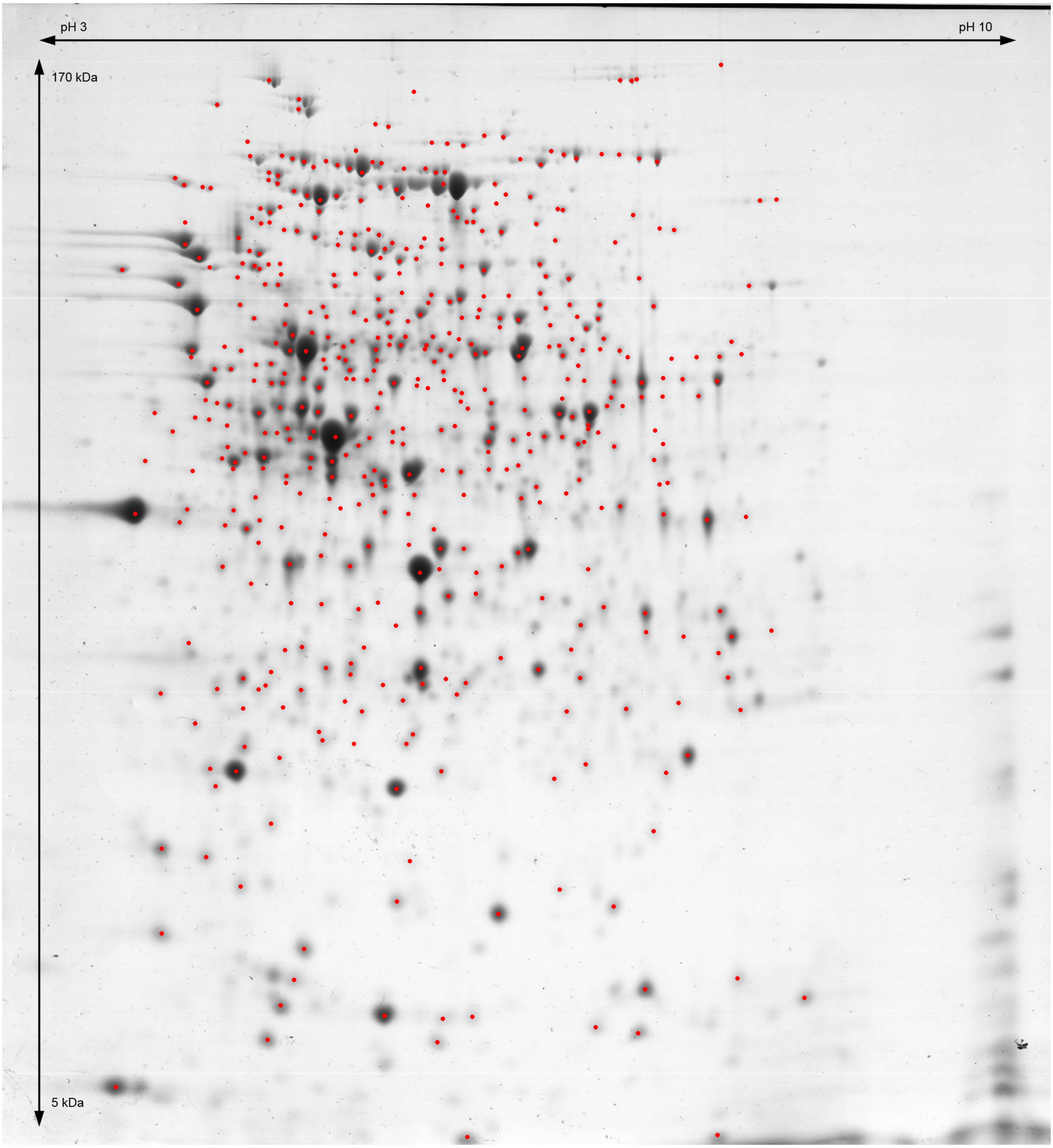

Figure S2 - 2D gel of *E. coli* BL21(DE3) growing in DNB medium at stationary phase

Click on the red spots to achieve detailed information of identified spots (proteins). Cultivation was carried out in shaker flask with baffles at 37 ºC and 200 rpm.

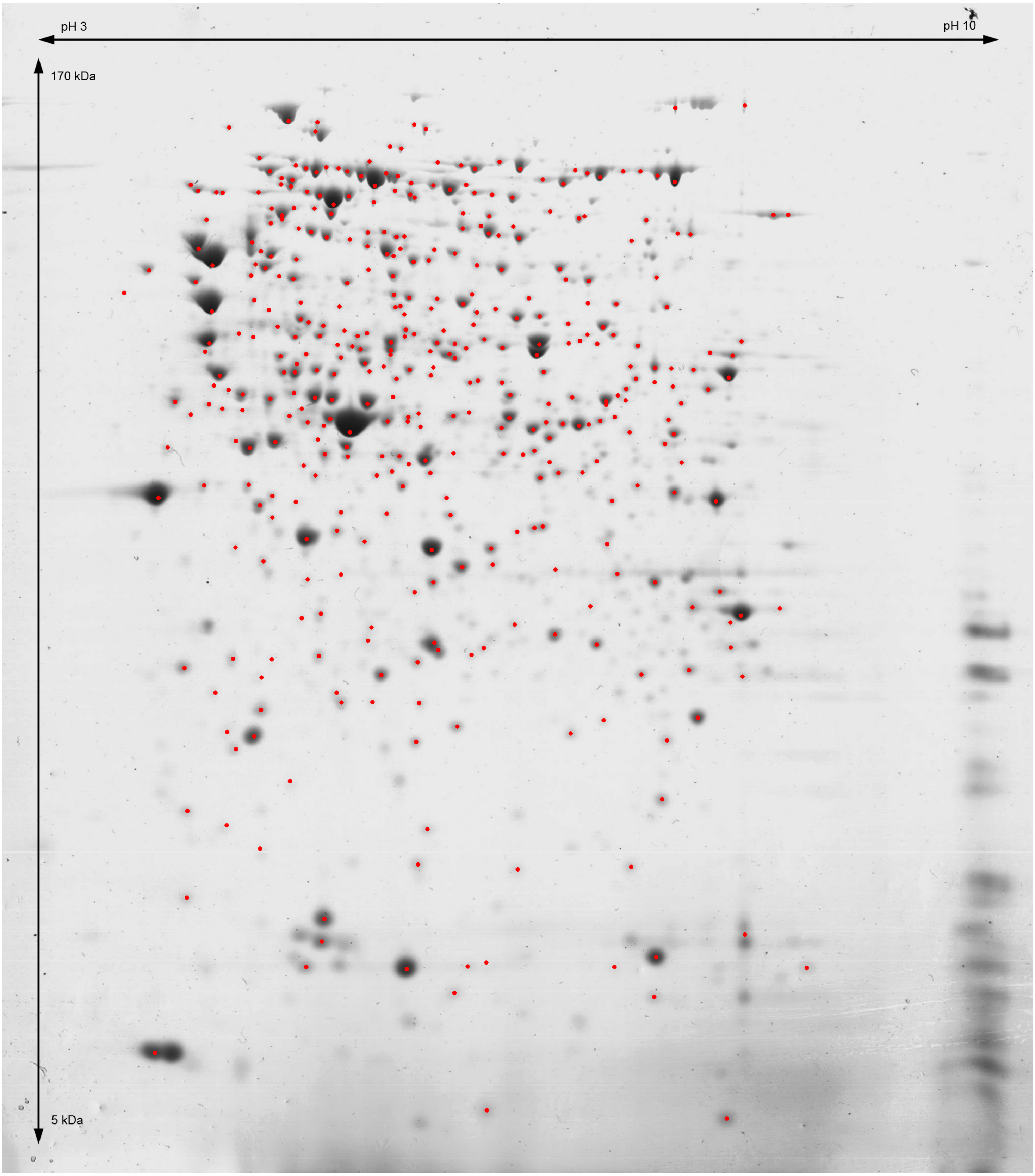

Figure S3 - 2D gel of *E. coli* BL21(DE3) growing in LB medium at exponential phase

Click on the red spots to achieve detailed information of identified spots (proteins). Cultivation was carried out in shaker flask with baffles at 37 ^{o}C and 200 rpm.

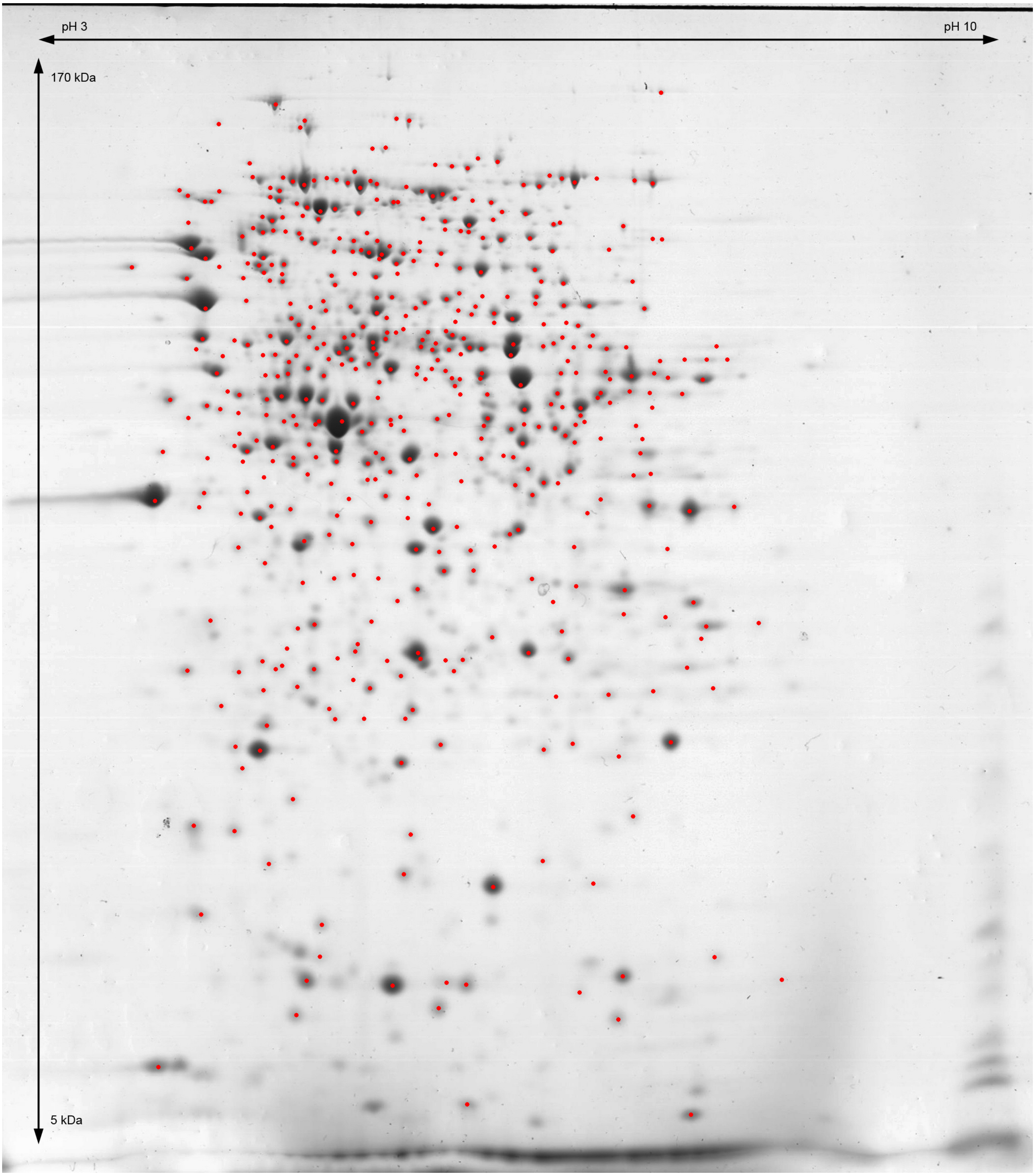

Figure S4 - 2D gel of *E. coli* BL21(DE3) growing in LB medium at stationary phase

Click on the red spots to achieve detailed information of identified spots (proteins). Cultivation was carried out in shaker flask with baffles at 37 ºC and 200 rpm.

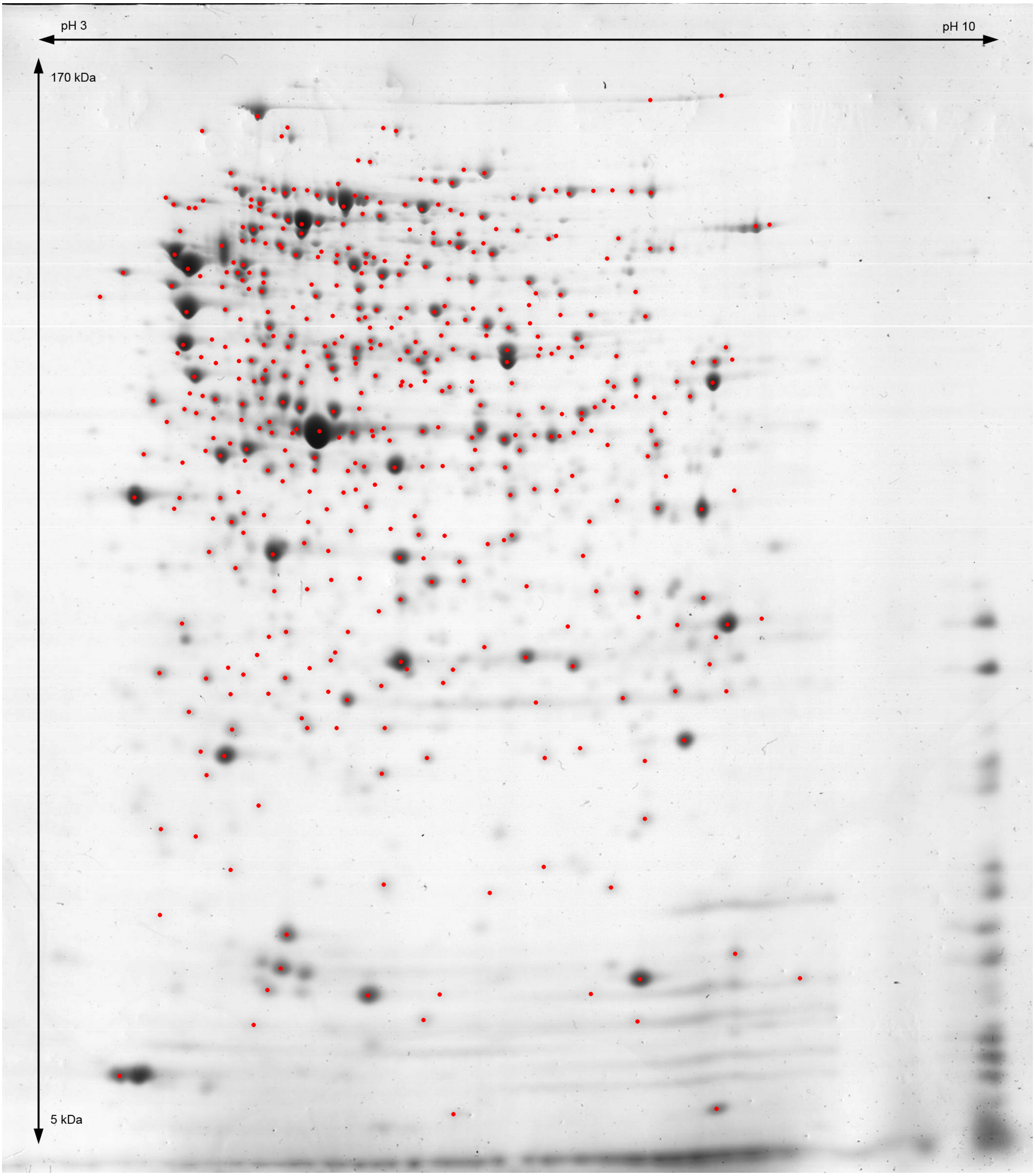

Figure S5 - 2D gel of *E. coli* BL21(DE3) growing in TB medium at exponential phase

Click on the red spots to achieve detailed information of identified spots (proteins). Cultivation was carried out in shaker flask with baffles at 37 °C and 200 rpm.

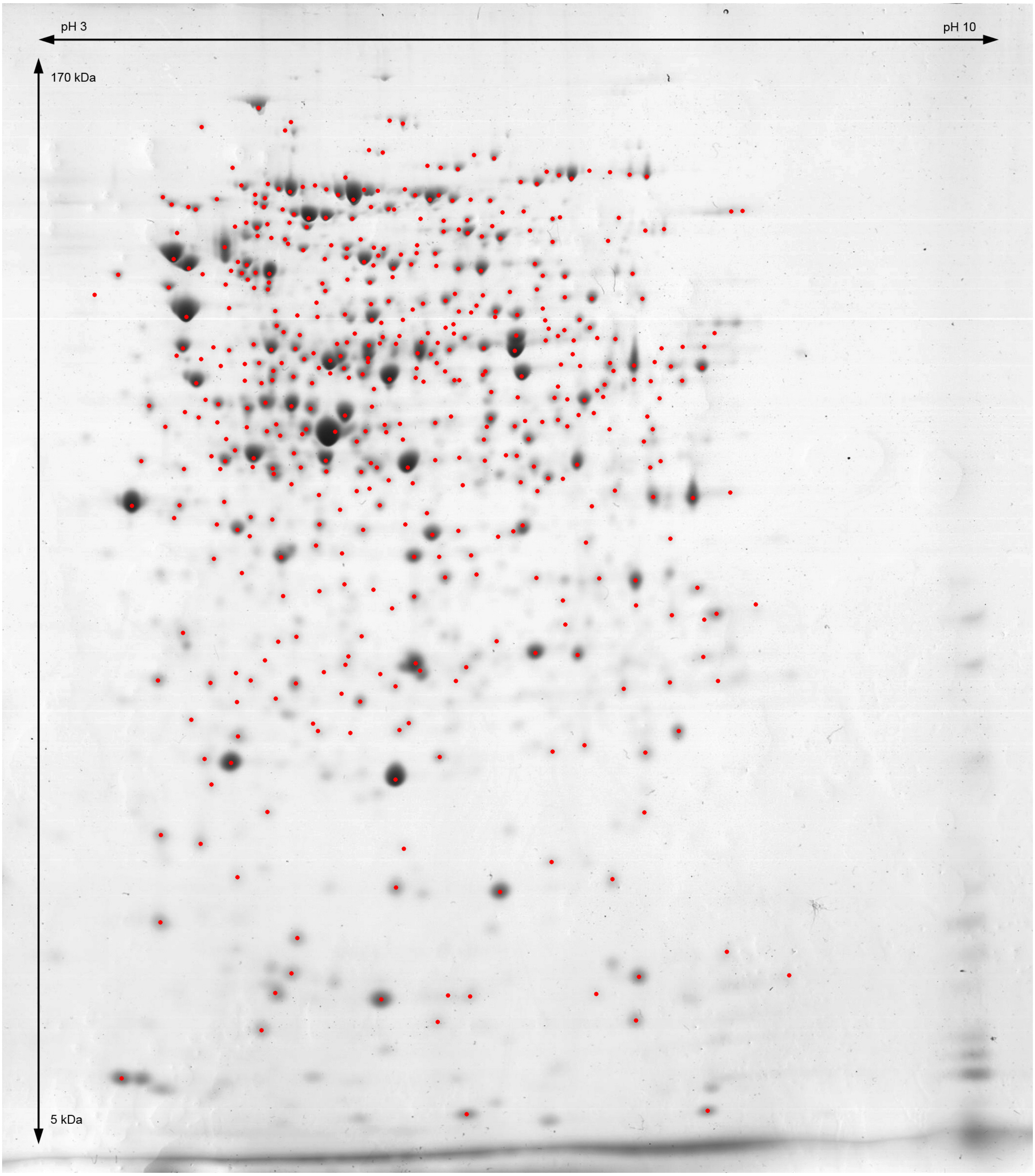

Figure S6 - 2D gel of *E. coli* BL21(DE3) growing in TB medium at stationary phase

Click on the red spots to achieve detailed information of identified spots (proteins). Cultivation was carried out in shaker flask with baffles at 37 ºC and 200 rpm.

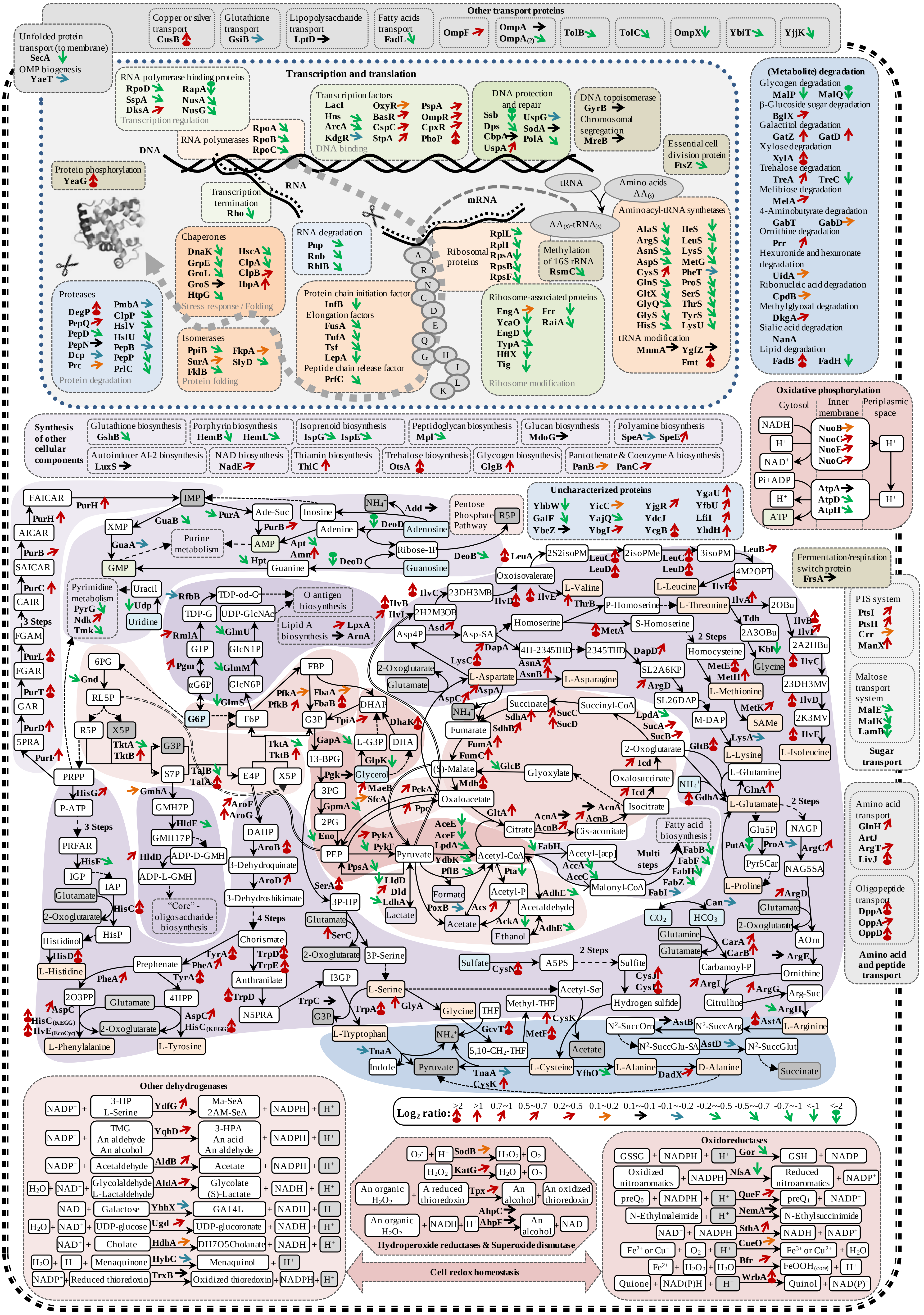

Figure S7 - Comparative proteome analysis of *E. coli*

Exponential phase in defined medium versus exponential phase in rich medium

Arrow indicates relative change of each protein (Log$_2$ ratio). Color code is shown in the figure. Value is given in Table S1. Print this page in A3 size.

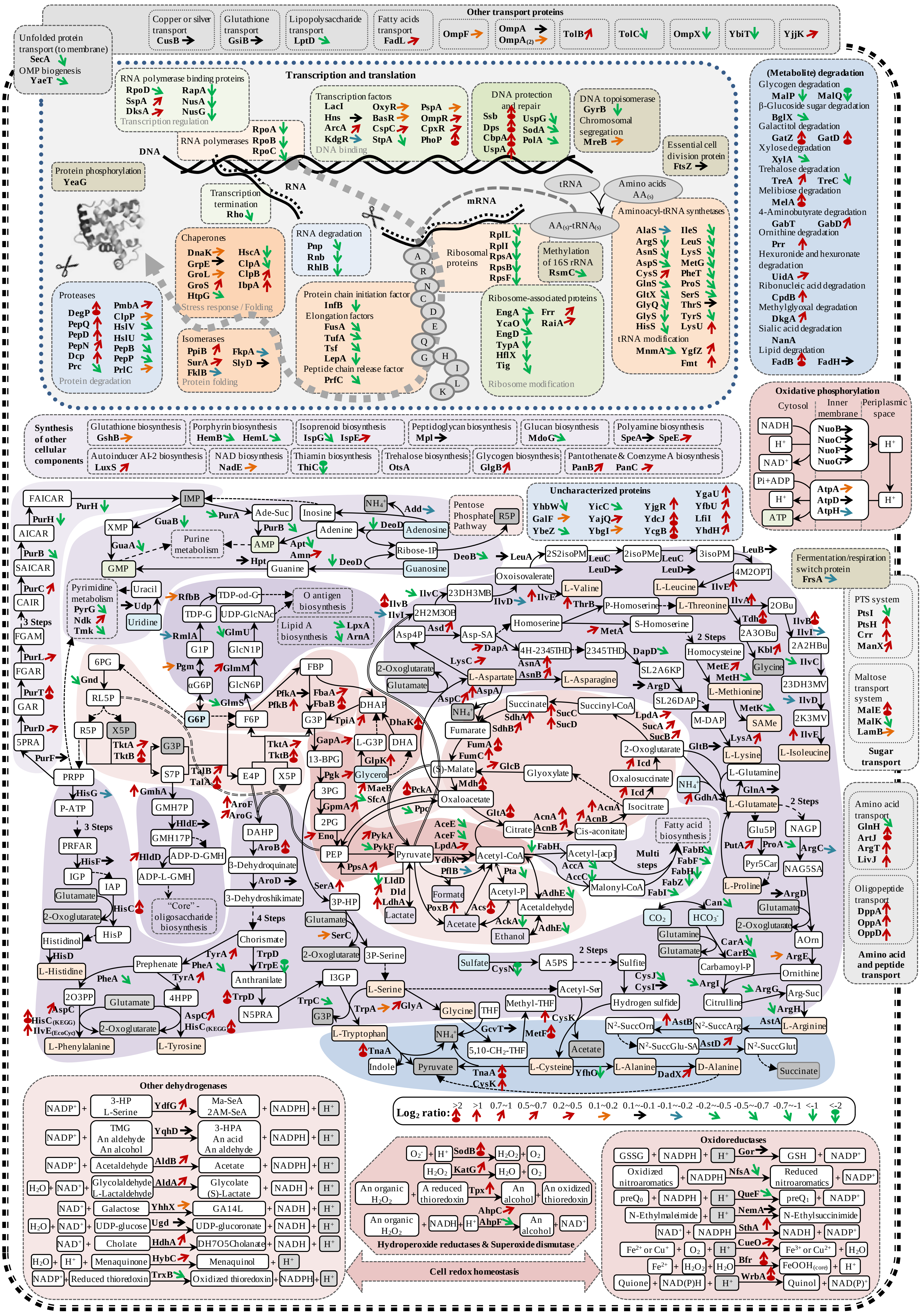

Figure S8 - Comparative proteome analysis of *E. coli*

Stationary phase versus exponential phase in rich medium

Arrow indicates relative change of each protein (Log_2 ratio). Color code is shown in the figure. Value is given in Table S1. Print this page in A3 size.

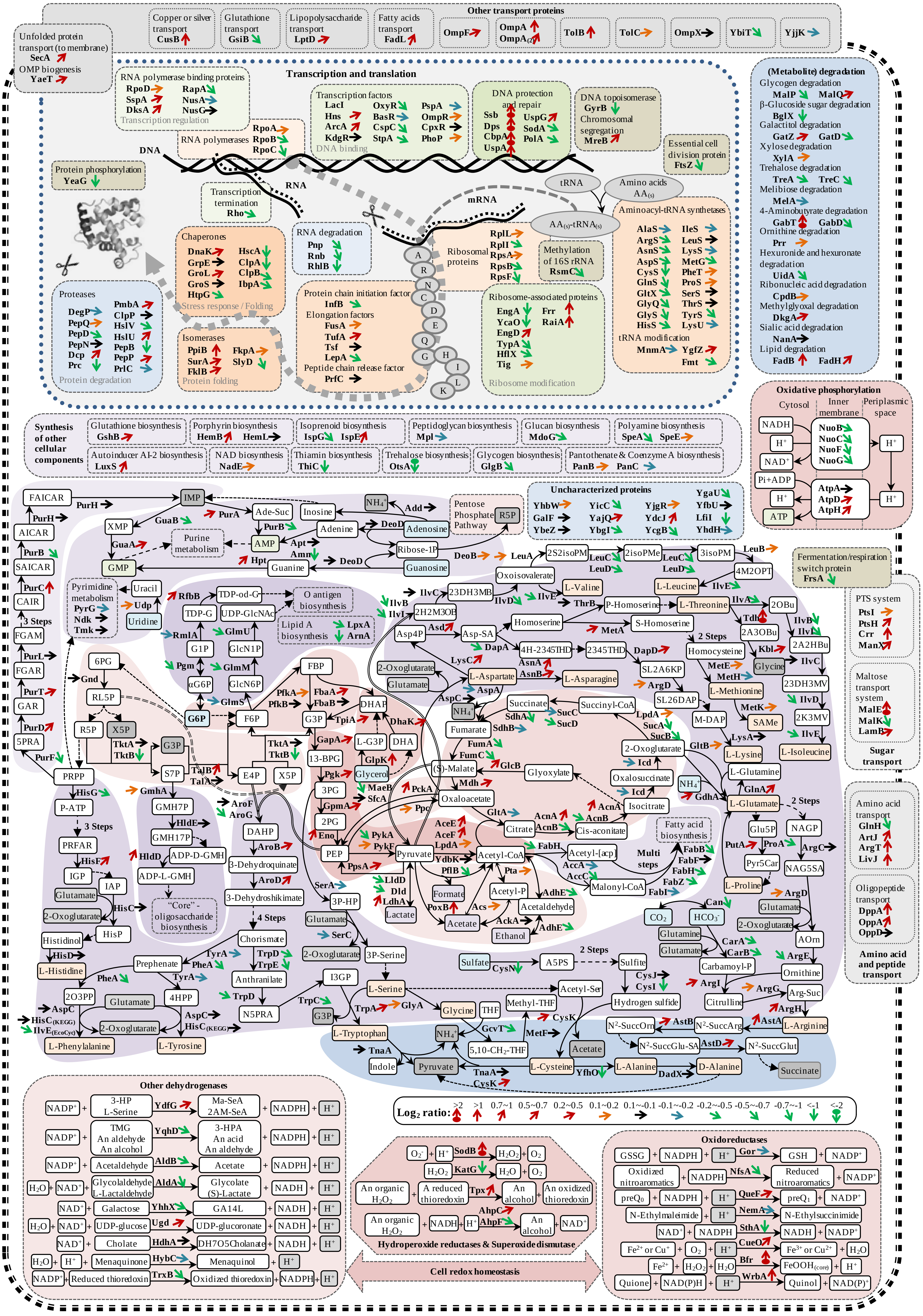

Figure S9 - Comparative proteome analysis of *E. coli*

Stationary phase versus exponential phase in defined medium

Arrow indicates relative change of each protein (Log$_2$ ratio). Color code is shown in the figure. Value is given in Table S1. Print this page in A3 size.

Figure captions for Figures S7, S8 and S9

A detailed direct comparison of pathway regulation at the proteomic level at different growth conditions is given in a pathway map using different color coded arrows for the representation of the comparative Log_2 ratios of the single protein abundances (code given in lower right of figure). Pathway map is drawn according to EcoCyc database (http://ecocyc.org/) [confirmed by KEGG database (http://www.genome.jp/kegg/)] based on current identified proteins. DNB medium was employed to indicate the glucose-supplemented defined mineral salt medium (defined medium). An average of single protein abundances from cells grown in LB and TB media was calculated to represent the single protein abundance in cells grown in rich medium. The corresponding values are given in the Table S1. The list of abbreviations is found at the end of the Figure S9.

Figure S**7**: Comparative proteome analysis of *E. coli* growing at exponential phase in defined medium versus exponential phase in rich medium.
Figure S**8**: Comparative proteome analysis of *E. coli* growing at stationary phase versus exponential phase in rich medium.
Figure S**9**: Comparative proteome analysis of *E. coli* growing at stationary phase versus exponential phase in defined medium.

Abbreviation used in Figures S7, S8 and S9

13-BPG	1,3-bisphospho-D-glycerate
2345THD	(*S*)-2,3,4,5-tetrahydrodipicolinate
23DH3MB	2,3-dihydroxy-3-methylbutanoate
23DH3MV	2,3-dihydroxy-3-methylvalerate
2A2HBu	2-aceto-2-hydroxy-butanoate
2A3OBu	2-amino-3-oxobutanoate
2AM-SeA	2-aminomalonate-semialdehyde
2H2M3OB	(*S*)-2-hydroxy-2-methyl-3-oxobutanoate (α-acetolactate)
2isoPMe	2-isopropylmaleate
2K3MV	2-keto-3-methyl-valerate
2OBu	2-oxobutanoate
2PG	2-phospho-D-glycerate
2S2isoPM	(2*S*)-2-isopropylmalate
3-HP	3-hydroxypropionate
3-HPA	3-hydroxypropionaldehyde
3isoPM	(2*R*,3*S*)-3-isopropylmalate
3PG	3-phospho-D-glycerate
3P-HP	3-phospho-hydroxypyruvate
3P-serine	3-phospho-L-serine
4H-2345THD	(2S,4S)-4-hydroxy-2,3,4,5-tetrahydrodipicolinate
4HPP	4-hydroxyphenylpyruvate

4M2OPT	4-methyl-2-oxopentanoate
5,10-CH_2-THF	5,10-methylenetetrahydrofolate
5PRA	5-phospho-β-D-ribosyl-amine
6PG	6-phospho-D-gluconate
A5PS	adenosine 5'-phosphosulfate
Acetyl-P	acetylphosphate
Acetyl-Ser	O-acetyl-L-serine
Ade-Suc	adenylo-succinate
ADP-D-GMH	ADP-D-glycero-β-D-manno-heptose
ADP-L-GMH	ADP-L-glycero-β-D-manno-heptose
AICAR	aminoimidazole carboxamide ribonucleotide
AOrn	N-acetyl-L-ornithine
Arg-Suc	L-arginino-succinate
Asp4P	L-aspartyl-4-phosphate
Asp-SA	L-aspartate-semialdehyde
CAIR	5-amino-1-(5-phospho-D-ribosyl)imidazole-4-carboxylate
Carbamoyl-P	carbamoyl-phosphate
DAHP	3-deoxy-D-arabino-heptulosonate-7-phosphate
DH7O5Cholanate	3α,12α-dihydroxy-7-oxo-5β-cholanate
DHA	dihydroxyacetone
DHAP	dihydroxyacetone phosphate
E4P	D-erythrose-4-phosphate
F6P	D-fructose-6-phosphate
FAICAR	phosphoribosyl-formamido-carboxamide
FBP	fructose-1,6-bisphosphate
FGAM	5-phosphoribosyl-*N*-formylglycineamidine
FGAR	5'-phosphoribosyl-*N*-formylglycineamide
G1P	α-D-glucose 1-phosphate
G3P	D-glyceraldehyde-3-phosphate
G6P	β-D-glucose-6-phosphate
GA14L	D-galactono-1,4-lactone
GAR	5-phospho-ribosyl-glycineamide
GlcN1P	D-glucosamine-1-phosphate
GlcN6P	D-glucosamine-6-phosphate
Glu5P	L-glutamate-5-phosphate
GMH17P	D-glycero-β-D-manno-heptose 1,7-bisphosphate
GMH7P	D-glycero-D-manno-heptose-7-phosphate
GSH	glutathione
GSSG	glutathione disulfide
HisP	L-histidinol-phosphate
I3GP	(1*S*,2*R*)-1-C-(indol-3-yl)glycerol 3-phosphate
IAP	imidazole acetol-phosphate

IGP	D-erythro-imidazole-glycerol-phosphate
IMP	inosine-5'-phosphate
L-G3P	*sn*-glycerol-3-phosphate
L-G3P	*sn*-glycerol-3-phosphate
Ma-SeA	malonate semialdehyde
M-DAP	*meso*-diaminopimelate
Methyl-THF	5-methyl-tetrahydrofolate
N^2-SuccArg	N^2-succinylarginine
N^2-SuccGlu-SA	N^2-succinylglutamic-semialdehyde
N^2-SuccGlut	N^2-succinylglutamate
N^2-SuccOrn	N^2-succinylornithine
N5PRA	N-(5'-phosphoribosyl)-anthranilate
NAG5SA	N-acetyl-L-glutamate 5-semialdehyde
NAGP	N-acetylglutamyl-phosphate
Nitroaromatics	nitroaromatic compound
OC1D5P	1-(o-carboxyphenylamino)-1'-deoxyribulose-5'-phosphate
2O3PP	2-oxo-3-phenylpropanoate
P-ATP	phosphoribosyl-ATP
PEP	phosphoenolpyruvate
P-Homoserine	O-phospho-L-homoserine
$preQ_0$	7-cyano-7-deazaguanine
$preQ_1$	7-aminomethyl-7-deazaguanine
PRFAR	phosphoribulosylformimino-AICAR-P
PRPP	5-phospho-α-D-ribose 1-diphosphate
Pyr5Car	(*S*)-1-pyrroline-5-carboxylate
R5P	D-ribose-5-phosphate
Ribose-1P	α-D-ribose-1-phosphate
RL5P	D-ribulose-5-phosphate
S7P	D-sedoheptulose-7-phosphate
SAICAR	5'-phosphoribosyl-4-(N-succinocarboxamide)-5-aminoimidazole
SAMe	S-adenosyl-L-methionine
S-Homoserine	O-succinyl-L-homoserine
SL26DAP	N-succinyl-L,L-2,6-diaminopimelate
SL2A6KP	N-succinyl-2-amino-6-ketopimelate
TDP-G	dTDP-α-D-glucose
TDP-od-G	dTDP-4-dehydro-6-deoxy-D-glucose
THF	tetrahydrofolate
TMG	1,3-propanediol
UDP-GlcNAc	UDP-α-N-acetyl-D-glucosamine
X5P	D-xylulose-5-phosphate
αG6P	α-D-glucose 6-phosphate

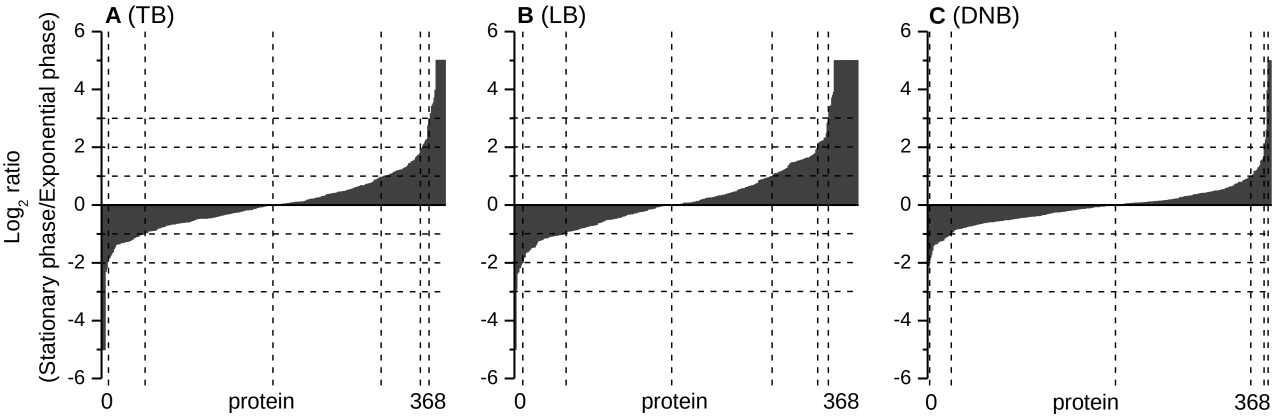

Figure S10 – Comparison of the stationary and exponential phase proteome of *E. coli* BL21 (DE3) in different media

The proteome comparisons are given as Log_2 ratios (stationary/ exponential phase) of single protein abundances of all identified proteins in rich medium (**A** and **B**) and in defined medium (**C**). Corresponding values of Log_2 (stationary/ exponential phase) values in TB medium (**A**), LB medium (B) and DNB medium (**C**) are also given in Table S1. Bar charts were generated from all identified individual proteins (x axis) and the corresponding values of Log_2 changes (y axis).

Table S1 - Quantitative data of individual proteins of *E. coli* BL21 (DE3) growing in defined and rich media

Name[1]	Protein name[1]	Uniprot ID[1]	Molecular mass[2] (Da)	Calculated pI value[2]	Mascot Score[2]	Mascot Expect Value[2]	Mascot Seq. Cover.[2]	Defined medium: DNB medium[7] exp. phase	Defined medium: DNB medium[7] stationary phase	Rich medium: TB medium[8] exp. phase	Rich medium: TB medium[8] stationary phase	Rich medium: LB medium[9] exp. phase	Rich medium: LB medium[9] stationary phase	Log_2 (DNB/TB) at exp. phase[4]	Log_2 (DNB/LB) at exp. phase[4]	**Log_2 (defined/rich)[4] at exp. phase[4]**	Log_2(stationary phase/exp. phase)[5, 6] in TB medium	Log_2(stationary phase/exp. phase)[5, 6] in LB medium	**Log_2(stationary phase/exp. phase)[5, 6] in rich media[5]**	**Log_2(stationary phase/exp. phase)[5, 6] in defined medium[6]**
								Relative Protein Mass (RPM)[3] - %												
**Central carbon metabolism (24 proteins):** Upper and lower glycolysis, pyruvate decarboxylation to acetyl CoA, glycerol metabolism and pentose phosphate pathway							**Total RPM:**	**7.05**	**9.29**	**10.07**	**14.06**	**9.96**	**9.34**	-0.50	-0.50	**-0.5**	0.50	-0.10	**0.2**	**0.4**
**Sub-group: Upper glycolysis (5 proteins)**							**Total RPM:**	**1.38**	**1.79**	**1.24**	**2.14**	**1.20**	**1.53**	0.20	0.20	**0.2**	0.80	0.30	**0.6**	**0.4**
PfkA	6-phosphofructokinase	P0A797	35162	5.47	105	2.7E-06	0.41	0.14	0.15	0.11	0.11	0.13	0.11	0.30	0.10	0.2	0.00	-0.20	-0.1	0.1
PfkB	6-phosphofructokinase isozyme 2	P06999	32664	5.25	145	7.2E-11	83%	0.040	0.037	0.026	0.05	0.018	0.06	0.60	1.20	0.9	1.00	1.80	1.4	-0.1
FbaA	fructose-bisphosphate aldolase class 2	P0AB71	39351	5.52	80	2.3E-04	25%	0.91	1.24	0.91	1.50	0.83	0.87	0.00	0.10	0.1	0.70	0.10	0.4	0.4
FbaB	fructose-bisphosphate aldolase class 1	P0A991	38313	6.25	59	2.8E-02	29%	0.031	0.031	0.00	0.06	0.00	0.12	>5	>5	>5	>5	>5	>5	0.0
TpiA	triosephosphate isomerase	P0A858	27126	5.64	77	4.4E-04	34%	0.26	0.32	0.18	0.42	0.23	0.37	0.50	0.20	0.4	1.20	0.70	1.0	0.3
**Sub-group: Lower glycolysis (7 proteins)**							**Total RPM:**	**2.47**	**3.35**	**3.20**	**4.56**	**3.31**	**3.63**	-0.40	-0.40	**-0.4**	0.50	0.10	**0.3**	**0.4**
GapA	glyceraldehyde-3-phosphate dehydrogenase A	P0A9B2	35681	6.61	58	3.8E-02	22%	0.70	0.92	0.93	1.28	1.01	1.28	-0.40	-0.50	-0.5	0.50	0.30	0.4	0.4
Pgk	phosphoglycerate kinase	P0A799	41264	5.08	231	6.6E-19	66%	0.75	1.00	0.77	1.07	0.72	0.76	0.00	0.10	0.1	0.50	0.10	0.3	0.4
GpmA	phosphoglyceromutase	P62709	28539	5.85	64	3.6E-02	37%	0.27	0.34	0.43	0.72	0.33	0.53	-0.70	-0.30	-0.5	0.70	0.70	0.7	0.4
Eno	enolase	A7ZQM2	45683	5.32	106	5.8E-07	41%	0.37	0.71	0.54	0.90	0.71	0.51	-0.50	-0.90	-0.7	0.80	-0.50	0.2	0.9
PykA	pyruvate kinase II	P21599	51553	6.24	115	7.3E-08	36%	0.12	0.08	0.09	0.14	0.08	0.10	0.40	0.60	0.5	0.70	0.30	0.5	-0.6
PykF	pyruvate kinase I	P0AD61	51039	5.77	70	2.1E-03	31%	0.20	0.22	0.36	0.29	0.32	0.23	-0.90	-0.70	-0.8	-0.30	-0.50	-0.4	0.1
PpsA	phosphoenolpyruvate synthase	P23538	87836	4.93	208	3.6E-17	32%	0.06	0.08	0.08	0.15	0.14	0.23	-0.30	-1.30	-0.8	1.00	0.60	0.8	0.4
**Sub-group: Pyruvate decarboxylation to acetyl CoA (4 proteins)**							**Total RPM:**	**2.01**	**2.80**	**4.16**	**4.77**	**3.91**	**2.16**	-1.0	-1.0	**-1.0**	0.2	-0.9	-0.3	**0.5**
AceE	pyruvate dehydrogenase E1 component	B3XKK6	99978	5.46	179	1.1E-13	28%	0.82	1.35	2.08	2.22	2.22	0.81	-1.30	-1.40	-1.4	0.10	-1.40	-0.7	0.7
AceF	pyruvate dehydrogenase, dihydrolipoyltransacetylase component E2	P06959	66112	5.09	156	2.1E-11	32%	0.34	0.53	0.98	0.85	0.66	0.31	-1.50	-1.00	-1.3	-0.20	-1.10	-0.7	0.6
LpdA[10]	dihydrolipoamide dehydrogenase	P0A9P2	50942	5.79	155	2.6E-11	43%	0.80	0.88	1.05	1.66	0.97	0.98	-0.40	-0.30	-0.4	0.70	0.00	0.4	0.1
YdbK	pyruvate flavodoxin/ferredoxin oxidoreductase domain protein	A7ZZU0	129871	5.51	76	2.0E-03	14%	0.044	0.041	0.046	0.041	0.06	0.06	0.00	-0.30	-0.2	-0.10	0.10	0.0	-0.1

Table S1 - Quantitative data of individual proteins of *E. coli* BL21 (DE3) growing in defined and rich media

Name[1]	Protein name[1]	Uniprot ID[1]	Molecular mass[2] (Da)	Calculated pI value[2]	Mascot Score[2]	Mascot Expect Value[2]	Mascot Seq. Cover.[2]	Defined medium: DNB medium[7] exp. phase	Defined medium: DNB medium[7] stationary phase	Rich medium: TB medium[8] exp. phase	Rich medium: TB medium[8] stationary phase	Rich medium: LB medium[9] exp. phase	Rich medium: LB medium[9] stationary phase	Log_2 (DNB /TB) at exp. phase[4]	Log_2 (DNB /LB) at exp. phase[4]	**Log_2 (defined/ rich)[4] at exp. phase[4]**	Log_2(stationary phase /exp. phase)[5, 6] in TB medium	Log_2(stationary phase /exp. phase)[5, 6] in LB medium	**Log_2(stationary phase /exp. phase)[5, 6] in rich media[5]**	**Log_2(stationary phase /exp. phase)[5, 6] in defined medium[6]**
								Relative Protein Mass (RPM)[3] - %												
**Sub-group: Glycerol metabolism (2 proteins)**							**Total RPM:**	**0.10**	**0.19**	**0.16**	**1.05**	**0.18**	**0.38**	-0.7	-0.8	**-0.8**	2.7	1.1	**1.9**	**1.0**
GlpK	glycerol kinase	C5A093	56480	5.36	129	2.9E-09	28%	0.08	0.16	0.14	0.98	0.18	0.33	-0.80	-1.20	-1.0	2.80	0.90	1.9	1.1
Dhak	dihydroxyacetone kinase subunit DhaK	A7ZZD5	38620	4.82	104	3.4E-06	44%	0.019	0.026	0.015	0.06	0.00	0.044	0.40	>5	>2.5	2.10	>5	>2.5	0.4
**Sub-group: Pentose phosphate pathway (PPP) (6 proteins)**							**Total RPM:**	**1.10**	**1.17**	**1.32**	**1.54**	**1.35**	**1.64**	-0.3	-0.3	**-0.3**	0.2	0.3	**0.3**	**0.1**
Gnd	6-phosphogluconate dehydrogenase, decarboxylating	Q59414	51563	5.06	133	1.1E-09	33%	0.26	0.26	0.30	0.23	0.37	0.19	-0.20	-0.50	-0.4	-0.40	-1.00	-0.7	0.0
TktA	transketolase 1	P27302	72451	5.43	75	7.2E-04	20%	0.49	0.47	0.64	0.64	0.60	0.75	-0.40	-0.30	-0.4	0.00	0.30	0.2	-0.1
TktB	transketolase 2	P33570	73225	5.86	190	2.3E-15	27%	0.034	0.016	0.016	0.042	0.011	0.12	1.00	1.60	1.3	1.40	3.50	2.5	-1.1
TalB	transaldolase B	P0A870	35368	5.11	136	5.7E-10	56%	0.19	0.30	0.28	0.47	0.31	0.41	-0.60	-0.70	-0.7	0.80	0.40	0.6	0.7
TalA	transaldolase A	P0A867	35865	5.89	211	1.8E-17	62%	0.05	0.050	0.00	0.049	0.00	0.10	>5	>5	>5	>5	>5	>5	-0.1
Eda	KHG/KDPG aldolase	P0A955	22441	5.57	108	3.6E-07	49%	0.06	0.08	0.09	0.10	0.06	0.07	-0.50	0.00	-0.3	0.20	0.20	0.2	0.3
**By-product metabolism (9 proteins)**							**Total RPM:**	**1.53**	**1.26**	**1.95**	**1.56**	**2.87**	**2.22**	-0.3	-0.9	**-0.6**	-0.3	-0.4	**-0.3**	**-0.3**
Acs	acetyl-coenzyme A synthetase	P27550	72447	5.5	224	9.1E-19	42%	0.10	0.12	0.08	0.13	0.043	0.60	0.40	1.20	0.8	0.80	3.80	2.3	0.2
PoxB	pyruvate dehydrogenase [ubiquinone]	Q47520	62542	5.86	160	2.3E-12	44%	0.019	0.048	0.019	0.05	0.025	0.11	0.00	-0.40	-0.2	1.50	2.20	1.9	1.3
Pta	phosphate acetyltransferase	P0A9M8	77466	5.28	72	1.3E-03	7%	0.14	0.15	0.37	0.20	0.33	0.14	-1.40	-1.20	-1.3	-0.80	-1.20	-1.0	0.1
AckA	acetate kinase	P0A6A3	43605	5.85	64	1.0E-02	18%	0.12	0.12	0.38	0.13	0.34	0.19	-1.60	-1.50	-1.6	-1.60	-0.90	-1.3	-0.1
AdhE	bifunctional acetaldehyde-CoA/alcohol dehydrogenase	P0A9Q8	96580	6.32	192	5.3E-15	26%	0.50	0.37	0.30	0.32	1.19	0.34	0.70	-1.20	-0.3	0.10	-1.80	-0.9	-0.5
PflB	formate acetyltransferase 1	P09373	85588	5.69	91	1.7E-05	20%	0.52	0.34	0.57	0.44	0.67	0.66	-0.10	-0.40	-0.3	-0.40	0.00	-0.2	-0.6
LldD	L-lactate dehydrogenase	A7ZTF9	42902	6.33	107	1.1E-05	22%	0.046	0.035	0.17	0.07	0.19	0.037	-1.90	-2.10	-2.0	-1.30	-2.40	-1.9	-0.4
Dld	D-lactate dehydrogenase	Q8FFW1	66546	6.39	106	2.1E-06	15%	0.045	0.032	0.025	0.06	0.037	0.042	0.80	0.30	0.6	1.20	0.20	0.7	-0.5
LdhA	D-lactate dehydrogenase	P52643	36854	5.29	115	7.2E-08	33%	0.031	0.05	0.032	0.15	0.045	0.10	0.00	-0.50	-0.3	2.20	1.20	1.7	0.7

Table S1 - Quantitative data of individual proteins of *E. coli* BL21 (DE3) growing in defined and rich media

Name[1]	Protein name[1]	Uniprot ID[1]	Molecular mass[2] (Da)	Calculated pI value[2]	Mascot Score[2]	Mascot Expect Value[2]	Mascot Seq. Cover.[2]	Defined medium: DNB medium[7]		Rich medium: TB medium[8]		Rich medium: LB medium[9]		Log_2 (DNB /TB)	Log_2 (DNB /LB)	**Log_2 (defined/ rich)[4]**	**Log_2(stationary phase /exp. phase)[5, 6]**			
								exp. phase	stationary phase	exp. phase	stationary phase	exp. phase	stationary phase				in TB medium	in LB medium	**in rich media[5]**	**in defined medium[6]**
								Relative Protein Mass (RPM)[3] - %						**at exp. phase[4]**						
**TCA cycle (15 proteins)**							**Total RPM:**	**7.98**	**6.77**	**4.56**	**9.99**	**5.28**	**10.44**	0.8	0.6	**0.7**	1.1	1.0	**1.1**	**-0.2**
GltA	citrate synthase	P0ABH7	48383	6.21	118	3.6E-08	42%	1.12	0.95	0.27	1.04	0.31	1.44	2.00	1.90	2.0	1.90	2.20	2.1	-0.2
AcnA	aconitate hydratase	P25516	98015	5.59	75	6.8E-04	17%	0.19	0.34	0.18	0.51	0.20	0.62	0.10	-0.10	0.0	1.50	1.60	1.6	0.9
AcnB	aconitate hydratase 2	B1LGR9	91078	5.22	213	4.2E-17	46%	0.88	0.67	0.51	1.07	0.70	1.16	0.80	0.30	0.6	1.10	0.70	0.9	-0.4
Icd	isocitrate dehydrogenase	Q7WV51	43029	5.18	197	1.7E-15	55%	1.06	0.96	0.71	1.13	0.68	1.44	0.60	0.70	0.7	0.70	1.10	0.9	-0.1
SucA	2-oxoglutarate dehydrogenase E1 component	P0AFG5	105566	6.04	99	1.0E-05	14%	0.60	0.31	0.32	0.72	0.61	0.62	0.90	0.00	0.5	1.20	0.00	0.6	-1.0
SucB	dihydrolipoamide succinyltransferase	A7ZXY7	44014	5.58	127	1.7E-08	38%	0.68	0.41	0.44	0.89	0.62	0.72	0.60	0.10	0.4	1.00	0.20	0.6	-0.7
SucC	succinyl-CoA synthetase subunit beta	P0A838	41652	5.37	256	2.1E-21	55%	0.59	0.54	0.29	0.78	0.30	0.71	1.00	1.00	1.0	1.40	1.20	1.3	-0.1
SucD	succinyl-CoA ligase [ADP-forming] subunit alpha	P0AGE9	30044	6.32	72	1.5E-03	29%	0.49	0.38	0.18	0.60	0.25	0.62	1.40	0.90	1.2	1.70	1.30	1.5	-0.4
SdhA	succinate dehydrogenase flavoprotein subunit	P0AC41	65008	5.85	59	2.7E-02	13%	0.64	0.36	0.24	0.59	0.27	0.64	1.40	1.20	1.3	1.30	1.20	1.3	-0.8
SdhB	succinate dehydrogenase and fumarate reductase iron-sulfur protein	Q8X9A8	27335	6.81	102	5.3E-06	34%	0.16	0.13	0.07	0.15	0.10	0.18	1.10	0.60	0.9	1.10	0.80	1.0	-0.2
FumA	fumarate hydratase (fumarase A), aerobic Class I	P0AC33	60774	6.11	116	2.1E-07	26%	0.07	0.039	0.029	0.08	0.022	0.12	1.20	1.60	1.4	1.50	2.50	2.0	-0.8
FumC	fumarate hydratase (fumarase C),aerobic Class II	P05042	50856	6.12	127	1.7E-08	29%	0.15	0.10	0.06	0.13	0.07	0.26	1.40	1.20	1.3	1.20	2.00	1.6	-0.6
Mdh	malate dehydrogenase	P61891	32488	5.61	79	1.0E-03	50%	0.53	0.64	0.14	0.61	0.10	0.77	2.00	2.40	2.2	2.20	2.90	2.6	0.3
LpdA[10]	dihydrolipoamide dehydrogenase	P0A9P2	50942	5.79	155	2.6E-11	43%	0.80	0.88	1.05	1.66	0.97	0.98	-0.40	-0.30	-0.4	0.70	0.00	0.4	0.1
GlcB	malate synthase G	P37330	80780	5.79	222	1.4E-18	37%	0.044	0.07	0.08	0.032	0.06	0.17	-0.80	-0.40	-0.6	-1.30	1.60	0.2	0.7
**Anaplerotic reactions (4 proteins)**							**Total RPM:**	**0.70**	**0.80**	**0.53**	**0.84**	**0.39**	**0.79**	0.4	0.8	**0.6**	0.7	1.0	**0.8**	**0.2**
Ppc	phosphoenolpyruvate carboxylase	C5A0C2	99456	5.52	66	5.2E-03	7%	0.41	0.45	0.25	0.17	0.24	0.17	0.70	0.80	0.8	-0.50	-0.50	-0.5	0.1
PckA	phosphoenolpyruvate carboxykinase [ATP]	C4ZUQ8	59891	5.46	219	2.9E-18	45%	0.14	0.24	0.14	0.54	0.06	0.46	0.00	1.10	0.6	1.90	2.80	2.4	0.8
MaeB	malate dehydrogenase (oxaloacetate-decarboxylating) (NADP(+))., phosphate	A8A2V3	82908	5.34	185	2.7E-14	46%	0.11	0.07	0.09	0.09	0.048	0.13	0.30	1.20	0.8	0.10	1.40	0.8	-0.7
SfcA	NAD-dependent malic enzyme	A7ZLS1	63481	5.19	88	3.2E-05	24%	0.045	0.041	0.05	0.040	0.032	0.029	-0.30	0.50	0.1	-0.40	-0.10	-0.3	-0.1

Table S1 - Quantitative data of individual proteins of *E. coli* BL21 (DE3) growing in defined and rich media

Name[1]	Protein name[1]	Uniprot ID[1]	Molecular mass[2] (Da)	Calculated pI value[2]	Mascot Score[2]	Mascot Expect Value[2]	Mascot Seq. Cover.[2]	Defined medium: DNB medium[7] exp. phase	Defined medium: DNB medium[7] stationary phase	Rich medium: TB medium[8] exp. phase	Rich medium: TB medium[8] stationary phase	Rich medium: LB medium[9] exp. phase	Rich medium: LB medium[9] stationary phase	Log$_2$ (DNB /TB)	Log$_2$ (DNB /LB)	**Log$_2$ (defined/ rich)[4]**	Log$_2$(stationary phase /exp. phase)[5, 6] in TB medium	in LB medium	**in rich media[5]**	**in defined medium[6]**
								Relative Protein Mass (RPM)[3] - %						**at exp. phase[4]**						
**Oxidative phosphorylation (7 proteins)**							**Total RPM:**	**2.68**	**2.52**	**2.31**	**2.69**	**2.87**	**2.42**	0.2	-0.1	**0.1**	0.2	-0.2	**0.0**	**-0.1**
AtpA	ATP synthase subunit alpha	A7ZTU6	55416	5.8	60	2.6E-02	26%	1.26	1.23	1.06	1.31	1.27	1.09	0.20	0.00	0.1	0.30	-0.20	0.1	0.0
AtpD	F0F1 ATP synthase subunit beta	P0ABB6	50351	4.9	113	4.2E-07	48%	0.50	0.64	0.63	0.66	0.69	0.57	-0.30	-0.50	-0.4	0.10	-0.30	-0.1	0.4
AtpH	F0F1 ATP synthase subunit delta	P0ABA5	19434	4.94	81	6.8E-04	41%	0.06	0.10	0.07	0.06	0.08	0.06	-0.10	-0.30	-0.2	-0.10	-0.30	-0.2	0.6
NuoF	NADH dehydrogenase I subunit F	Q8XCX1	49802	6.44	117	1.7E-07	33%	0.19	0.11	0.12	0.14	0.19	0.16	0.60	0.00	0.3	0.20	-0.20	0.0	-0.8
NuoG	NADH dehydrogenase subunit G	A8A2F2	101129	5.83	200	8.4E-16	35%	0.35	0.24	0.21	0.27	0.33	0.26	0.80	0.10	0.5	0.40	-0.40	0.0	-0.6
NuoC	bifunctional NADH:ubiquinone oxidoreductase subunit C/D	Q0TFG0	68451	5.98	64	3.1E-02	16%	0.22	0.13	0.15	0.16	0.19	0.18	0.50	0.20	0.4	0.10	0.00	0.1	-0.7
NuoB	NADH-quinone oxidoreductase subunit B	C4ZUD0	25325	5.58	74	8.7E-04	36%	0.10	0.08	0.08	0.08	0.11	0.09	0.40	-0.10	0.2	0.10	-0.30	-0.1	-0.4
**Amino acid biosynthesis and metabolism (68 proteins):** Amino acid biosynthesis and amino acid degradation							**Total RPM:**	**19.13**	**18.69**	**6.16**	**8.45**	**6.32**	**10.09**	1.6	1.6	**1.6**	0.5	0.7	**0.6**	**0.0**
**Sub-group: Amino acid biosynthesis (57 proteins)**							**Total RPM:**	**18.18**	**17.67**	**5.42**	**6.29**	**5.41**	**6.85**	1.7	1.7	**1.7**	0.2	0.3	**0.3**	**0.0**
AroD	3-dehydroquinate dehydratase	A8A0N9	27459	5.3	68	3.8E-03	44%	0.08	0.12	0.044	0.050	0.050	0.045	0.90	0.70	0.8	0.20	-0.20	0.0	0.6
AroB	3-dehydroquinate synthase	C4ZUP4	39141	5.72	91	1.7E-05	37%	0.025	0.028	0.018	0.027	0.00	0.028	0.40	>5	>2.5	0.60	>5	>2.5	0.2
AroF	phospho-2-dehydro-3-deoxyheptonate aldolase	Q3YYP3	39079	5.42	131	6.7E-09	35%	0.13	0.14	0.08	0.18	0.09	0.25	0.60	0.50	0.6	1.10	1.40	1.3	0.1
AroG	phospho-2-dehydro-3-deoxyheptonate aldolase, Phe-sensitive	P0AB91	38385	6.14	131	1.8E-09	53%	0.28	0.18	0.08	0.11	0.09	0.14	1.80	1.60	1.7	0.40	0.60	0.5	-0.6
TrpD	bifunctional glutamine amidotransferase/anthranilate	B3HP12	57176	6.26	157	1.7E-11	37%	0.14	0.11	0.00	0.00	0.00	0.027	>5	>5	>5	0.00	>5	-	-0.4
TrpE	component I of anthranilate synthase	P00895	58142	5.32	138	1.3E-09	30%	0.23	0.13	0.045	0.00	0.06	0.023	2.40	1.90	2.2	<-5	-1.50	<-2.5	-0.8
TrpC	anthranilate isomerase	C5W377	49774	5.43	191	6.7E-15	56%	0.12	0.10	0.12	0.11	0.12	0.07	0.00	0.00	0.0	-0.10	-0.70	-0.4	-0.3
TrpA	tryptophan synthase alpha chain	B7L492	28905	5.31	107	4.6E-07	38%	0.12	0.17	0.027	0.026	0.029	0.031	2.20	2.10	2.2	-0.10	0.10	0.0	0.4
PheA	P-protein	P0A9J8	43312	6.21	154	9.1E-12	40%	0.09	0.06	0.038	0.028	0.048	0.028	1.20	0.80	1.0	-0.40	-0.80	-0.6	-0.6
TyrA	T-protein	P07023	42187	5.68	165	7.2E-13	47%	0.12	0.11	0.029	0.045	0.031	0.06	2.00	1.90	2.0	0.60	1.00	0.8	-0.1
AspC	aspartate aminotransferase, PLP-dependent	P00509	43831	5.54	151	6.7E-11	46%	0.24	0.24	0.13	0.24	0.12	0.19	0.90	1.00	1.0	0.90	0.60	0.8	0.0

Table S1 - Quantitative data of individual proteins of *E. coli* BL21 (DE3) growing in defined and rich media

Name[1]	Protein name[1]	Uniprot ID[1]	Molecular mass[2] (Da)	Calculated pI value[2]	Mascot Score[2]	Mascot Expect Value[2]	Mascot Seq. Cover.[2]	Defined medium: DNB medium[7] exp. phase	Defined medium: DNB medium[7] stationary phase	Rich medium: TB medium[8] exp. phase	Rich medium: TB medium[8] stationary phase	Rich medium: LB medium[9] exp. phase	Rich medium: LB medium[9] stationary phase	Log_2 (DNB /TB) at exp. phase[4]	Log_2 (DNB /LB) at exp. phase[4]	**Log_2 (defined/ rich)[4] at exp. phase[4]**	Log_2(stationary phase /exp. phase)[5, 6] in TB medium	Log_2(stationary phase /exp. phase)[5, 6] in LB medium	**Log_2(stationary phase /exp. phase)[5, 6] in rich media[5]**	**Log_2(stationary phase /exp. phase)[5, 6] in defined medium[6]**
								Relative Protein Mass (RPM)[3] - %												
SerA	D-3-phosphoglycerate dehydrogenase	P0A9T2	44376	5.92	100	8.3E-06	32%	0.86	0.77	0.16	0.20	0.19	0.57	2.40	2.10	2.3	0.30	1.60	1.0	-0.2
SerC	phosphoserine aminotransferase	Q8FJB7	39974	5.36	169	1.1E-10	41%	0.49	0.43	0.18	0.16	0.17	0.23	1.40	1.50	1.5	-0.20	0.40	0.1	-0.2
GdhA	glutamate dehydrogenase, NADP-specific	P00370	48778	5.98	179	1.1E-13	52%	0.14	0.17	0.021	0.041	0.032	0.049	2.70	2.10	2.4	1.00	0.60	0.8	0.4
GlnA	glutamine synthetase	P0A9C7	52099	5.26	70	2.1E-03	15%	0.60	0.88	0.26	0.23	0.21	0.23	1.20	1.50	1.4	-0.20	0.10	-0.1	0.5
GltB	glutamate synthase [NADPH] large chain	P09831	164335	6.15	343	1.1E-30	38%	0.09	0.10	0.00	0.00	0.00	0.00	>5	>5	>5	0.00	0.00	0.0	0.1
HisG	ATP phosphoribosyltransferase	Q8X8T4	33615	5.59	104	9.1E-07	38%	0.15	0.11	0.11	0.08	0.09	0.09	0.40	0.80	0.6	-0.40	0.00	-0.2	-0.4
HisF	Imidazole glycerol phosphate synthase subunit hisF	C4ZSB4	28722	5.03	80	2.5E-04	38%	0.09	0.13	0.14	0.12	0.10	0.10	-0.60	-0.10	-0.4	-0.30	0.00	-0.2	0.5
HisC	histidinol-phosphate aminotransferase	B7NQG9	39907	5	144	9.1E-11	51%	0.09	0.09	0.035	0.047	0.00	0.033	1.30	>5	>2.5	0.40	>5	>2.5	0.1
HisD	histidinol dehydrogenase	A1ACN2	46939	5.13	125	2.7E-08	34%	0.041	0.041	0.030	0.026	0.00	0.014	0.40	>5	>2.5	-0.20	>5	-	0.0
GlyA	serine hydroxymethyltransferase	C4ZXC6	45459	6.03	103	1.1E-06	31%	0.80	0.82	0.33	0.51	0.38	0.56	1.30	1.10	1.2	0.60	0.60	0.6	0.0
CysK	cysteine synthase A	P0ABK5	34525	5.83	176	5.7E-14	60%	0.67	0.85	0.25	0.56	0.12	0.38	1.40	2.50	2.0	1.10	1.60	1.4	0.4
CysN	sulfate adenylyltransferase, subunit 1	P23845	52640	4.98	56	5.6E-02	19%	0.10	0.043	0.023	0.00	0.020	0.00	2.10	2.30	2.2	<-5	<-5	<-5	-1.2
CysJ	sulfite reductase subunit alpha	A8A3P5	66415	4.92	161	6.6E-12	33%	0.07	0.07	0.015	0.017	0.035	0.013	2.30	1.10	1.7	0.20	-1.50	-0.7	0.0
CysI	sulfite reductase subunit beta	Q8X7U2	64262	7.27	79	9.9E-04	21%	0.07	0.031	0.00	0.00	0.00	0.00	>5	>5	>5	0.00	0.00	0.0	-1.3
LysC	aspartokinase III	P08660	48787	5.03	237	1.7E-19	62%	0.05	0.08	0.016	0.022	0.00	0.00	1.80	>5	>2.5	0.50	0.00	0.3	0.6
Asd	aspartate-semialdehyde dehydrogenase	P0A9Q9	40221	5.37	128	3.6E-09	53%	0.38	0.58	0.34	0.44	0.17	0.36	0.20	1.20	0.7	0.40	1.10	0.8	0.6
DapA	dihydrodipicolinate synthase	A7ZPS4	31549	5.98	119	2.9E-08	42%	0.15	0.10	0.09	0.10	0.10	0.14	0.70	0.50	0.6	0.20	0.40	0.3	-0.5
DapD	2,3,4,5-tetrahydropyridine-2-carboxylate N-succinyltransferase	P0A9D8	30045	5.56	169	1.0E-12	63%	0.31	0.35	0.21	0.16	0.17	0.16	0.60	0.90	0.8	-0.40	-0.10	-0.3	0.2
LysA	diaminopimelate decarboxylase	P00861	46377	5.63	106	1.3E-05	45%	0.11	0.11	0.13	0.28	0.13	0.16	-0.20	-0.20	-0.2	1.10	0.30	0.7	-0.1
ThrB	homoserine kinase	A7ZH92	34101	5.44	90	2.3E-05	34%	0.08	0.07	0.033	0.07	0.017	0.05	1.20	2.20	1.7	1.00	1.70	1.4	-0.1
MetA	homoserine O-succinyltransferase	C5A0V0	35819	5.06	106	5.7E-07	35%	0.06	0.07	0.025	0.00	0.00	0.048	1.20	>5	>2.5	<-5	>5	-	0.4
MetH	methionine synthase	B3XC96	136639	4.97	291	6.7E-25	38%	0.07	0.06	0.028	0.030	0.030	0.015	1.40	1.30	1.4	0.10	-1.00	-0.5	-0.2
MetE	5-methyltetrahydropteroyltriglutamate-homocysteine S-methyltransferase	B3XGD6	85074	5.61	107	1.7E-06	31%	3.60	3.74	0.08	0.22	0.13	0.10	5.50	4.80	5.2	1.40	-0.30	0.6	0.1

Table S1 - Quantitative data of individual proteins of *E. coli* BL21 (DE3) growing in defined and rich media

Name[1]	Protein name[1]	Uniprot ID[1]	Molecular mass[2] (Da)	Calculated pI value[2]	Mascot Score[2]	Mascot Expect Value[2]	Mascot Seq. Cover.[2]	Defined medium: DNB medium[7] exp. phase	Defined medium: DNB medium[7] stationary phase	Rich medium: TB medium[8] exp. phase	Rich medium: TB medium[8] stationary phase	Rich medium: LB medium[9] exp. phase	Rich medium: LB medium[9] stationary phase	Log$_2$ (DNB /TB) at exp. phase[4]	Log$_2$ (DNB /LB) at exp. phase[4]	**Log$_2$ (defined/ rich)[4]** at exp. phase[4]	Log$_2$(stationary phase /exp. phase)[5, 6] in TB medium	Log$_2$(stationary phase /exp. phase)[5, 6] in LB medium	**Log$_2$(stationary phase /exp. phase)[5, 6] in rich media[5]**	**Log$_2$(stationary phase /exp. phase)[5, 6] in defined medium[6]**
								Relative Protein Mass (RPM)[3] - %												
IlvA	threonine dehydratase biosynthetic	P04968	56559	5.57	213	2.7E-16	48%	0.27	0.19	0.08	0.13	0.05	0.16	1.70	2.30	2.0	0.60	1.50	1.1	-0.5
IlvB	acetolactate synthase I, large subunit	P08142	60915	5.3	85	2.4E-04	24%	0.10	0.050	0.00	0.044	0.011	0.06	>5	3.20	>2.5	>5	2.30	>2.5	-1.0
IlvC	ketol-acid reductoisomerase	C4ZZ44	54376	5.2	93	1.1E-05	38%	2.83	2.84	0.17	0.19	0.14	0.08	4.00	4.30	4.2	0.10	-0.80	-0.4	0.0
IlvD	dihydroxyacid dehydratase	P05791	66174	5.59	235	2.6E-19	41%	0.58	0.36	0.09	0.07	0.06	0.06	2.70	3.30	3.0	-0.40	0.00	-0.2	-0.7
IlvE	branched-chain-amino-acid aminotransferase	P0AB80	34112	5.54	108	1.3E-06	44%	0.54	0.41	0.06	0.14	0.046	0.20	3.10	3.60	3.4	1.20	2.20	1.7	-0.4
IlvI	acetolactate synthase isozyme 3 large subunit	P00893	63286	5.88	81	4.3E-03	25%	0.07	0.043	0.045	0.028	0.048	0.06	0.60	0.50	0.6	-0.70	0.30	-0.2	-0.6
LeuA	2-isopropylmalate synthase	A7ZHG6	57590	5.47	156	5.8E-12	46%	0.09	0.09	0.00	0.00	0.00	0.00	>5	>5	>5	0.00	0.00	0.0	0.1
LeuB	3-isopropylmalate dehydrogenase	P30125	39834	5.14	72	5.6E-03	27%	0.15	0.16	0.16	0.13	0.10	0.11	0.00	0.60	0.3	-0.30	0.10	-0.1	0.1
LeuC	isopropylmalate isomerase large subunit	A7ZW23	50377	5.9	162	5.3E-12	42%	0.20	0.13	0.00	0.00	0.00	0.020	>5	>5	>5	0.00	>5	-	-0.6
LeuD	3-isopropylmalate isomerase subunit	P30126	22587	5.16	128	1.3E-08	56%	0.08	0.06	0.00	0.00	0.00	0.00	>5	>5	>5	0.00	0.00	0.0	-0.5
AsnA	aspartate-ammonia ligase	B1LL71	36770	5.55	89	1.1E-04	33%	0.06	0.11	0.024	0.07	0.043	0.14	1.40	0.50	1.0	1.50	1.70	1.6	0.9
AsnB	asparagine synthase	B3ISY7	63075	5.55	84	3.4E-04	27%	0.10	0.12	0.037	0.05	0.043	0.08	1.40	1.20	1.3	0.50	0.90	0.7	0.3
CarA	carbamoyl phosphate synthase small subunit	B7NHD6	41677	5.91	107	1.7E-06	39%	0.22	0.16	0.08	0.040	0.17	0.10	1.50	0.40	1.0	-1.00	-0.70	-0.9	-0.5
CarB	carbamoyl phosphate synthase large subunit	B7MNN9	118633	5.22	103	4.2E-06	14%	0.51	0.36	0.16	0.12	0.33	0.19	1.70	0.60	1.2	-0.40	-0.80	-0.6	-0.5
ArgC	N-acetyl-gamma-glutamyl-phosphate reductase	B1LNR8	36328	5.58	105	2.7E-06	33%	0.08	0.08	0.041	0.035	0.05	0.046	1.00	0.50	0.8	-0.20	-0.20	-0.2	0.0
ArgD	acetylornithine/succinyldiaminopimelate aminotransferase	P18335	44081	5.79	141	6.6E-10	60%	0.30	0.33	0.19	0.19	0.19	0.17	0.70	0.70	0.7	0.00	-0.10	-0.1	0.1
ArgE	acetylornithine deacetylase	C5A0C3	42777	5.54	99	3.0E-06	37%	0.06	0.042	0.07	0.05	0.05	0.08	-0.20	0.30	0.1	-0.40	0.70	0.2	-0.6
ArgI	ornithine carbamoyltransferase chain I	P04391	37112	5.46	93	1.2E-05	35%	0.05	0.07	0.034	0.021	0.041	0.040	0.70	0.40	0.6	-0.70	0.00	-0.4	0.4
ArgG	argininosuccinate synthase	B1XGY3	50038	5.23	143	2.6E-09	35%	0.27	0.30	0.13	0.09	0.18	0.13	1.10	0.60	0.9	-0.50	-0.50	-0.5	0.1
ArgH	argininosuccinate lyase	P11447	50686	5.11	128	1.3E-08	38%	0.13	0.17	0.16	0.10	0.20	0.10	-0.30	-0.60	-0.5	-0.60	-0.90	-0.8	0.5
AspA	aspartate ammonia-lyase	P0AC38	52950	5.19	135	7.2E-10	29%	0.36	0.33	0.24	0.52	0.29	0.54	0.60	0.30	0.5	1.10	0.90	1.0	-0.1
ProA	gamma-glutamyl phosphate reductase	B3XF57	45031	5.42	205	2.7E-16	52%	0.05	0.038	0.06	0.039	0.06	0.047	-0.20	-0.20	-0.2	-0.60	-0.30	-0.5	-0.5
IscS (Yfho)	cysteine desulfurase	C4ZXA5	45232	5.94	127	4.6E-09	57%	0.41	0.12	0.46	0.14	0.58	0.25	-0.20	-0.50	-0.4	-1.70	-1.20	-1.5	-1.7

Table S1 - Quantitative data of individual proteins of *E. coli* BL21 (DE3) growing in defined and rich media

Name[1]	Protein name[1]	Uniprot ID[1]	Molecular mass[2] (Da)	Calculated pI value[2]	Mascot Score[2]	Mascot Expect Value[2]	Mascot Seq. Cover.[2]	Defined medium: DNB medium[7] exp. phase	Defined medium: DNB medium[7] stationary phase	Rich medium: TB medium[8] exp. phase	Rich medium: TB medium[8] stationary phase	Rich medium: LB medium[9] exp. phase	Rich medium: LB medium[9] stationary phase	Log_2 (DNB /TB) at exp. phase[4]	Log_2 (DNB /LB) at exp. phase[4]	**Log_2 (defined/ rich)[4]** at exp. phase[4]	Log_2(stationary phase /exp. phase)[5, 6] in TB medium	Log_2(stationary phase /exp. phase)[5, 6] in LB medium	**in rich media[5]**	**in defined medium[6]**
								Relative Protein Mass (RPM)[3] - %												
**Sub-group: Amino acid degradation (11 proteins)**							**Total RPM:**	**0.95**	**1.02**	**0.74**	**2.17**	**0.91**	**3.24**	0.4	0.1	**0.2**	1.6	1.8	**1.7**	**0.1**
DadX	alanine racemase, catabolic	P29012	39048	6.56	60	2.6E-02	31%	0.045	0.045	0.031	0.037	0.033	0.05	0.60	0.40	0.5	0.30	0.70	0.5	0.0
TnaA	tryptophanase	B1LL35	53107	5.88	97	4.8E-06	20%	0.10	0.10	0.10	1.09	0.13	1.94	0.00	-0.30	-0.2	3.40	3.90	3.7	-0.1
MetF	5,10-methylenetetrahydrofolate reductase	P0AEZ1	33253	6	165	7.3E-13	52%	0.12	0.11	0.00	0.041	0.00	0.031	>5	>5	>5	>5	>5	>5	-0.1
MetK	chain A, S-adenosylmethionine synthetase	P0A817	42022	5.1	155	2.6E-11	61%	0.37	0.38	0.25	0.22	0.26	0.17	0.60	0.50	0.6	-0.20	-0.60	-0.4	0.1
GcvT	glycine cleavage system T protein	B2ND95	40251	5.36	88	1.3E-04	34%	0.10	0.08	0.00	0.00	0.00	0.00	>5	>5	>5	0.00	0.00	0.0	-0.4
AstA	arginine N-succinyltransferase	C4ZZA3	38831	6.02	172	1.4E-13	58%	0.026	0.048	0.00	0.00	0.00	0.10	>5	>5	>5	0.00	>5	-	0.9
AstB	N-succinylarginine dihydrolase	C4ZZA1	49439	5.74	116	5.7E-08	41%	0.027	0.034	0.026	0.035	0.027	0.09	0.10	0.00	0.1	0.40	1.70	1.1	0.3
AstD	N-succinylglutamate 5-semialdehyde dehydrogenase	C4ZZA2	53278	5.69	85	6.8E-05	29%	0.046	0.06	0.05	0.033	0.048	0.12	-0.20	-0.10	-0.2	-0.70	1.30	0.3	0.4
Tdh	L-threonine 3-dehydrogenase	C4ZXK8	37557	5.94	126	5.7E-09	56%	0.00	0.034	0.00	0.18	0.11	0.17	0.00	<-5	-	>5	0.60	>2.5	>5
Kbl	2-amino-3-ketobutyrate coenzyme A ligase	P0AB77	43432	5.64	127	4.6E-09	47%	0.12	0.13	0.21	0.42	0.24	0.46	-0.80	-1.00	-0.9	1.00	0.90	1.0	0.1
PutA	bifunctional protein PutA	P09546	144467	5.69	148	3.6E-11	13%	0.005	0.006	0.07	0.10	0.06	0.10	-3.90	-3.70	-3.8	0.50	0.70	0.6	0.4
**IMP biosynthesis (for nucleotide) (7 proteins)**							**Total RPM:**	**1.28**	**1.39**	**0.43**	**0.47**	**0.39**	**0.61**	1.6	1.7	**1.6**	0.1	0.6	**0.4**	**0.1**
PurF	amidophosphoribosyltransferase	P0AG16	56852	5.33	82	4.7E-04	22%	0.06	0.032	0.026	0.023	0.016	0.017	1.30	2.00	1.7	-0.20	0.10	-0.1	-1.0
PurD	phosphoribosylamine-glycine ligase	A7ZUM2	46326	4.89	80	8.9E-04	36%	0.16	0.24	0.08	0.08	0.05	0.07	1.10	1.60	1.4	0.10	0.50	0.3	0.6
PurT	phosphoribosylglycinamide formyltransferase 2	C4ZZK6	42692	5.48	178	3.6E-14	46%	0.10	0.11	0.05	0.22	0.00	0.21	0.90	>5	>2.5	2.10	>5	>2.5	0.2
PurL	phosphoribosylformyl-glycineamide synthetase	B3Y153	142036	5.23	149	1.0E-10	24%	0.37	0.37	0.023	0.015	0.06	0.11	4.00	2.70	3.4	-0.60	0.90	0.2	0.0
PurC	phosphoribosylaminoimidazole-succinocarboxamide synthase	A7ZPS1	27149	5.07	136	5.7E-10	51%	0.09	0.21	0.023	0.034	0.037	0.07	2.00	1.30	1.7	0.60	0.90	0.8	1.2
PurB	adenylosuccinate lyase	Q8X737	51652	5.68	173	4.2E-13	45%	0.17	0.12	0.12	0.08	0.14	0.08	0.60	0.30	0.5	-0.60	-0.80	-0.7	-0.6
PurH	bifunctional phosphoribosylaminoimidazolecarboxamide	Q1R5X1	57747	5.53	81	6.4E-04	19%	0.32	0.31	0.11	0.024	0.09	0.05	1.50	1.80	1.7	-2.20	-0.70	-1.5	0.0

Table S1 - Quantitative data of individual proteins of *E. coli* BL21 (DE3) growing in defined and rich media

Name[1]	Protein name[1]	Uniprot ID[1]	Molecular mass[2] (Da)	Calculated pI value[2]	Mascot Score[2]	Mascot Expect Value[2]	Mascot Seq. Cover.[2]	Defined medium: DNB medium[7] exp. phase	Defined medium: DNB medium[7] stationary phase	Rich medium: TB medium[8] exp. phase	Rich medium: TB medium[8] stationary phase	Rich medium: LB medium[9] exp. phase	Rich medium: LB medium[9] stationary phase	Log_2 (DNB /TB) at exp. phase[4]	Log_2 (DNB /LB) at exp. phase[4]	**Log_2 (defined/ rich)[4] at exp. phase[4]**	Log_2(stationary phase /exp. phase)[5, 6] in TB medium	Log_2(stationary phase /exp. phase)[5, 6] in LB medium	**Log_2(stationary phase /exp. phase)[5, 6] in rich media[5]**	**Log_2(stationary phase /exp. phase)[5, 6] in defined medium[6]**
								Relative Protein Mass (RPM)[3] - %												
**Nucleotide biosynthesis (start from IMP) (13 proteins)**							**Total RPM:**	**1.78**	**1.77**	**2.92**	**2.01**	**2.59**	**1.92**	-0.7	-0.5	**-0.6**	-0.5	-0.4	**-0.5**	**0.0**
PurA	adenylosuccinate synthetase	A7ZV47	47543	5.31	172	1.4E-13	51%	0.35	0.40	0.44	0.37	0.48	0.30	-0.30	-0.50	-0.4	-0.30	-0.70	-0.5	0.2
GuaA	GMP synthetase (glutamine aminotransferase)	P04079	59041	5.24	178	1.3E-13	41%	0.16	0.19	0.19	0.09	0.16	0.08	-0.30	-0.10	-0.2	-1.00	-1.00	-1.0	0.3
GuaB	inositol-5-monophosphate dehydrogenase	P0ADG8	52275	6.02	94	3.7E-05	35%	0.18	0.13	0.25	0.12	0.26	0.08	-0.50	-0.50	-0.5	-1.10	-1.70	-1.4	-0.4
Add	adenosine deaminase	C4ZY85	36603	5.36	72	1.6E-03	40%	0.038	0.037	0.046	0.030	0.034	0.035	-0.30	0.20	-0.1	-0.60	0.10	-0.3	-0.1
DeoB	phosphopentomutase	C4ZT65	44684	5.11	158	3.6E-12	44%	0.08	0.10	0.14	0.10	0.07	0.06	-0.70	0.20	-0.3	-0.40	-0.20	-0.3	0.2
DeoD	chain A, purine nucleoside phosphorylase	P0ABP8	26030	5.42	130	8.3E-09	62%	0.07	0.07	0.37	0.19	0.33	0.15	-2.40	-2.20	-2.3	-1.00	-1.10	-1.1	0.0
Hpt	hypoxanthine phosphoribosyltransferase	B1LGS6	20315	5.09	76	2.0E-03	56%	0.14	0.20	0.24	0.22	0.23	0.24	-0.70	-0.60	-0.7	-0.10	0.10	0.0	0.5
Apt	adenine phosphoribosyltransferase	P69503	19847	5.26	71	6.5E-03	52%	0.09	0.09	0.13	0.07	0.14	0.08	-0.50	-0.70	-0.6	-0.90	-0.90	-0.9	0.0
Udp	uridine phosphorylase	Q8X8L3	27304	5.71	81	6.8E-04	54%	0.10	0.11	0.34	0.31	0.22	0.21	-1.70	-1.10	-1.4	-0.10	-0.10	-0.1	0.1
PyrG	CTP synthase	A7ZQM3	60792	5.63	74	1.0E-03	15%	0.25	0.21	0.52	0.33	0.45	0.33	-1.00	-0.80	-0.9	-0.60	-0.50	-0.6	-0.2
Tmk	thymidylate kinase	A7ZKK1	23768	5.33	65	7.6E-03	48%	0.05	0.05	0.11	0.047	0.06	0.07	-1.00	-0.20	-0.6	-1.30	0.10	-0.6	0.0
Ndk	nucleoside diphosphate kinase	C4ZX93	15511	5.54	120	2.3E-08	62%	0.15	0.14	0.11	0.07	0.09	0.24	0.40	0.80	0.6	-0.60	1.50	0.5	-0.1
Amn	AMP nucleosidase	P0AE13	54246	5.9	104	3.4E-06	35%	0.10	0.044	0.036	0.05	0.05	0.05	1.50	1.00	1.3	0.50	0.00	0.3	-1.2
**Fatty acid biosynthesis (7 proteins)**							**Total RPM:**	**1.28**	**1.05**	**1.81**	**1.12**	**2.18**	**1.15**	-0.5	-0.8	**-0.6**	-0.7	-0.9	**-0.8**	**-0.3**
AccA	acetyl-coenzyme A carboxylase carboxyl transferase subunit alpha	A7ZHS5	35333	5.76	212	1.4E-17	57%	0.08	0.07	0.13	0.08	0.18	0.10	-0.60	-1.10	-0.9	-0.60	-0.90	-0.8	-0.2
AccC	acetyl-CoA carboxylase, biotin carboxylase subunit	P24182	49745	6.65	210	8.5E-17	43%	0.15	0.10	0.24	0.09	0.23	0.11	-0.70	-0.70	-0.7	-1.40	-1.10	-1.3	-0.6
FabH	3-oxoacyl-[acyl-carrier-protein] synthase 3	P0A6R0	33779	5.08	63	1.0E-02	19%	0.041	0.032	0.09	0.034	0.07	0.036	-1.10	-0.80	-1.0	-1.30	-1.00	-1.2	-0.4
FabB	3-oxoacyl-[acyl-carrier-protein] synthase I	P0A953	42928	5.35	259	1.1E-21	64%	0.22	0.14	0.34	0.25	0.48	0.25	-0.60	-1.10	-0.9	-0.50	-0.90	-0.7	-0.7
FabF	3-oxoacyl-[acyl-carrier-protein] synthase 2	P0AAI5	43247	5.71	86	5.5E-05	41%	0.31	0.33	0.50	0.37	0.52	0.32	-0.70	-0.70	-0.7	-0.40	-0.70	-0.6	0.1
FabI	enoyl-[acyl-carrier-protein] reductase [NADH] FabI	P0AEK4	28074	5.58	117	4.6E-08	51%	0.32	0.27	0.36	0.23	0.36	0.27	-0.20	-0.20	-0.2	-0.70	-0.40	-0.6	-0.2
FabZ	(3R)-hydroxymyristoyl-[acyl-carrier-protein] dehydratase	A7ZHS0	17136	6.84	71	4.5E-02	25%	0.15	0.11	0.16	0.08	0.35	0.07	0.00	-1.20	-0.6	-1.00	-2.30	-1.7	-0.5

Table S1 - Quantitative data of individual proteins of *E. coli* BL21 (DE3) growing in defined and rich media

Name[1]	Protein name[1]	Uniprot ID[1]	Molecular mass[2] (Da)	Calculated pI value[2]	Mascot Score[2]	Mascot Expect Value[2]	Mascot Seq. Cover.[2]	Defined medium: DNB medium[7] exp. phase	Defined medium: DNB medium[7] stationary phase	Rich medium: TB medium[8] exp. phase	Rich medium: TB medium[8] stationary phase	Rich medium: LB medium[9] exp. phase	Rich medium: LB medium[9] stationary phase	Log_2 (DNB /TB) at exp. phase[4]	Log_2 (DNB /LB) at exp. phase[4]	**Log_2 (defined/ rich)[4]** at exp. phase[4]	Log_2(stationary phase /exp. phase)[5, 6] in TB medium	Log_2(stationary phase /exp. phase)[5, 6] in LB medium	**Log_2(stationary phase /exp. phase)[5, 6] in rich media[5]**	**Log_2(stationary phase /exp. phase)[5, 6] in defined medium[6]**
								Relative Protein Mass (RPM)[3] - %												
**Lipopolysaccharide biosynthesis (10 proteins)**							**Total RPM:**	**0.90**	**0.89**	**1.28**	**1.24**	**0.99**	**0.91**	-0.5	-0.1	**-0.3**	0.0	-0.1	**-0.1**	**0.0**
GmhA	phosphoheptose isomerase	C2DM95	21686	5.97	142	5.3E-10	63%	0.05	0.06	0.046	0.09	0.045	0.09	0.10	0.20	0.2	1.00	0.90	1.0	0.1
HldE	bifunctional heptose 7-phosphate kinase/heptose 1-phosphate	Q8FDH5	51232	5.29	122	5.3E-08	31%	0.047	0.048	0.06	0.06	0.07	0.06	-0.40	-0.50	-0.5	0.00	-0.20	-0.1	0.0
HldD	ADP-L-glycero-D-manno-heptose-6-epimerase	C4ZXL1	34985	4.8	83	1.2E-04	41%	0.18	0.26	0.18	0.21	0.09	0.16	0.00	1.10	0.6	0.20	0.90	0.6	0.5
LpxA	acyl-[acyl-carrier-protein]-UDP-N-acetylglucosamine O-acyltransferase	C4ZRS3	28348	6.63	58	3.8E-02	32%	0.07	0.06	0.06	0.047	0.06	0.034	0.30	0.30	0.3	-0.30	-0.80	-0.6	-0.3
ArnA	fused UDP-L-Ara4N formyltransferase/UDP-GlcA C-4'-	P77398	74869	6.39	185	2.7E-14	39%	0.07	0.028	0.05	0.049	0.08	0.031	0.30	-0.40	-0.1	-0.20	-1.50	-0.9	-1.2
GlmS	glucosamine-fructose-6-phosphate aminotransferase [isomerizing]	P17169	67081	5.56	220	2.3E-18	38%	0.22	0.17	0.52	0.44	0.38	0.26	-1.30	-0.80	-1.1	-0.20	-0.50	-0.4	-0.4
GlmM	phosphoglucosamine mutase	P31120	47799	5.71	140	8.5E-10	36%	0.049	0.038	0.07	0.11	0.06	0.09	-0.60	-0.40	-0.5	0.60	0.50	0.6	-0.4
GlmU	bifunctional N-acetylglucosamine-1-phosphate uridyltransferase/glucosamine-1-	P0ACC8	49384	6.2	116	2.1E-07	30%	0.05	0.041	0.08	0.047	0.07	0.031	-0.50	-0.50	-0.5	-0.70	-1.30	-1.0	-0.4
RmlA	glucose-1-phosphate thymidylyltransferase	P55253	32703	5.27	114	9.1E-08	48%	0.07	0.06	0.08	0.05	0.049	0.06	-0.10	0.60	0.3	-0.60	0.20	-0.2	-0.2
RfbB	dTDP-glucose 4,6-dehydratase	P55293	40787	5.09	122	1.4E-08	44%	0.09	0.13	0.13	0.13	0.08	0.10	-0.50	0.10	-0.2	0.00	0.30	0.2	0.5
**Synthesis of other cellular components (16 proteins)**							**Total RPM:**	**1.08**	**0.98**	**1.07**	**0.97**	**0.94**	**0.98**	0.0	0.2	**0.1**	-0.1	0.1	**0.0**	**-0.1**
HemB	delta-aminolevulinic acid dehydratase	B2NAC8	35962	5.25	95	2.4E-05	31%	0.08	0.13	0.13	0.13	0.14	0.08	-0.70	-0.80	-0.8	0.00	-0.90	-0.5	0.8
HemL	glutamate-1-semialdehyde 2,1-aminomutase	C4ZRP7	45907	4.73	127	4.6E-09	36%	0.06	0.05	0.08	0.06	0.06	0.05	-0.40	0.00	-0.2	-0.40	-0.10	-0.3	-0.1
IspG	4-hydroxy-3-methylbut-2-en-1-yl diphosphate synthase	A7ZPV8	40943	5.87	146	5.7E-11	44%	0.09	0.06	0.11	0.09	0.12	0.06	-0.30	-0.40	-0.4	-0.30	-0.90	-0.6	-0.6
IspE	4-diphosphocytidyl-2-C-methyl-D-erythritol kinase	Q8FI04	31182	5.13	69	2.9E-03	33%	0.06	0.09	0.09	0.08	0.06	0.08	-0.60	0.00	-0.3	-0.10	0.50	0.2	0.7
Mpl	UDP-N-acetylmuramate:L-alanyl-gamma-D-glutamyl-meso-diaminopimelate ligase	P37773	50298	5.53	64	8.3E-03	28%	0.036	0.030	0.049	0.050	0.041	0.042	-0.40	-0.20	-0.3	0.00	0.00	0.0	-0.2
GshB	glutathione synthetase	P04425	35766	5.11	154	9.1E-12	47%	0.06	0.06	0.09	0.11	0.08	0.07	-0.80	-0.50	-0.7	0.20	-0.10	0.1	0.2
MdoG	glucan biosynthesis protein, periplasmic	P33136	57876	6.7	89	1.0E-04	22%	0.10	0.07	0.10	0.07	0.10	0.07	0.00	0.00	0.0	-0.40	-0.50	-0.5	-0.4
SpeA	biosynthetic arginine decarboxylase	P21170	74308	4.83	76	5.5E-04	16%	0.09	0.06	0.12	0.08	0.07	0.11	-0.40	0.20	-0.1	-0.50	0.50	0.0	-0.6
SpeE	spermidine synthase	B1XC96	32643	5.33	87	4.3E-05	38%	0.07	0.07	0.044	0.040	0.036	0.06	0.60	0.90	0.8	-0.20	0.70	0.3	0.1

Table S1 - Quantitative data of individual proteins of *E. coli* BL21 (DE3) growing in defined and rich media

Name[1]	Protein name[1]	Uniprot ID[1]	Molecular mass[2] (Da)	Calculated pI value[2]	Mascot Score[2]	Mascot Expect Value[2]	Mascot Seq. Cover.[2]	Defined medium: DNB medium[7]		Rich medium: TB medium[8]		Rich medium: LB medium[9]		Log_2 (DNB /TB)	Log_2 (DNB /LB)	**Log_2 (defined/ rich)[4]**	Log_2(stationary phase /exp. phase)[5, 6]			
								exp. phase	stationary phase	exp. phase	stationary phase	exp. phase	stationary phase				in TB medium	in LB medium	**in rich media[5]**	**in defined medium[6]**
								Relative Protein Mass (RPM)[3] - %						**at exp. phase[4]**						
LuxS	S-ribosylhomocysteine lyase	C4ZYT7	19575	5.18	77	4.4E-04	54%	0.048	0.08	0.05	0.06	0.044	0.09	-0.10	0.10	0.0	0.10	1.10	0.6	0.8
NadE	NH(3)-dependent NAD(+) synthetase	B1XGK1	30789	5.41	63	1.0E-02	39%	0.06	0.06	0.06	0.06	0.039	0.049	-0.10	0.60	0.3	-0.10	0.30	0.1	0.1
PanB	3-methyl-2-oxobutanoate hydroxymethyltransferase	A7ZHM4	28389	5.16	63	1.1E-02	26%	0.036	0.038	0.036	0.037	0.031	0.06	0.00	0.20	0.1	0.10	1.00	0.6	0.1
PanC	pantothenate synthetase	C4ZRM6	31692	5.91	109	2.9E-07	42%	0.07	0.06	0.049	0.07	0.05	0.06	0.40	0.30	0.4	0.50	0.20	0.4	-0.2
ThiC	phosphomethylpyrimidine synthase	B7NRS7	71320	5.66	66	6.3E-03	11%	0.15	0.06	0.047	0.013	0.05	0.013	1.70	1.50	1.6	-1.80	-2.10	-2.0	-1.3
GlgB	1,4-alpha-glucan branching enzyme	P07762	84398	5.91	71	7.0E-03	19%	0.044	0.029	0.013	0.017	0.020	0.045	1.80	1.10	1.5	0.40	1.10	0.8	-0.6
OtsA	alpha,alpha-trehalose-phosphate synthase [UDP-forming]	B1X658	53749	6.37	91	1.7E-05	35%	0.06	0.013	0.014	0.017	0.00	0.034	2.00	>5	>2.5	0.20	>5	-	-2.1
**(Metabolite) degradation (19 proteins)**							**Total RPM:**	**1.46**	**1.41**	**1.35**	**3.92**	**1.32**	**2.63**	0.1	0.1	**0.1**	1.5	1.0	**1.3**	**-0.1**
MalP	maltodextrin phosphorylase	P00490	90865	6.94	118	3.6E-08	20%	0.049	0.030	0.18	0.08	0.15	0.036	-1.90	-1.60	-1.8	-1.30	-2.00	-1.7	-0.7
MalQ	4-alpha-glucanotransferase	P15977	79080	6.14	123	1.1E-08	28%	0.019	0.025	0.18	0.07	0.15	0.020	-3.20	-3.00	-3.1	-1.30	-2.90	-2.1	0.4
Pgm	phosphoglucomutase	P36938	58610	5.43	75	7.8E-04	19%	0.17	0.12	0.11	0.14	0.11	0.10	0.60	0.60	0.6	0.30	-0.10	0.1	-0.5
BglX	periplasmic beta-glucosidase precursor	Q8CVX0	83562	5.85	113	4.2E-07	27%	0.08	0.035	0.08	0.07	0.06	0.050	0.10	0.40	0.3	-0.10	-0.40	-0.3	-1.3
GatZ	putative tagatose-6-phosphate kinase	B3X944	47440	5.52	74	3.8E-03	33%	0.41	0.51	0.15	1.75	0.23	0.77	1.40	0.90	1.2	3.50	1.80	2.7	0.3
GatD	galactitol-1-phosphate 5-dehydrogenase	P0A9S3	37822	5.94	61	2.0E-02	17%	0.22	0.17	0.08	0.64	0.08	0.41	1.40	1.40	1.4	3.00	2.30	2.7	-0.3
XylA	xylose isomerase	C4ZXF6	49939	5.75	63	1.2E-02	24%	0.029	0.031	0.037	0.00	0.00	0.024	-0.40	>5	-	<-5	>5	-	0.1
TreA	periplasmic trehalase	C4ZTN8	63825	5.6	213	1.1E-17	46%	0.06	0.047	0.036	0.038	0.027	0.08	0.80	1.20	1.0	0.00	1.60	0.8	-0.4
TreC	trehalose-6-phosphate hydrolase	P28904	64082	5.51	122	1.4E-08	25%	0.06	0.036	0.15	0.09	0.18	0.08	-1.40	-1.60	-1.5	-0.80	-1.20	-1.0	-0.7
MelA	alpha-galactosidase	P06720	51309	5.52	109	2.9E-07	23%	0.07	0.07	0.06	0.61	0.06	0.17	0.40	0.30	0.4	3.50	1.50	2.5	-0.1
GabT	4-aminobutyrate aminotransferase GabT	P22256	46202	5.78	227	4.6E-19	67%	0.00	0.06	0.023	0.025	0.00	0.15	<-5	0.00	-	0.20	>5	-	>5
GabD	succinate-semialdehyde dehydrogenase [NADP+] GabD	P25526	52030	5.44	125	7.2E-09	28%	0.10	0.06	0.08	0.14	0.10	0.19	0.30	0.10	0.2	0.80	1.00	0.9	-0.7
Prr	gamma-aminobutyraldehyde dehydrogenase	C4ZVI3	51197	5.65	80	2.1E-04	41%	0.042	0.045	0.033	0.042	0.018	0.18	0.40	1.30	0.9	0.40	3.30	1.9	0.1
UidA	beta-glucuronidase	P05804	68917	5.24	80	2.3E-04	21%	0.050	0.035	0.048	0.05	0.046	0.06	0.10	0.10	0.1	0.10	0.40	0.3	-0.5

Table S1 - Quantitative data of individual proteins of *E. coli* BL21 (DE3) growing in defined and rich media

Name[1]	Protein name[1]	Uniprot ID[1]	Molecular mass[2] (Da)	Calculated pI value[2]	Mascot Score[2]	Mascot Expect Value[2]	Mascot Seq. Cover.[2]	Defined medium: DNB medium[7] exp. phase	Defined medium: DNB medium[7] stationary phase	Rich medium: TB medium[8] exp. phase	Rich medium: TB medium[8] stationary phase	Rich medium: LB medium[9] exp. phase	Rich medium: LB medium[9] stationary phase	Log2 (DNB /TB) at exp. phase[4]	Log2 (DNB /LB) at exp. phase[4]	**Log2 (defined/ rich)[4] at exp. phase[4]**	Log2(stationary phase /exp. phase)[5, 6] in TB medium	Log2(stationary phase /exp. phase)[5, 6] in LB medium	**Log2(stationary phase /exp. phase)[5, 6] in rich media[5]**	**Log2(stationary phase /exp. phase)[5, 6] in defined medium[6]**
								Relative Protein Mass (RPM)[3] - %												
CpdB	2',3'-cyclic-nucleotide 2'-phosphodiesterase/3'-nucleotidase	P08331	70902	5.45	114	9.1E-08	34%	0.025	0.026	0.029	0.05	0.016	0.044	-0.20	0.60	0.2	0.90	1.50	1.2	0.1
DkgA	2,5-diketo-D-gluconic acid reductase A	Q46857	31147	6	151	1.8E-11	53%	0.046	0.06	0.038	0.040	0.028	0.07	0.20	0.70	0.5	0.10	1.40	0.8	0.3
NanA	N-acetylneuraminate lyase	C4ZSW3	32801	5.61	119	2.9E-08	36%	0.00	0.00	0.00	0.025	0.045	0.045	0.00	<-5	-	>5	0.00	-	0.0
FadB	fatty acid oxidation complex subunit alpha	C5A020	79829	5.84	131	1.8E-09	29%	0.017	0.035	0.011	0.022	0.00	0.13	0.60	>5	>2.5	1.00	>5	>2.5	1.0
FadH	2,4-dienoyl-CoA reductase [NADPH]	P42593	73203	6.11	89	3.2E-05	9%	0.011	0.014	0.021	0.018	0.027	0.029	-0.90	-1.30	-1.1	-0.20	0.10	-0.1	0.4
**Sugar transport (7 proteins)**							**Total RPM:**	**1.07**	**1.64**	**0.94**	**1.36**	**0.84**	**1.38**	0.2	0.3	**0.3**	0.5	0.7	**0.6**	**0.6**
PtsI	phosphoenolpyruvate-protein phosphotransferase	Q8XBL3	63722	4.78	109	1.0E-06	28%	0.43	0.45	0.31	0.17	0.26	0.17	0.50	0.70	0.6	-0.90	-0.60	-0.8	0.1
PtsH	phosphohistidinoprotein-hexose phosphotransferase component of PTS	P0AA06	9114	5.65	123	4.2E-08	90%	0.20	0.30	0.07	0.29	0.16	0.19	1.50	0.40	1.0	2.00	0.30	1.2	0.5
Crr	glucose-specific enzyme IIA component of PTS	P69783	18240	4.73	104	3.6E-04	74%	0.11	0.32	0.09	0.16	0.11	0.29	0.30	0.10	0.2	0.70	1.50	1.1	1.5
ManX	PTS system mannose-specific EIIAB component	P69797	35026	5.74	143	1.1E-10	42%	0.22	0.31	0.11	0.15	0.09	0.16	1.00	1.30	1.2	0.50	0.80	0.7	0.5
MalE	maltose-binding periplasmic protein	P0AEX9	43360	5.53	65	6.9E-03	16%	0.031	0.19	0.06	0.42	0.023	0.25	-1.10	0.40	-0.4	2.70	3.40	3.1	2.6
MalK	maltose/maltodextrin import ATP-binding protein MalK	P68188	41136	6.23	88	8.0E-04	33%	0.035	0.022	0.08	0.031	0.047	0.037	-1.20	-0.40	-0.8	-1.40	-0.30	-0.9	-0.7
LamB	maltoporin (lambda receptor protein)	P02943	47469	4.72	97	1.7E-05	35%	0.045	0.05	0.21	0.13	0.15	0.28	-2.20	-1.80	-2.0	-0.70	0.80	0.1	0.2
**Amino acid and peptide transport (7 proteins)**							**Total RPM:**	**0.76**	**1.28**	**0.38**	**0.59**	**0.29**	**1.12**	1.0	1.4	**1.2**	0.6	1.9	**1.3**	**0.7**
GlnH	glutamine ABC transporter periplasmic protein	P0AEQ5	27173	8.44	75	2.8E-03	48%	0.21	0.12	0.10	0.08	0.12	0.07	1.20	0.80	1.0	-0.30	-0.80	-0.6	-0.8
ArtJ	arginine transporter subunit	P30860	26927	6.84	90	8.1E-05	38%	0.032	0.06	0.05	0.06	0.00	0.044	-0.70	>5	-	0.20	>5	>2.5	0.8
ArgT	lysine-arginine-ornithine-binding periplasmic protein	P09551	28088	5.62	94	8.5E-06	51%	0.046	0.14	0.048	0.06	0.033	0.10	0.00	0.50	0.3	0.40	1.60	1.0	1.6
LivJ	leucine/isoleucine/valine transporter subunit	P0AD96	39223	5.54	131	6.6E-09	55%	0.12	0.35	0.030	0.09	0.029	0.11	2.00	2.00	2.0	1.60	2.00	1.8	1.6
DppA	periplasmic dipeptide transport protein	P23847	60483	6.21	161	1.8E-12	44%	0.14	0.28	0.00	0.00	0.00	0.29	>5	>5	>5	0.00	>5	>2.5	1.0
OppA	periplasmic oligopeptide-binding protein	P23843	60975	6.05	150	2.3E-11	38%	0.16	0.28	0.16	0.30	0.11	0.47	0.00	0.60	0.3	0.90	2.10	1.5	0.8
OppD	oligopeptide transport ATP-binding protein OppD	P76027	37506	5.78	67	4.2E-03	41%	0.05	0.049	0.00	0.00	0.00	0.032	>5	>5	>5	0.00	>5	>2.5	0.0

Table S1 - Quantitative data of individual proteins of *E. coli* BL21 (DE3) growing in defined and rich media

Name[1]	Protein name[1]	Uniprot ID[1]	Molecular mass[2] (Da)	Calculated pI value[2]	Mascot Score[2]	Mascot Expect Value[2]	Mascot Seq. Cover.[2]	Defined medium: DNB medium[7] exp. phase	Defined medium: DNB medium[7] stationary phase	Rich medium: TB medium[8] exp. phase	Rich medium: TB medium[8] stationary phase	Rich medium: LB medium[9] exp. phase	Rich medium: LB medium[9] stationary phase	Log_2 (DNB /TB) at exp. phase[4]	Log_2 (DNB /LB) at exp. phase[4]	**Log_2 (defined/ rich)[4] at exp. phase[4]**	Log_2(stationary phase /exp. phase)[5, 6] in TB medium	Log_2(stationary phase /exp. phase)[5, 6] in LB medium	**Log_2(stationary phase /exp. phase)[5, 6] in rich media[5]**	**Log_2(stationary phase /exp. phase)[5, 6] in defined medium[6]**
								Relative Protein Mass (RPM)[3] - %												
**Other transport proteins (14 proteins)**							**Total RPM:**	**4.76**	**7.26**	**4.95**	**4.49**	**5.69**	**5.24**	-0.1	-0.3	**-0.2**	-0.1	-0.1	**-0.1**	**0.6**
CusB	copper/silver efflux system, membrane fusion protein	P77239	44277	5.93	253	4.2E-21	62%	0.032	0.10	0.00	0.00	0.00	0.00	>5	>5	>5	0.00	0.00	0.0	1.7
GsiB	glutathione ABC transporter, periplasmic glutathione-binding protein GsiB	B3IL13	56479	8.23	80	7.7E-04	22%	0.039	0.027	0.048	0.035	0.038	0.045	-0.30	0.00	-0.2	-0.40	0.20	-0.1	-0.5
LptD	exported protein required for envelope biosynthesis and integrity	P31554	89843	4.94	158	1.3E-11	27%	0.07	0.08	0.06	0.046	0.08	0.05	0.20	-0.20	0.0	-0.30	-0.50	-0.4	0.2
FadL	long-chain fatty acid transport protein	Q8XCN6	48509	5.19	84	7.1E-05	34%	0.019	0.033	0.044	0.021	0.035	0.11	-1.20	-0.80	-1.0	-1.10	1.60	0.3	0.8
OmpF	outer membrane porin 1a (Ia;b;F)	P02931	39309	4.76	197	1.7E-15	69%	2.29	2.74	1.34	1.64	2.62	2.38	0.80	-0.20	0.3	0.30	-0.10	0.1	0.3
OmpA	outer membrane protein A	P0A911	37292	5.99	92	1.3E-05	32%	1.04	2.42	1.14	1.00	0.88	1.06	-0.10	0.20	0.1	-0.20	0.30	0.1	1.2
OmpA (2)	outer membrane protein A	ZP_03085789(NCBI[11])	28830	5.98	114	9.2E-07	49%	0.55	1.03	0.81	0.89	0.71	0.77	-0.60	-0.30	-0.5	0.10	0.10	0.1	0.9
TolB	translocation protein TolB	A7ZJC2	41502	6.22	100	8.5E-06	31%	0.06	0.15	0.07	0.07	0.06	0.20	-0.30	-0.10	-0.2	0.00	1.60	0.8	1.4
TolC	outer membrane protein TolC	P02930	53708	5.46	79	3.0E-04	23%	0.10	0.11	0.16	0.09	0.14	0.07	-0.60	-0.40	-0.5	-0.90	-1.00	-1.0	0.1
OmpX	outer membrane protein X	P0A919	18648	6.56	110	8.3E-07	68%	0.26	0.26	0.70	0.28	0.59	0.19	-1.40	-1.20	-1.3	-1.30	-1.60	-1.5	0.0
YbiT	putative ATP-binding component of a transport system	P0A9U5	59877	4.99	123	4.2E-08	31%	0.06	0.034	0.10	0.029	0.047	0.027	-0.80	0.30	-0.3	-1.70	-0.80	-1.3	-0.7
YjjK	uncharacterized ABC transporter ATP-binding protein YjjK	P0A9W3	62518	5.43	118	3.6E-08	34%	0.08	0.07	0.15	0.14	0.12	0.17	-0.90	-0.70	-0.8	0.00	0.40	0.2	-0.2
SecA	preprotein translocase subunit, ATPase	P10408	102187	5.43	196	2.1E-15	36%	0.06	0.09	0.22	0.17	0.24	0.08	-1.80	-1.90	-1.9	-0.40	-1.60	-1.0	0.5
YaeT	outer membrane protein assembly factor YaeT	A7ZHR7	90611	4.93	161	1.5E-12	26%	0.11	0.13	0.12	0.08	0.13	0.10	-0.10	-0.20	-0.2	-0.60	-0.30	-0.5	0.2
**RNA polymerases (3 proteins)**							**Total RPM:**	**1.18**	**1.01**	**1.48**	**0.74**	**1.72**	**0.71**	-0.3	-0.5	**-0.4**	-1.0	-1.3	**-1.1**	**-0.2**
RpoA	DNA-directed RNA polymerase subunit alpha	P0A7Z6	36717	4.98	247	1.7E-20	69%	0.51	0.53	0.74	0.38	0.73	0.35	-0.50	-0.50	-0.5	-1.00	-1.10	-1.1	0.1
RpoB	DNA-directed RNA polymerase subunit beta	P0A8V4	150935	5.15	117	1.7E-07	18%	0.62	0.45	0.70	0.31	0.86	0.33	-0.20	-0.50	-0.4	-1.20	-1.40	-1.3	-0.4
RpoC	DNA-directed RNA polymerase subunit beta'	B1XBZ0	155918	6.67	160	2.3E-12	18%	0.06	0.030	0.038	0.044	0.13	0.028	0.60	-1.10	-0.3	0.20	-2.20	-1.0	-1.0
**RNA polymerase binding proteins (7 proteins)**							**Total RPM:**	**0.94**	**0.87**	**1.40**	**0.95**	**1.55**	**0.92**	-0.6	-0.7	**-0.6**	-0.6	-0.8	**-0.7**	**-0.1**
RapA	RNA polymerase-associated protein RapA	A7ZHF0	110057	5.04	65	6.9E-03	12%	0.019	0.013	0.08	0.021	0.07	0.018	-2.10	-1.90	-2.0	-2.00	-2.00	-2.0	-0.6
NusA	transcription elongation protein NusA	P0AFF8	55008	4.53	77	4.8E-04	20%	0.13	0.11	0.19	0.08	0.21	0.09	-0.60	-0.70	-0.7	-1.30	-1.10	-1.2	-0.2

Table S1 - Quantitative data of individual proteins of *E. coli* BL21 (DE3) growing in defined and rich media

Name[1]	Protein name[1]	Uniprot ID[1]	Molecular mass[2] (Da)	Calculated pI value[2]	Mascot Score[2]	Mascot Expect Value[2]	Mascot Seq. Cover.[2]	Defined medium: DNB medium[7] exp. phase	Defined medium: DNB medium[7] stationary phase	Rich medium: TB medium[8] exp. phase	Rich medium: TB medium[8] stationary phase	Rich medium: LB medium[9] exp. phase	Rich medium: LB medium[9] stationary phase	Log_2 (DNB /TB) at exp. phase[4]	Log_2 (DNB /LB) at exp. phase[4]	**Log_2 (defined/ rich)[4] at exp. phase[4]**	Log_2(stationary phase /exp. phase)[5, 6] in TB medium	Log_2(stationary phase /exp. phase)[5, 6] in LB medium	**Log_2(stationary phase /exp. phase)[5, 6] in rich media[5]**	**Log_2(stationary phase /exp. phase)[5, 6] in defined medium[6]**
								Relative Protein Mass (RPM)[3] - %												
NusG	Transcription termination/antitermination protein NusG	P0AFG0	20518	6.34	141	1.8E-10	69%	0.07	0.07	0.10	0.045	0.13	0.06	-0.40	-0.90	-0.7	-1.10	-1.10	-1.1	0.0
Rho	transcription termination factor Rho	P0AG32	47032	6.75	166	5.7E-13	33%	0.47	0.35	0.72	0.47	0.88	0.43	-0.60	-0.90	-0.8	-0.60	-1.00	-0.8	-0.4
RpoD	RNA polymerase sigma-subunit	Q59371	69157	4.87	97	1.6E-05	31%	0.06	0.07	0.10	0.06	0.07	0.07	-0.70	-0.10	-0.4	-0.90	0.00	-0.5	0.1
SspA	stringent starvation protein A	P0ACA3	24346	5.22	128	3.6E-09	58%	0.10	0.14	0.15	0.21	0.13	0.17	-0.50	-0.30	-0.4	0.50	0.40	0.5	0.4
DksA	DnaK transcriptional regulator DksA	P0ABS3	17745	5.06	72	6.0E-03	64%	0.08	0.12	0.06	0.07	0.06	0.08	0.50	0.40	0.5	0.20	0.30	0.3	0.6
**Transcription factors (11 proteins)**							**Total RPM:**	**1.77**	**1.66**	**1.64**	**1.65**	**1.92**	**2.07**	0.1	-0.1	**0.0**	0.0	0.1	**0.1**	**-0.1**
Hns	global DNA-binding transcriptional dual regulator H-NS	P0ACG0	15587	5.43	92	5.9E-05	62%	0.71	0.81	0.78	0.75	1.12	1.07	-0.10	-0.70	-0.4	-0.10	-0.10	-0.1	0.2
ArcA	aerobic respiration control protein ArcA	P0A9Q1	27389	5.21	156	5.7E-12	46%	0.07	0.11	0.08	0.07	0.09	0.21	-0.10	-0.30	-0.2	-0.20	1.20	0.5	0.5
KdgR	transcriptional regulator KdgR	P76268	30067	5.43	164	9.1E-13	74%	0.035	0.035	0.036	0.027	0.040	0.041	0.00	-0.20	-0.1	-0.40	0.00	-0.2	0.0
OxyR	hydrogen peroxide-inducible genes activator	P0ACQ4	34596	5.96	95	7.1E-06	34%	0.08	0.06	0.06	0.08	0.09	0.07	0.50	0.00	0.3	0.50	-0.40	0.1	-0.6
BasR	transcriptional regulatory protein BasR	P30843	25072	5.66	70	2.1E-03	31%	0.07	0.07	0.06	0.049	0.049	0.07	0.20	0.60	0.4	-0.30	0.40	0.1	-0.1
CspC	CspC	Q1PG46	7497	8.09	56	2.2E-01	60%	0.38	0.23	0.31	0.38	0.29	0.37	0.30	0.40	0.4	0.30	0.30	0.3	-0.7
StpA	DNA binding protein, nucleoid-associated	P0ACG2	15338	7.93	50	8.1E-01	33%	0.20	0.15	0.13	0.10	0.13	0.041	0.60	0.70	0.7	-0.40	-1.60	-1.0	-0.4
PspA	phage shock protein A	P0AFM6	25477	5.39	76	6.3E-04	57%	0.025	0.022	0.027	0.029	0.020	0.022	-0.10	0.40	0.2	0.10	0.20	0.2	-0.2
OmpR	transcriptional regulatory protein OmpR	P0AA16	27393	6.04	132	1.4E-09	52%	0.08	0.09	0.07	0.06	0.05	0.11	0.10	0.60	0.4	-0.40	1.10	0.4	0.1
CpxR	transcriptional regulatory protein CpxR	P0AE88	26296	5.39	71	1.9E-03	38%	0.06	0.06	0.047	0.06	0.042	0.048	0.30	0.50	0.4	0.40	0.20	0.3	-0.1
PhoP	transcriptional regulatory protein PhoP	P23836	25519	5.1	123	1.1E-08	51%	0.042	0.048	0.027	0.049	0.00	0.029	0.60	>5	>2.5	0.80	>5	>2.5	0.2

Table S1 - Quantitative data of individual proteins of *E. coli* BL21 (DE3) growing in defined and rich media

Name[1]	Protein name[1]	Uniprot ID[1]	Molecular mass[2] (Da)	Calculated pI value[2]	Mascot Score[2]	Mascot Expect Value[2]	Mascot Seq. Cover.[2]	Defined medium: DNB medium[7] exp. phase	Defined medium: DNB medium[7] stationary phase	Rich medium: TB medium[8] exp. phase	Rich medium: TB medium[8] stationary phase	Rich medium: LB medium[9] exp. phase	Rich medium: LB medium[9] stationary phase	Log_2 (DNB/TB) at exp. phase[4]	Log_2 (DNB/LB) at exp. phase[4]	**Log_2 (defined/rich)[4] at exp. phase[4]**	Log_2(stationary phase/exp. phase)[5, 6] in TB medium	Log_2(stationary phase/exp. phase)[5, 6] in LB medium	**Log_2(stationary phase/exp. phase)[5, 6] in rich media[5]**	**Log_2(stationary phase/exp. phase)[5, 6] in defined medium[6]**
								Relative Protein Mass (RPM)[3] - %												
**Ribosomal proteins (5 proteins)**							**Total RPM:**	**2.98**	**2.72**	**5.25**	**2.39**	**5.99**	**2.27**	-0.8	-1.0	**-0.9**	-1.1	-1.4	**-1.3**	**-0.1**
RplL	50S ribosomal protein L7/L12	P0A7K2	12069	4.6	70	7.8E-03	44%	0.70	0.79	0.76	0.45	1.15	0.56	-0.10	-0.70	-0.4	-0.70	-1.00	-0.9	0.2
RplI	50S ribosomal protein L9	P0A7R3	15759	6.17	197	1.7E-15	81%	0.49	0.39	0.81	0.33	0.81	0.36	-0.70	-0.70	-0.7	-1.30	-1.10	-1.2	-0.4
RpsA	30S ribosomal protein S1	P0AG69	61235	4.89	189	1.0E-14	35%	0.86	0.95	2.04	0.93	2.19	0.79	-1.20	-1.30	-1.3	-1.10	-1.50	-1.3	0.1
RpsB	30S ribosomal protein S2	C4ZRR1	26784	6.61	68	3.2E-03	29%	0.62	0.44	1.10	0.45	1.29	0.45	-0.80	-1.10	-1.0	-1.30	-1.50	-1.4	-0.5
RpsF	30S ribosomal protein S6	A7ZV71	15177	5.26	61	1.7E-02	39%	0.30	0.17	0.55	0.23	0.56	0.12	-0.90	-0.90	-0.9	-1.30	-2.20	-1.8	-0.9
**Ribosome-associated proteins (8 proteins)**							**Total RPM:**	**0.90**	**0.98**	**1.64**	**1.00**	**1.68**	**1.00**	-0.9	-0.9	**-0.9**	-0.7	-0.7	**-0.7**	**0.1**
EngA	predicted GTP-binding protein	P0A6P5	55058	5.6	150	8.4E-11	32%	0.039	0.011	0.041	0.022	0.029	0.031	-0.10	0.40	0.2	-0.90	0.10	-0.4	-1.9
YcaO	UPF0142 protein YcaO	P75838	66009	4.38	75	7.8E-04	13%	0.020	0.010	0.033	0.022	0.05	0.015	-0.70	-1.40	-1.1	-0.60	-1.80	-1.2	-1.1
EngD (YchF)	GTP-dependent nucleic acid-binding protein EngD	P0ABU3	39984	4.87	211	6.7E-17	60%	0.031	0.043	0.06	0.036	0.07	0.06	-1.00	-1.20	-1.1	-0.80	-0.20	-0.5	0.5
TypA	GTP-binding protein TypA/BipA	P32132	67542	5.16	71	4.1E-02	22%	0.20	0.12	0.26	0.11	0.27	0.13	-0.40	-0.40	-0.4	-1.30	-1.10	-1.2	-0.7
HflX	GTP-binding protein hflX	P25519	48468	5.68	88	8.6E-04	21%	0.031	0.022	0.11	0.023	0.08	0.036	-1.90	-1.40	-1.7	-2.30	-1.20	-1.8	-0.5
Tig	trigger factor	Q1RFA0	47836	4.83	205	2.6E-16	46%	0.45	0.46	0.96	0.45	0.85	0.47	-1.10	-0.90	-1.0	-1.10	-0.80	-1.0	0.0
Frr	ribosome recycling factor	A1A7L6	19269	6.43	66	2.3E-02	40%	0.040	0.09	0.06	0.15	0.12	0.11	-0.60	-1.60	-1.1	1.30	-0.20	0.6	1.2
RaiA	ribosome-associated inhibitor A	P0AD51	12777	6.2	62	1.4E-02	46%	0.09	0.22	0.11	0.20	0.20	0.14	-0.30	-1.20	-0.8	0.90	-0.40	0.3	1.3
**Aminoacyl-tRNA synthetases (23 proteins)**							**Total RPM:**	**3.57**	**2.86**	**5.28**	**4.58**	**4.97**	**3.32**	-0.6	-0.5	**-0.5**	-0.2	-0.6	**-0.4**	**-0.3**
AlaS	alanyl-tRNA synthetase	B1XCM5	96315	5.53	99	2.8E-06	16%	0.32	0.28	0.48	0.49	0.58	0.41	-0.60	-0.90	-0.8	0.00	-0.50	-0.3	-0.2
ArgS	arginyl-tRNA synthetase	Q8XCH2	64851	5.31	131	6.7E-09	26%	0.07	0.05	0.13	0.06	0.14	0.06	-0.90	-1.00	-1.0	-1.20	-1.30	-1.3	-0.4
AsnS	asparagine-tRNA ligase	A7ZK21	52766	5.17	131	1.8E-09	32%	0.27	0.18	0.29	0.19	0.32	0.17	-0.10	-0.20	-0.2	-0.70	-0.90	-0.8	-0.5
AspS	aspartyl-tRNA synthetase	Q8XCI7	66115	5.47	59	3.0E-02	20%	0.20	0.15	0.26	0.18	0.22	0.17	-0.40	-0.10	-0.3	-0.50	-0.30	-0.4	-0.4
CysS	cysteinyl-tRNA synthetase	Q8FK44	52436	5.33	76	2.0E-03	28%	0.24	0.10	0.13	0.22	0.11	0.16	0.90	1.10	1.0	0.70	0.60	0.7	-1.2

Table S1 - Quantitative data of individual proteins of *E. coli* BL21 (DE3) growing in defined and rich media

Name[1]	Protein name[1]	Uniprot ID[1]	Molecular mass[2] (Da)	Calculated pI value[2]	Mascot Score[2]	Mascot Expect Value[2]	Mascot Seq. Cover.[2]	Defined medium: DNB medium[7] exp. phase	Defined medium: DNB medium[7] stationary phase	Rich medium: TB medium[8] exp. phase	Rich medium: TB medium[8] stationary phase	Rich medium: LB medium[9] exp. phase	Rich medium: LB medium[9] stationary phase	Log_2 (DNB /TB) at exp. phase[4]	Log_2 (DNB /LB) at exp. phase[4]	**Log_2 (defined/ rich)[4] at exp. phase[4]**	Log_2(stationary phase /exp. phase)[5, 6] in TB medium	Log_2(stationary phase /exp. phase)[5, 6] in LB medium	**in rich media[5]**	**in defined medium[6]**
								Relative Protein Mass (RPM)[3] - %												
GlnS	glutaminyl-tRNA synthetase	Q8X9H8	64040	5.88	109	1.1E-06	31%	0.15	0.08	0.17	0.12	0.18	0.15	-0.20	-0.30	-0.3	-0.50	-0.20	-0.4	-0.9
GltX	glutamyl-tRNA synthetase	B1X9R9	54181	5.59	58	3.2E-02	24%	0.15	0.10	0.26	0.17	0.24	0.16	-0.80	-0.70	-0.8	-0.60	-0.60	-0.6	-0.6
GlyQ	glycyl-tRNA synthetase alpha subunit	A7ZTA6	34979	4.94	106	5.7E-07	45%	0.10	0.06	0.13	0.06	0.10	0.06	-0.40	0.00	-0.2	-1.00	-0.70	-0.9	-0.6
GlyS	glycyl-tRNA synthetase beta subunit	P00961	76936	5.29	181	1.8E-14	28%	0.18	0.10	0.37	0.16	0.32	0.16	-1.10	-0.80	-1.0	-1.20	-1.00	-1.1	-0.8
HisS	histidyl-tRNA synthetase	P60908	47285	5.65	148	1.3E-10	44%	0.08	0.07	0.11	0.06	0.11	0.05	-0.40	-0.50	-0.5	-1.00	-1.10	-1.1	-0.3
IleS	isoleucyl-tRNA synthetase	P00956	105042	5.7	169	1.1E-12	33%	0.17	0.14	0.33	0.22	0.34	0.16	-1.00	-1.00	-1.0	-0.60	-1.10	-0.9	-0.2
LeuS	leucine-tRNA ligase	A7ZJ31	97768	5.16	103	1.1E-06	21%	0.21	0.21	0.36	0.16	0.29	0.17	-0.80	-0.50	-0.7	-1.10	-0.80	-1.0	0.0
LysS	lysyl-tRNA synthetase	Q8XD57	57614	5.11	159	1.1E-11	42%	0.13	0.11	0.26	0.16	0.28	0.13	-0.90	-1.00	-1.0	-0.70	-1.10	-0.9	-0.2
MetG	methionyl-tRNA synthetase	C4ZSJ8	76662	5.56	88	3.8E-05	24%	0.12	0.09	0.18	0.11	0.20	0.10	-0.60	-0.70	-0.7	-0.70	-1.00	-0.9	-0.5
PheT	phenylalanyl-tRNA synthetase subunit beta	Q8XE32	88119	5.12	181	6.6E-14	31%	0.20	0.22	0.21	0.13	0.24	0.11	-0.10	-0.30	-0.2	-0.70	-1.10	-0.9	0.1
ProS	proline-tRNA ligase	C4ZRT7	63710	5.12	214	9.1E-18	37%	0.20	0.22	0.36	0.25	0.33	0.20	-0.80	-0.70	-0.8	-0.50	-0.70	-0.6	0.1
SerS	serine-tRNA ligase	A7ZJW2	48669	5.34	84	1.0E-04	35%	0.14	0.14	0.24	0.21	0.24	0.16	-0.80	-0.80	-0.8	-0.20	-0.60	-0.4	0.0
ThrS	threonyl-tRNA synthetase	Q8XE27	74722	5.8	52	5.8E-01	17%	0.16	0.16	0.28	0.33	0.28	0.21	-0.80	-0.80	-0.8	0.30	-0.40	-0.1	0.0
TyrS	tyrosyl-tRNA synthetase	A1ABI2	48368	5.49	83	1.1E-04	39%	0.18	0.12	0.29	0.16	0.22	0.13	-0.60	-0.20	-0.4	-0.90	-0.80	-0.9	-0.6
LysU	lysyl-tRNA synthetase, heat inducible	P0A8N5	57847	5.1	166	5.7E-13	40%	0.12	0.11	0.30	0.99	0.16	0.24	-1.30	-0.40	-0.9	1.70	0.60	1.2	-0.2
MnmA	tRNA-specific 2-thiouridylase MnmA	A7ZKS3	41333	4.94	69	2.9E-03	33%	0.05	0.045	0.06	0.035	0.045	0.032	-0.10	0.20	0.1	-0.60	-0.50	-0.6	-0.2
YgfZ	tRNA-modifying protein YgfZ	C5A0H0	36185	5.17	211	1.8E-17	54%	0.046	0.06	0.06	0.08	0.038	0.08	-0.30	0.30	0.0	0.50	1.10	0.8	0.3
Fmt	methionyl-tRNA formyltransferase	P23882	34318	5.56	65	7.8E-03	22%	0.07	0.05	0.018	0.027	0.011	0.041	2.00	2.70	2.4	0.60	1.90	1.3	-0.5
**Elongation factors (6 proteins)**							**Total RPM:**	**6.00**	**6.61**	**9.25**	**7.02**	**9.68**	**5.83**	-0.6	-0.7	**-0.7**	-0.4	-0.7	**-0.6**	**0.1**
InfB	initiation factor IF2-gamma	P0A705	78978	5.65	79	1.1E-03	26%	0.14	0.11	0.29	0.13	0.30	0.14	-1.10	-1.10	-1.1	-1.20	-1.10	-1.2	-0.3
FusA	elongation factor G	A7ZSL5	77704	5.24	102	1.4E-06	23%	1.49	1.59	2.10	1.64	2.25	1.23	-0.50	-0.60	-0.6	-0.40	-0.90	-0.7	0.1
TufA	elongation factor Tu	A7ZSL4	43427	5.3	109	2.9E-07	28%	3.51	4.09	5.49	4.51	5.83	3.63	-0.60	-0.70	-0.7	-0.30	-0.70	-0.5	0.2

Table S1 - Quantitative data of individual proteins of *E. coli* BL21 (DE3) growing in defined and rich media

Name[1]	Protein name[1]	Uniprot ID[1]	Molecular mass[2] (Da)	Calculated pI value[2]	Mascot Score[2]	Mascot Expect Value[2]	Mascot Seq. Cover.[2]	Defined medium: DNB medium[7] exp. phase	Defined medium: DNB medium[7] stationary phase	Rich medium: TB medium[8] exp. phase	Rich medium: TB medium[8] stationary phase	Rich medium: LB medium[9] exp. phase	Rich medium: LB medium[9] stationary phase	Log2 (DNB /TB) at exp. phase[4]	Log2 (DNB /LB) at exp. phase[4]	Log2 (defined/ rich)[4] at exp. phase[4]	Log2(stationary phase /exp. phase)[5, 6] in TB medium	Log2(stationary phase /exp. phase)[5, 6] in LB medium	Log2(stationary phase /exp. phase)[5, 6] in rich media[5]	Log2(stationary phase /exp. phase)[5, 6] in defined medium[6]
								Relative Protein Mass (RPM)[3] - %												
Tsf	elongation factor Ts	P0A6P1	30387	5.22	245	2.6E-20	73%	0.79	0.76	1.22	0.66	1.17	0.77	-0.60	-0.60	-0.6	-0.90	-0.60	-0.8	-0.1
LepA	GTP-binding protein LepA	P60787	67099	5.4	193	4.2E-15	40%	0.034	0.027	0.07	0.045	0.07	0.024	-1.00	-1.10	-1.1	-0.60	-1.50	-1.1	-0.3
PrfC	peptide chain release factor 3	Q1R270	59632	5.66	75	2.9E-03	20%	0.038	0.035	0.08	0.037	0.048	0.042	-1.00	-0.30	-0.7	-1.10	-0.20	-0.7	-0.1
**RNA degradation (3 proteins)**						**Total RPM:**		**0.52**	**0.34**	**0.77**	**0.34**	**0.69**	**0.39**	-0.6	-0.4	**-0.5**	-1.2	-0.8	**-1.0**	**-0.6**
Pnp	polyribonucleotide nucleotidyltransferase	B1IQV7	77093	5.09	100	2.6E-06	15%	0.38	0.26	0.55	0.27	0.51	0.28	-0.50	-0.40	-0.5	-1.00	-0.80	-0.9	-0.5
Rnb	exoribonuclease II	B3XC13	72829	5.44	136	2.1E-09	30%	0.10	0.06	0.18	0.06	0.12	0.07	-0.80	-0.30	-0.6	-1.60	-0.80	-1.2	-0.8
RhlB	ATP-dependent RNA helicase	P0A8J8	47325	7.29	104	3.4E-06	29%	0.039	0.016	0.046	0.013	0.06	0.032	-0.20	-0.70	-0.5	-1.80	-1.00	-1.4	-1.3
**Isomerases (5 proteins)**						**Total RPM:**		**0.55**	**0.63**	**0.67**	**0.63**	**0.52**	**0.70**	-0.3	0.1	**-0.1**	-0.1	0.4	**0.2**	**0.2**
PpiB	peptidyl-prolyl cis-trans isomerase B (rotamase B)	Q8XCU0	18270	5.52	62	5.5E-02	38%	0.09	0.17	0.10	0.23	0.13	0.18	-0.10	-0.60	-0.4	1.30	0.50	0.9	1.0
SurA	peptidyl-prolyl cis-trans isomerase SurA	P0ABZ8	47254	6.48	135	2.7E-09	28%	0.09	0.11	0.11	0.09	0.06	0.11	-0.30	0.60	0.2	-0.30	1.00	0.4	0.3
FklB	FKBP-type 22 kDa peptidyl-prolyl cis-trans isomerase	P0A9L3	22203	4.85	146	4.7E-11	37%	0.05	0.06	0.08	0.06	0.05	0.06	-0.60	0.00	-0.3	-0.50	0.20	-0.2	0.3
FkpA	FKBP-type peptidyl-prolyl cis-trans isomerase	P65765	28894	8.39	94	3.3E-05	44%	0.22	0.23	0.25	0.14	0.16	0.21	-0.20	0.40	0.1	-0.80	0.40	-0.2	0.1
SlyD	FKBP-type peptidyl-prolyl cis-trans isomerase SlyD	P0A9L1	21182	4.86	100	2.6E-06	31%	0.10	0.05	0.13	0.11	0.12	0.13	-0.40	-0.30	-0.4	-0.20	0.10	-0.1	-0.9
**Chaperones (9 proteins)**						**Total RPM:**		**2.83**	**3.11**	**3.94**	**4.48**	**4.08**	**4.06**	-0.5	-0.5	**-0.5**	0.2	0.0	**0.1**	**0.1**
DnaK	molecular chaperone DnaK	P0A6Z0	69130	4.83	192	5.3E-15	39%	0.69	0.86	1.17	1.26	1.11	1.16	-0.70	-0.70	-0.7	0.10	0.10	0.1	0.3
GrpE	heat shock protein GrpE	A7ZQ54	21727	4.68	54	3.3E-01	32%	0.10	0.10	0.16	0.17	0.18	0.14	-0.70	-0.90	-0.8	0.10	-0.30	-0.1	0.0
GroL	60 kDa chaperonin (GroEL protein)	P0A6F5	55224	4.84	219	1.0E-17	52%	1.12	1.41	1.60	1.88	1.83	1.68	-0.50	-0.70	-0.6	0.20	-0.10	0.1	0.3
GroS	co-chaperonin GroES	P0A6G1	10381	5.15	100	8.3E-06	64%	0.30	0.30	0.33	0.43	0.25	0.49	-0.10	0.30	0.1	0.40	0.90	0.7	0.0
HtpG	heat shock protein 90	P0A6Z5	71378	5.09	262	5.3E-22	50%	0.27	0.22	0.42	0.31	0.37	0.26	-0.60	-0.40	-0.5	-0.50	-0.50	-0.5	-0.3
HscA	Fe-S protein assembly chaperone HscA	B3XDI5	65726	5.02	85	2.9E-04	21%	0.046	0.018	0.07	0.026	0.06	0.024	-0.70	-0.30	-0.5	-1.50	-1.20	-1.4	-1.4
ClpA	ATP-dependent Clp protease ATP-binding subunit	P0ABI1	84326	5.91	87	1.6E-04	21%	0.049	0.019	0.06	0.037	0.10	0.050	-0.30	-1.00	-0.7	-0.80	-1.00	-0.9	-1.3

Table S1 - Quantitative data of individual proteins of *E. coli* BL21 (DE3) growing in defined and rich media

Name[1]	Protein name[1]	Uniprot ID[1]	Molecular mass[2] (Da)	Calculated pI value[2]	Mascot Score[2]	Mascot Expect Value[2]	Mascot Seq. Cover.[2]	Defined medium: DNB medium[7] exp. phase	Defined medium: DNB medium[7] stationary phase	Rich medium: TB medium[8] exp. phase	Rich medium: TB medium[8] stationary phase	Rich medium: LB medium[9] exp. phase	Rich medium: LB medium[9] stationary phase	Log_2 (DNB /TB) at exp. phase[4]	Log_2 (DNB /LB) at exp. phase[4]	**Log_2 (defined/ rich)[4]** at exp. phase[4]	Log_2(stationary phase /exp. phase)[5, 6] in TB medium	Log_2(stationary phase /exp. phase)[5, 6] in LB medium	**in rich media[5]**	**in defined medium[6]**
								Relative Protein Mass (RPM)[3] - %												
ClpB	protein disaggregation chaperone	P63285	95697	5.37	160	8.3E-12	27%	0.11	0.08	0.08	0.19	0.09	0.10	0.50	0.30	0.4	1.20	0.20	0.7	-0.5
IbpA	small heat shock protein IbpA	A7ZTP1	15764	5.57	168	8.4E-12	70%	0.13	0.10	0.040	0.19	0.10	0.15	1.70	0.40	1.1	2.30	0.50	1.4	-0.4
**Proteases (13 proteins)**							**Total RPM:**	**1.23**	**1.10**	**1.55**	**1.91**	**1.37**	**1.75**	-0.3	-0.2	**-0.2**	0.3	0.4	**0.3**	**-0.2**
DegP	serine endoprotease	P0C0V1	49438	8.65	119	1.1E-07	31%	0.029	0.024	0.00	0.019	0.00	0.041	>5	>5	>5	>5	>5	>5	-0.2
PepQ	Xaa-Pro dipeptidase	A7ZU52	50315	5.6	103	1.1E-06	28%	0.13	0.13	0.12	0.22	0.08	0.19	0.10	0.60	0.4	1.00	1.20	1.1	0.1
PepD	aminoacyl-histidine dipeptidase	P15288	53110	5.2	78	8.0E-03	27%	0.19	0.14	0.26	0.61	0.19	0.40	-0.50	0.00	-0.3	1.20	1.10	1.2	-0.4
PepN	aminopeptidase N	B7UN18	99343	5.09	131	6.7E-09	22%	0.13	0.12	0.13	0.17	0.12	0.22	0.00	0.10	0.1	0.40	0.90	0.7	-0.1
Dcp	dipeptidyl carboxypeptidase II	Q8XB30	77516	5.35	189	1.1E-14	33%	0.033	0.045	0.05	0.08	0.025	0.11	-0.70	0.40	-0.2	0.60	2.20	1.4	0.5
Prc	carboxy-terminal protease	Q321Y5	76514	6.38	105	2.7E-06	28%	0.05	0.024	0.041	0.021	0.049	0.037	0.30	0.10	0.2	-0.90	-0.40	-0.7	-1.1
PmbA	protein PmbA	P0AFK0	48625	5.4	124	9.1E-09	35%	0.049	0.07	0.05	0.07	0.05	0.05	-0.10	-0.10	-0.1	0.40	0.00	0.2	0.4
ClpP	ATP-dependent Clp protease proteolytic subunit	P0A6G9	23286	5.52	63	4.6E-02	30%	0.10	0.09	0.10	0.11	0.12	0.13	-0.10	-0.40	-0.3	0.10	0.00	0.1	-0.1
HslV	ATP-dependent protease peptidase subunit	P0A7C0	19138	5.96	79	1.0E-03	46%	0.06	0.045	0.07	0.046	0.08	0.08	-0.20	-0.40	-0.3	-0.60	-0.10	-0.4	-0.4
HslU	ATP-dependent protease ATP-binding subunit	P0A6H6	49677	5.24	166	2.3E-10	48%	0.11	0.16	0.18	0.13	0.19	0.14	-0.70	-0.70	-0.7	-0.40	-0.40	-0.4	0.5
PepB	peptidase B	B7UGW9	46483	5.49	69	2.6E-03	29%	0.20	0.07	0.23	0.16	0.19	0.12	-0.30	0.00	-0.2	-0.60	-0.70	-0.7	-1.4
PepP	Xaa-Pro aminopeptidase	P15034	50012	5.25	84	8.5E-05	27%	0.09	0.11	0.17	0.14	0.17	0.11	-0.90	-1.00	-1.0	-0.20	-0.60	-0.4	0.4
PrlC	oligopeptidase A	P27298	77461	5.15	209	2.9E-17	41%	0.07	0.06	0.14	0.12	0.10	0.12	-1.00	-0.50	-0.8	-0.20	0.30	0.1	-0.2
**Other dehydrogenases (9 proteins)**							**Total RPM:**	**0.93**	**0.78**	**0.79**	**0.79**	**0.76**	**1.02**	0.2	0.3	**0.3**	0.0	0.4	**0.2**	**-0.2**
YdfG	3-hydroxy acid dehydrogenase	Q8X505	27360	5.65	86	2.1E-04	40%	0.10	0.11	0.07	0.10	0.047	0.08	0.60	1.10	0.9	0.70	0.70	0.7	0.2
YqhD	alcohol dehydrogenase, NAD(P)-dependent	Q46856	42128	5.72	83	4.5E-04	32%	0.20	0.16	0.19	0.19	0.16	0.16	0.10	0.40	0.3	0.00	0.10	0.1	-0.3
AldB	aldehyde dehydrogenase B	P37685	56670	5.44	62	1.3E-02	17%	0.07	0.05	0.042	0.045	0.06	0.13	0.80	0.30	0.6	0.10	1.10	0.6	-0.5
AldA	lactaldehyde dehydrogenase	P25553	52411	5.07	61	1.8E-02	20%	0.08	0.043	0.08	0.06	0.05	0.18	0.00	0.50	0.3	-0.30	1.70	0.7	-0.8

Table S1 - Quantitative data of individual proteins of *E. coli* BL21 (DE3) growing in defined and rich media

Name[1]	Protein name[1]	Uniprot ID[1]	Molecular mass[2] (Da)	Calculated pI value[2]	Mascot Score[2]	Mascot Expect Value[2]	Mascot Seq. Cover.[2]	Defined medium		Rich medium				Log$_2$ (DNB /TB)	Log$_2$ (DNB /LB)	**Log$_2$ (defined/ rich)[4]**	**Log$_2$(stationary phase /exp. phase)[5, 6]**			
								DNB medium[7]		TB medium[8]		LB medium[9]								
								exp. phase	stationary phase	exp. phase	stationary phase	exp. phase	stationary phase				in TB medium	in LB medium	**in rich media[5]**	**in defined medium[6]**
								Relative Protein Mass (RPM)[3] - %						**at exp. phase[4]**						
YhhX	predicted oxidoreductase with NAD(P)-binding Rossmann-fold domain	P46853	38912	6.07	131	6.7E-09	32%	0.035	0.026	0.030	0.034	0.046	0.042	0.20	-0.40	-0.1	0.20	-0.10	0.1	-0.4
Ugd	UDP-glucose 6-dehydrogenase	P76373	43744	6.06	76	1.5E-02	33%	0.18	0.20	0.12	0.10	0.15	0.16	0.70	0.20	0.5	-0.20	0.10	-0.1	0.1
HdhA	7-alpha-hydroxysteroid dehydrogenase	P0AET8	26990	5.22	67	4.3E-03	36%	0.048	0.044	0.037	0.07	0.049	0.07	0.40	-0.10	0.2	0.90	0.60	0.8	-0.1
HybC	hydrogenase-2 large chain	P0ACE0	62908	5.84	60	2.3E-02	25%	0.05	0.045	0.06	0.08	0.06	0.09	-0.30	-0.10	-0.2	0.20	0.60	0.4	-0.2
TrxB	thioredoxin reductase	P0A9P4	34829	5.3	128	3.6E-09	48%	0.15	0.10	0.16	0.12	0.13	0.10	-0.10	0.20	0.1	-0.50	-0.50	-0.5	-0.7
**Oxidoreductases (8 proteins)**							**Total RPM:**	**0.51**	**0.68**	**0.48**	**0.82**	**0.49**	**0.90**	0.1	0.1	**0.1**	0.8	0.9	**0.8**	**0.4**
Gor	glutathione reductase	P06715	49084	5.64	78	8.4E-03	29%	0.06	0.050	0.09	0.09	0.08	0.07	-0.60	-0.40	-0.5	0.00	0.00	0.0	-0.2
NfsA	oxygen-insensitive NADPH nitroreductase	P17117	27069	6.45	117	4.6E-08	40%	0.07	0.050	0.15	0.09	0.14	0.08	-1.10	-1.00	-1.1	-0.80	-0.90	-0.9	-0.5
QueF	NADPH-dependent 7-cyano-7-deazaguanine reductase	C4ZZU9	32909	5.73	131	1.8E-09	39%	0.06	0.08	0.07	0.036	0.037	0.043	0.00	0.80	0.4	-0.90	0.20	-0.4	0.3
NemA	N-ethylmaleimide reductase	P77258	39492	5.8	90	2.2E-05	38%	0.046	0.040	0.05	0.05	0.034	0.043	-0.20	0.40	0.1	0.00	0.30	0.2	-0.2
SthA	soluble pyridine nucleotide transhydrogenase	A7ZUI2	51984	6.08	109	6.6E-06	22%	0.11	0.05	0.05	0.12	0.08	0.19	1.10	0.40	0.8	1.20	1.20	1.2	-1.1
CueO	blue copper oxidase CueO	P36649	53557	6.07	63	4.4E-02	18%	0.042	0.06	0.037	0.06	0.040	0.044	0.20	0.10	0.2	0.70	0.10	0.4	0.5
Bfr	bacterioferritin, iron storage and detoxification protein	P0ABD3	18483	4.69	156	2.1E-11	60%	0.06	0.25	0.033	0.29	0.08	0.23	0.90	-0.30	0.3	3.20	1.50	2.4	2.0
WrbA	flavoprotein wrbA	C4ZQD2	20832	5.59	85	7.6E-05	53%	0.049	0.10	0.00	0.09	0.00	0.20	>5	>5	>5	>5	>5	>5	1.0
**Hydroperoxide reductases and superoxide dismutase (5 proteins)**							**Total RPM:**	**1.44**	**2.19**	**1.55**	**3.45**	**1.29**	**2.05**	-0.1	0.2	**0.0**	1.2	0.7	**0.9**	**0.6**
AhpF	alkyl hydroperoxide reductase subunit F	B3XD66	56496	5.47	101	6.7E-06	28%	0.10	0.08	0.12	0.10	0.10	0.07	-0.20	0.10	-0.1	-0.30	-0.50	-0.4	-0.3
AhpC	alkyl hydroperoxide reductase subunit C	P0AE08	20862	5.03	108	3.6E-07	70%	0.90	1.22	1.00	1.38	0.89	1.13	-0.20	0.00	-0.1	0.50	0.30	0.4	0.4
Tpx	thiol peroxidase	P0A864	17995	4.75	103	1.1E-06	48%	0.08	0.15	0.06	0.15	0.06	0.16	0.40	0.60	0.5	1.20	1.50	1.4	0.8
SodB	superoxide dismutase [Fe]	P0AGD5	21310	5.58	125	2.6E-08	87%	0.14	0.64	0.12	1.51	0.12	0.30	0.20	0.20	0.2	3.70	1.40	2.6	2.2
KatG	catalase-peroxidase	B1XBA8	80031	5.14	125	7.2E-09	20%	0.22	0.09	0.25	0.32	0.14	0.39	-0.20	0.70	0.3	0.40	1.50	1.0	-1.3

Table S1 - Quantitative data of individual proteins of *E. coli* BL21 (DE3) growing in defined and rich media

Name[1]	Protein name[1]	Uniprot ID[1]	Molecular mass[2] (Da)	Calculated pI value[2]	Mascot Score[2]	Mascot Expect Value[2]	Mascot Seq. Cover.[2]	Defined medium: DNB medium[7] exp. phase	Defined medium: DNB medium[7] stationary phase	Rich medium: TB medium[8] exp. phase	Rich medium: TB medium[8] stationary phase	Rich medium: LB medium[9] exp. phase	Rich medium: LB medium[9] stationary phase	Log_2 (DNB /TB) at exp. phase[4]	Log_2 (DNB /LB) at exp. phase[4]	**Log_2 (defined/ rich)[4] at exp. phase[4]**	Log_2(stationary phase /exp. phase)[5, 6] in TB medium	Log_2(stationary phase /exp. phase)[5, 6] in LB medium	**Log_2(stationary phase /exp. phase)[5, 6] in rich media[5]**	**Log_2(stationary phase /exp. phase)[5, 6] in defined medium[6]**
								Relative Protein Mass (RPM)[3] - %												
**DNA protection and repair (7 proteins)**							**Total RPM:**	**0.83**	**1.39**	**0.76**	**1.22**	**0.86**	**1.74**	0.1	-0.1	**0.0**	0.7	1.0	**0.8**	**0.8**
Ssb	single-stranded DNA-binding protein	P0AGE0	18963	5.44	132	1.4E-09	41%	0.00	0.033	0.00	0.042	0.06	0.08	0.00	<-5	-	>5	0.40	>2.5	>5
Dps	DNA starvation/stationary phase protection protein Dps	P0ABT3	18684	5.72	142	5.3E-10	66%	0.030	0.48	0.037	0.58	0.06	0.75	-0.30	-0.90	-0.6	4.00	3.70	3.9	4.0
CbpA	curved DNA-binding protein	C4ZQC8	34434	6.33	82	1.4E-04	24%	0.00	0.020	0.00	0.029	0.00	0.034	0.00	0.00	0.0	>5	>5	>5	>5
UspA	universal stress protein A	P0AED2	16113	5.11	110	8.3E-07	79%	0.11	0.26	0.07	0.17	0.07	0.20	0.70	0.70	0.7	1.30	1.50	1.4	1.2
UspG	universal stress protein G	P39177	15925	6.03	101	1.8E-06	52%	0.07	0.11	0.08	0.045	0.07	0.05	-0.20	-0.10	-0.2	-0.90	-0.60	-0.8	0.6
SodA	superoxide dismutase [Mn]	P00448	22952	6.44	64	3.6E-02	44%	0.57	0.46	0.51	0.29	0.52	0.59	0.20	0.10	0.2	-0.80	0.20	-0.3	-0.3
PolA	DNA polymerase I	P00582	103168	5.4	119	2.4E-08	25%	0.046	0.034	0.07	0.06	0.08	0.045	-0.50	-0.70	-0.6	-0.10	-0.70	-0.4	-0.4
**Unclassified proteins (7 proteins)**							**Total RPM:**	**0.58**	**0.30**	**0.45**	**0.33**	**0.54**	**0.37**	0.4	0.1	**0.2**	-0.4	-0.5	**-0.5**	**-0.9**
GyrB	DNA gyrase subunit B	P0AES6	90153	5.68	135	2.7E-09	24%	0.15	0.043	0.14	0.06	0.15	0.06	0.10	0.00	0.1	-1.30	-1.20	-1.3	-1.8
MreB	regulator of FtsI, penicillin binding protein 3, septation function	Q8X9C9	37140	5.19	101	6.7E-06	34%	0.05	0.07	0.044	0.05	0.06	0.06	0.20	-0.30	-0.1	0.20	0.00	0.1	0.5
FtsZ	cell division protein FtsZ	P0A9A8	40299	4.65	138	1.3E-09	39%	0.045	0.026	0.06	0.06	0.06	0.047	-0.40	-0.30	-0.4	0.00	-0.30	-0.2	-0.8
RsmC	ribosomal RNA small subunit methyltransferase C	B1XFI0	37829	6	156	5.7E-12	51%	0.026	0.015	0.031	0.025	0.043	0.028	-0.20	-0.70	-0.5	-0.30	-0.60	-0.5	-0.7
Can	carbonic anhydrase	P61517	25366	6.16	89	9.8E-05	35%	0.15	0.09	0.14	0.10	0.19	0.09	0.10	-0.30	-0.1	-0.40	-1.10	-0.8	-0.8
FrsA	fermentation/respiration switch protein	Q8FKM5	47336	6.47	75	2.5E-03	24%	0.032	0.019	0.035	0.033	0.037	0.028	-0.10	-0.20	-0.2	-0.10	-0.40	-0.3	-0.8
YeaG	uncharacterized protein YeaG	P0ACY3	74776	5.63	66	5.2E-03	10%	0.12	0.041	0.00	0.00	0.00	0.048	>5	>5	>5	0.00	>5	-	-1.6
**Uncharacterized proteins (13 proteins)**							**Total RPM:**	**0.85**	**0.69**	**0.61**	**0.96**	**0.57**	**1.14**	0.5	0.6	**0.5**	0.7	1.0	**0.8**	**-0.3**
YhbW	uncharacterized protein YhbW	P0ADV5	37163	5.99	89	2.9E-05	33%	0.025	0.026	0.06	0.026	0.06	0.037	-1.30	-1.30	-1.3	-1.20	-0.70	-1.0	0.1
GalF	UTP-glucose-1-phosphate uridylyltransferase	P0AAB6	32979	5.73	61	1.9E-02	26%	0.019	0.018	0.033	0.044	0.033	0.028	-0.80	-0.80	-0.8	0.40	-0.20	0.1	0.0
YbeZ	putative ATP-binding protein in Pho regulon	Q0T6R9	39129	5.71	84	3.8E-04	41%	0.08	0.09	0.09	0.05	0.09	0.07	0.00	0.00	0.0	-0.70	-0.30	-0.5	0.0
YicC	UPF0701 protein YicC	P23839	33211	5.1	86	6.2E-05	43%	0.06	0.039	0.06	0.038	0.043	0.032	0.00	0.40	0.2	-0.60	-0.40	-0.5	-0.6

Table S1 - Quantitative data of individual proteins of *E. coli* BL21 (DE3) growing in defined and rich media

Name[1]	Protein name[1]	Uniprot ID[1]	Molecular mass[2] (Da)	Calculated pI value[2]	Mascot Score[2]	Mascot Expect Value[2]	Mascot Seq. Cover.[2]	Defined medium: DNB medium[7] exp. phase	Defined medium: DNB medium[7] stationary phase	Rich medium: TB medium[8] exp. phase	Rich medium: TB medium[8] stationary phase	Rich medium: LB medium[9] exp. phase	Rich medium: LB medium[9] stationary phase	Log$_2$ (DNB /TB) at exp. phase[4]	Log$_2$ (DNB /LB) at exp. phase[4]	**Log$_2$ (defined/ rich)[4] at exp. phase[4]**	Log$_2$(stationary phase /exp. phase)[5, 6] in TB medium	Log$_2$(stationary phase /exp. phase)[5, 6] in LB medium	**Log$_2$(stationary phase /exp. phase)[5, 6] in rich media[5]**	**Log$_2$(stationary phase /exp. phase)[5, 6] in defined medium[6]**
								Relative Protein Mass (RPM)[3] - %												
YajQ	UPF0234 protein YajQ	C4ZTI3	18333	5.96	178	3.6E-14	64%	0.035	0.042	0.041	0.08	0.06	0.049	-0.20	-0.80	-0.5	1.00	-0.30	0.4	0.3
YbgI	UPF0135 protein YbgI	P0AFP6	26990	5.07	89	2.7E-05	46%	0.05	0.044	0.044	0.033	0.038	0.05	0.30	0.50	0.4	-0.40	0.50	0.1	-0.3
YjgR	uncharacterized protein YjgR	P39342	54355	5.91	84	9.3E-05	28%	0.016	0.018	0.010	0.016	0.010	0.031	0.70	0.60	0.7	0.70	1.60	1.2	0.2
YdcJ	uncharacterized protein YdcJ	P76097	51353	5.35	58	3.6E-02	34%	0.021	0.042	0.040	0.11	0.00	0.07	-0.90	>5	-	1.50	>5	>2.5	1.0
YcgB	uncharacterized protein YcgB	P29013	61117	5.66	69	2.7E-03	15%	0.047	0.030	0.022	0.030	0.00	0.08	1.10	>5	>2.5	0.50	>5	>2.5	-0.7
YgaU	uncharacterized protein YgaU	P0ADE6	16053	5.71	70	2.1E-03	36%	0.12	0.07	0.026	0.09	0.06	0.29	2.30	1.00	1.7	1.80	2.20	2.0	-0.8
YfbU	hypothetical protein	P0A8W8	19638	6.06	111	6.6E-07	50%	0.13	0.13	0.06	0.12	0.07	0.09	1.00	0.90	1.0	0.90	0.40	0.7	0.0
LfiI	lateral flagellar export/assembly protein LfiI	B1LHP6	48085	6.53	77	1.6E-03	32%	0.16	0.07	0.08	0.25	0.08	0.23	1.00	0.90	1.0	1.70	1.50	1.6	-1.1
YhdH	putative quinone oxidoreductase YhdH	P26646	34873	5.63	111	1.8E-07	33%	0.08	0.07	0.050	0.07	0.024	0.07	0.70	1.70	1.2	0.40	1.60	1.0	-0.2

[1] Name, protein name and Uniprot ID are according to Uniprot database (http://www.uniprot.org/). Functional classifications are mostly according to EcoCyc database (http://ecocyc.org/). The classifications are confirmed by KEGG database (http://www.genome.jp/kegg/).

[2] Molecular mass, calculated pI, Mascot score, expect and sequence coverage are from MASCOT search program (Matrix Science, UK, http://www.matrixscience.com/), based on the annotated *E. coli* genome [Uniprot (http://www.uniprot.org/) serving as database].

[3] Relative Protein Mass (RPM) - %: Each spot's intensity was normalized by the whole spots intensity on each 2D gel. The corresponding average from duplicate gels was used indicating each spot's protein portion (%) of Relative Protein Mass (RPM). The RPM (%) of all spots representing the same protein was used for indicating the abundance of the corresponding protein. For each protein's RPM (%), number with two digits after the decimal point is given. But for a better comparison of low abundance proteins (<0.05 %), three digits after the decimal point are given.

[4] Log$_2$(defined/rich) at exp. phase: Average of Log$_2$[DNB (medium)/TB (medium)] and Log$_2$[DNB (medium)/LB (medium)] at exponential phase

[5] Log$_2$(stationary phase/exp. phase) in rich media: Average of Log$_2$(stationary phase/exponential phase) in TB and LB media

[6] Log$_2$(stationary phase/exp. phase) in defined medium: Log$_2$(stationary phase/exponential phase) in DNB medium

To facilitate the comparison, the color code for Log$_2$ ratio is indicated as follows:

Log$_2$ ratio: -2 -1.5 -1 -0.5 0 0.5 1 1.5 2

[7] DNB medium: 10.91 g L^{-1} glucose, 4 g L^{-1} $(NH_4)_2HPO_4$, 13.3 g L^{-1} KH_2PO_4, 1.554 g L^{-1} Citric acid, 0.586 g L^{-1} $MgSO_4$, 0.1008 g L^{-1} Fe(III) citrate, 2.1 mg L^{-1} $Na_2MoO_4.2H_2O$, 2.5 mg L^{-1} $CoCl_2.6H_2O$, 15 mg L^{-1} $MnCl_2.4H_2O$, 1.5 mg L^{-1} $CuCl_2.2H_2O$, 3 mg L^{-1} H_3BO_3, 33.8 mg L^{-1} $Zn(CH_3COOH)_2.2H_2O$, 14.10 mg L^{-1} Titriplex III. μ_{max}= 0.8 h^{-1}

[8] TB medium: 12 g L^{-1} tryptone, 24 g L^{-1} yeast extract, 5 g L^{-1} glycerol, 2.31 g L^{-1} KH_2PO_4, 12.54 g L^{-1} K_2HPO_4. μ_{max}= 1.4 h^{-1}

[9] LB medium: 10 g L^{-1} tryptone, 5 g L^{-1} yeast extract, 5 g L^{-1} NaCl. μ_{max}= 1.6 h^{-1}

[10] LpdA is assigned to both groups, pyruvate decarboxylation to acetyl CoA and TCA cycle.

[11] A truncated outer membrane protein A is identified in the NCBI database (http://www.ncbi.nlm.nih.gov/).

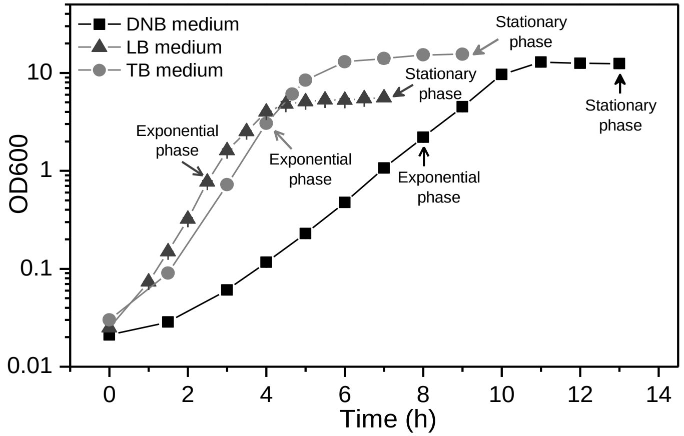

***E. coli* growth and sampling time for 2D gel electrophoresis analysis**
Cultivations were carried out at 37 ℃ in 2 L shake flasks (200 mL working volume) using DNB (black square), LB (dark gray triangle) and TB (light gray circle) media. Samples for 2D gel electrophoresis analysis were taken at the time points indicated by arrows: exponential phase (open arrow) and stationary phase (stealth arrow).

Table S1.1 - Summary of quantitative data of individual proteins of *E. coli* BL21 (DE3) growing in defined and rich media*

Cat. Nr.	Nr.	Category and group[1]	Defined medium		Rich medium				Log$_2$ (DNB /TB)	Log$_2$ (DNB /LB)	**Log$_2$ (defined/ rich)[3]**	**Log$_2$(stationary phase /exp. phase)[4, 5]**			
			DNB medium[6]		TB medium[7]		LB medium[8]								
			exp. phase	stationary phase	exp. phase	stationary phase	exp. phase	stationary phase				in TB medium	in LB medium	**in rich media[4]**	**in defined medium[5]**
			Relative Protein Mass (RPM)[2] - %						**at exp. phase[3]**						
I		Carbohydrate Metabolism (51 proteins) Sum 1 - 4[9]	16.46	17.24	16.06	24.79	17.52	21.82	0.0	-0.1	0.0	0.6	0.3	0.5	0.1
	1	Central carbon metabolism (24 proteins) Sum 1.1 - 1.5	7.05	9.29	10.07	14.06	9.96	9.34	-0.5	-0.5	-0.5	0.5	-0.1	0.2	0.4
	1.1	Sub-group: Upper glycolysis (5 proteins)	1.38	1.79	1.24	2.14	1.20	1.53	0.2	0.2	0.2	0.8	0.3	0.6	0.4
	1.2	Sub-group: Lower glycolysis (7 proteins)	2.47	3.35	3.20	4.56	3.31	3.63	-0.4	-0.4	-0.4	0.5	0.1	0.3	0.4
	1.3	Sub-group: Pyruvate decarboxylation to acetyl CoA (4 proteins)[9]	2.01	2.80	4.16	4.77	3.91	2.16	-1.1	-1.0	-1.0	0.2	-0.9	-0.3	0.5
	1.4	Sub-group: Glycerol metabolism (2 proteins)	0.10	0.19	0.16	1.05	0.18	0.38	-0.7	-0.9	-0.8	2.7	1.1	1.9	1.0
	1.5	Sub-group: Pentose phosphate pathway (PPP) (6 proteins)	1.10	1.17	1.32	1.54	1.35	1.64	-0.3	-0.3	-0.3	0.2	0.3	0.3	0.1
	2	By-product metabolism (9 proteins)	1.53	1.26	1.95	1.56	2.87	2.22	-0.3	-0.9	-0.6	-0.3	-0.4	-0.3	-0.3
	3	TCA cycle (15 proteins)[9]	7.98	6.77	4.56	9.99	5.28	10.44	0.8	0.6	0.7	1.1	1.0	1.1	-0.2
	4	Anaplerotic reactions (4 proteins)	0.70	0.80	0.53	0.84	0.39	0.79	0.4	0.9	0.6	0.7	1.0	0.8	0.2
II	5	Oxidative phosphorylation (7 proteins)	2.68	2.52	2.31	2.69	2.87	2.42	0.2	-0.1	0.1	0.2	-0.2	0.0	-0.1
III		Synthesis of biomass building blocks (121 proteins) Sum 6 - 10	25.44	24.78	13.67	14.27	13.41	15.66	0.9	0.9	0.9	0.1	0.2	0.1	0.0
	6	Amino acid biosynthesis and metabolism (68 proteins) Sum 6.1 - 6.2	19.13	18.69	6.16	8.45	6.32	10.09	1.6	1.6	1.6	0.5	0.7	0.6	0.0
	6.1	Sub-group: Amino acid biosynthesis (57 proteins)	18.18	17.67	5.42	6.29	5.41	6.85	1.7	1.7	1.7	0.2	0.3	0.3	0.0
	6.2	Sub-group: Amino acid degradation (11 proteins)	0.95	1.02	0.74	2.17	0.91	3.24	0.4	0.1	0.2	1.5	1.8	1.7	0.1
	7.1	IMP biosynthesis (for nucleotide) (7 proteins)	1.28	1.39	0.43	0.47	0.39	0.61	1.6	1.7	1.6	0.1	0.6	0.4	0.1
	7.2	Nucleotide biosynthesis (start from IMP) (13 proteins)	1.78	1.77	2.92	2.01	2.59	1.92	-0.7	-0.5	-0.6	-0.5	-0.4	-0.5	0.0
	8	Fatty acid biosynthesis (7 proteins)	1.28	1.05	1.81	1.12	2.18	1.15	-0.5	-0.8	-0.6	-0.7	-0.9	-0.8	-0.3
	9	Lipopolysaccharide biosynthesis (10 proteins)	0.90	0.89	1.28	1.24	0.99	0.91	-0.5	-0.1	-0.3	0.0	-0.1	-0.1	0.0
	10	Synthesis of other cellular components (16 proteins)	1.08	0.98	1.07	0.97	0.94	0.98	0.0	0.2	0.1	-0.1	0.1	0.0	-0.1
IV	11	(Metabolite) degradation (19 proteins)	1.46	1.41	1.35	3.92	1.32	2.63	0.1	0.1	0.1	1.5	1.0	1.3	-0.1
V		Transportation (28 proteins) Sum 12 - 14	6.60	10.17	6.27	6.44	6.82	7.75	0.1	0.0	0.0	0.0	0.2	0.1	0.6
	12	Sugar transport (7 proteins)	1.07	1.64	0.94	1.36	0.84	1.38	0.2	0.4	0.3	0.5	0.7	0.6	0.6
	13	Amino acid and peptide transport (7 proteins)	0.76	1.28	0.38	0.59	0.29	1.12	1.0	1.4	1.2	0.6	1.9	1.3	0.7
	14	Other transport proteins (14 proteins)	4.76	7.26	4.95	4.49	5.69	5.24	-0.1	-0.3	-0.2	-0.1	-0.1	-0.1	0.6
VI		Transcription and translation (66 proteins) Sum 15 - 22	17.85	17.05	26.72	18.67	28.19	16.51	-0.6	-0.7	-0.6	-0.5	-0.8	-0.6	-0.1
	15	RNA polymerases (3 proteins)	1.18	1.01	1.48	0.74	1.72	0.71	-0.3	-0.5	-0.4	-1.0	-1.3	-1.1	-0.2
	16	RNA polymerase binding proteins (7 proteins)	0.94	0.87	1.40	0.95	1.55	0.92	-0.6	-0.7	-0.7	-0.6	-0.7	-0.7	-0.1
	17	Transcription factors (11 proteins)	1.77	1.66	1.64	1.65	1.92	2.07	0.1	-0.1	0.0	0.0	0.1	0.1	-0.1

Table S1.1 - Summary of quantitative data of individual proteins of *E. coli* BL21 (DE3) growing in defined and rich media*

Cat. Nr.	Nr.	Category and group[1]	Defined medium		Rich medium				Log_2 (DNB /TB)	Log_2 (DNB /LB)	**Log_2 (defined/ rich)[3]**	**Log_2(stationary phase /exp. phase)[4, 5]**			
			DNB medium[6]		TB medium[7]		LB medium[8]								
			exp. phase	stationary phase	exp. phase	stationary phase	exp. phase	stationary phase				in TB medium	in LB medium	**in rich media[4]**	**in defined medium[5]**
			Relative Protein Mass (RPM)[2] - %						**at exp. phase[3]**						
	18	Ribosomal proteins (5 proteins)	2.98	2.72	5.25	2.39	5.99	2.27	-0.8	-1.0	-0.9	-1.1	-1.4	-1.3	-0.1
	19	Ribosome-associated proteins (8 proteins)	0.90	0.98	1.64	1.00	1.68	1.00	-0.9	-0.9	-0.9	-0.7	-0.8	-0.7	0.1
	20	Aminoacyl-tRNA synthetases (23 proteins)	3.57	2.86	5.28	4.58	4.97	3.32	-0.6	-0.5	-0.5	-0.2	-0.6	-0.4	-0.3
	21	Elongation factors (6 proteins)	6.00	6.61	9.25	7.02	9.68	5.83	-0.6	-0.7	-0.7	-0.4	-0.7	-0.6	0.1
	22	RNA degradation (3 proteins)	0.52	0.34	0.77	0.34	0.69	0.39	-0.6	-0.4	-0.5	-1.2	-0.8	-1.0	-0.6
VII		Protein folding and degradation (28 proteins) Sum 23 - 25	4.61	4.83	6.16	7.02	5.97	6.50	-0.4	-0.4	-0.4	0.2	0.1	0.2	0.1
	23	Isomerases (5 proteins)	0.55	0.63	0.67	0.63	0.52	0.70	-0.3	0.1	-0.1	-0.1	0.4	0.2	0.2
	24	Chaperones (9 proteins)	2.83	3.11	3.94	4.48	4.08	4.06	-0.5	-0.5	-0.5	0.2	0.0	0.1	0.1
	25	Proteases (13 proteins)	1.23	1.10	1.55	1.91	1.37	1.75	-0.3	-0.2	-0.2	0.3	0.4	0.3	-0.2
VIII		Cell redox balance (22 proteins) Sum 26.1 - 26.3	2.88	3.64	2.82	5.06	2.54	3.97	0.0	0.2	0.1	0.8	0.6	0.7	0.3
	26.1	Other dehydrogenases (9 proteins)	0.93	0.78	0.79	0.79	0.76	1.02	0.2	0.3	0.3	0.0	0.4	0.2	-0.2
	26.2	Oxidoreductases (8 proteins)	0.51	0.68	0.48	0.82	0.49	0.90	0.1	0.0	0.1	0.8	0.9	0.8	0.4
	26.3	Hydroperoxide reductases and superoxide dismutase (5 proteins)	1.44	2.19	1.55	3.45	1.29	2.05	-0.1	0.2	0.0	1.2	0.7	0.9	0.6
IX	27	DNA protection and repair (7 proteins)	0.83	1.39	0.76	1.22	0.86	1.74	0.1	-0.1	0.0	0.7	1.0	0.9	0.8
X	28	Unclassified proteins (7 proteins)	0.58	0.30	0.45	0.33	0.54	0.37	0.4	0.1	0.2	-0.5	-0.6	-0.5	-0.9
XI	29	Uncharacterized proteins (13 proteins)	0.85	0.69	0.61	0.96	0.57	1.14	0.5	0.6	0.5	0.7	1.0	0.8	-0.3

* Supplementary Table 1.1 is a summary of proteome data for all categories and groups from Supplementary Table 1

1 Category and group: functional classifications are mostly according to EcoCyc database (http://ecocyc.org/). The classifications are confirmed by KEGG database (http://www.genome.jp/kegg/).

2 Relative Protein Mass (RPM) - %: Each spot's intensity was normalized by the whole spots intensity on each 2D gel. The corresponding average from duplicate gels was used indicating each spot's protein portion (%) of relative protein mass (RPM). The total "RPM %" of all spots representing the same protein was used for indicating the abundance of the corresponding protein. The total "RPM %" of all proteins belonging to the same category or group was used for indicating the abundance of the corresponding category or group.

3 Log_2(defined/rich) at exp. phase: Average of Log_2[DNB (medium)/TB (medium)] and Log_2[DNB (medium)/LB (medium)] at exponential phase

4 Log_2(stationary phase/exp. phase) in rich media: Average of Log_2(stationary phase/exponential phase) in TB and LB media

5 Log_2(stationary phase/exp. phase) in defined medium: Log_2(stationary phase/exponential phase) in DNB medium

To facilitate the comparison, the color code for Log_2 ratio is indicated as follows: **Log_2 ratio:** -2 -1.5 -1 -0.5 0 0.5 1 1.5 2

6 DNB medium: 10.91 g L^{-1} glucose, 4 g L^{-1} $(NH_4)_2HPO_4$, 13.3 g L^{-1} KH_2PO_4, 1.554 g L^{-1} Citric acid, 0.586 g L^{-1} $MgSO_4$, 0.1008 g L^{-1} Fe(III) citrate, 2.1 mg L^{-1} $Na_2MoO_4.2H_2O$, 2.5 mg L^{-1} $CoCl_2.6H_2O$, 15 mg L^{-1} $MnCl_2.4H_2O$, 1.5 mg L^{-1} $CuCl_2.2H_2O$, 3 mg L^{-1} H_3BO_3, 33.8 mg L^{-1} $Zn(CH_3COOH)_2.2H_2O$, 14.10 mg L^{-1} Titriplex III. μ_{max}= 0.8 h^{-1}

7 TB medium: 12 g L^{-1} tryptone, 24 g L^{-1} yeast extract, 5 g L^{-1} glycerol, 2.31 g L^{-1} KH_2PO_4, 12.54 g L^{-1} K_2HPO_4. μ_{max}= 1.4 h^{-1}

8 LB medium: 10 g L^{-1} tryptone, 5 g L^{-1} yeast extract, 5 g L^{-1} NaCl. μ_{max}= 1.6 h^{-1}

9 LpdA is assigned to both groups, pyruvate decarboxylation to acetyl CoA and TCA cycle.

Table S2 - Transcriptional control of glycolysis and TCA cycle genes

Pathway	Gene	Detected in this study?	Promoter[1]	Sigma Factor[1, 2]	Transcription factor[1, 3]	Function[1]	Binding sites[1]	Binding sequence[1, 3]
Upper glycolysis	*pgi*	No	*pgip*	**RpoS**, RpoD	SoxS	activator	1	cattacgctaACGGCACTAAAACCATCACAtttttctgtg
	pfkA	Yes	*pfkAp2*	**RpoS**, RpoD	Cra (FruR)	repressor	1	atttggcctgACCTGAATCAATTCAGCAggaagtgatt
			pfkAp1	**RpoS**, RpoD				
	pfkB	Yes	*pfkBp2*	**RpoS**				
			pfkBp1	**RpoS**				
	fbaA	Yes	*epdp*	**RpoS**, RpoD	**CRP-cAMP**	activator	1	aagaagacatTTATCTGACTCACATCACACTTttatcccctt
					**Cra (FruR)**	repressor	1	acatttaatcGACTGAAACGCTTCAGCTaggataagcg
			pgkp13	RpoD				
			pgkp2					
	fbaB	Yes	*fbaBp*	**RpoS**	**Cra (FruR)**	repressor	1	cagcgttttcTGTTGGCTCGATTCATCAgaaaaaatgt
	tpiA	Yes	*tpiAp2*	**RpoS**, RpoD	**Cra (FruR)**	repressor	1	agatagcgccAGCTTAATCGGTTCAACAgcgaaggtca
			tpiAp1					
Lower glycolysis	*gapA*	Yes	*gapAp3*	RpoD	**CRP-cAMP**	activator	1	gctgcacctaAATCGTGATGAAAATCACATTTttatcgtaat
			gapAp1	**RpoS**, RpoD	Cra (FruR)	repressor		
			gapAp2	**RpoS**				
			gapAp4	RpoD				
	pgk	Yes	*epdp*	**RpoS**, RpoD	**CRP-cAMP**	activator	1	aagaagacatTTATCTGACTCACATCACACTTttatcccctt
					**Cra (FruR)**	repressor	1	acatttaatcGACTGAAACGCTTCAGCTaggataagcg
			pgkp13	RpoD				
			pgkp2					
	gpmA	Yes	*gpmAp*	**RpoS**, RpoD	Fur	repressor	2	atcatcttttAATGATAATAATTCTCATTatattgccgc<br>caaatcatctTTTAATGATAATAATTCTCattatattgc
			pmAp2	**RpoS**, RpoD				
	gpmM	No	*gpmMp*		**Cra (FruR)**	repressor	1	cttcagaggcTATTTTATCGATTCAGCTgtagtaaaat
			gpmMp2	**RpoS**				
	eno	Yes	*enop123*		Cra (FruR)	repressor		
			enop47	**RpoS**, RpoD				
			enop6					
			pyrGp					
			pyrGp2					
			pyrGp1					
	pykA	Yes	*pykAp12*	**RpoS**, RpoD				
	pykF	Yes	*pykFp*	RpoD	**Cra (FruR)**	repressor	1	gccaattgacTCTTGAATGGTTTCAGCActttggactg
			pykFp12	**RpoS**, RpoD				
			pykFp3					
	ppsA	Yes	*ppsp*	RpoD	**Cra (FruR)**	repressor	1	tgaaaaaaacGGTGAATCGTTCAAGCaaatatattt

Table S2 - Transcriptional control of glycolysis and TCA cycle genes

Pathway	Gene	Detected in this study?	Promoter[1]	Sigma Factor[1, 2]	Transcription factor[1, 3]	Function[1]	Binding sites[1]	Binding sequence[1, 3]
Pyruvate dehydrogenase complex	*aceEF*	Yes	*pdhRp*	**RpoS**, RpoD	**CRP-cAMP**	activator	6	atgtgcacagTTTCATGATTTCAATCAAAACCtgtatggaca<br>caagttgttaAAATGTGCACAGTTTCATGATTtcaatcaaaa<br>taaagtctacATTTGTGCATAGTTACAACTTTgaaacgttat<br>tacatcaaagAAGTTTGAATTGTTACAAAAAGacttccgtca<br>ctttgaaacgTTATATATGTCAAGTTGTTAAAatgtgcacag<br>atatatgtcaAGTTGTTAAAATGTGCACAGTTtcatgatttc
					**Cra (FruR)**	repressor	1	caaaattggtAAGTGAATCGGTTCAATTcggattttta
					PdhR	repressor	1	cacagacatgAAATTGGTAAGACCAATtgacttcggc
					FNR	dual	1	gcacagtttcATGATTTCAATCAAaacctgtatg
			aceEp	**RpoS**, RpoD	ArcA-Phosphorylated	repressor		
					FNR	repressor		
					NsrR-nitric oxide	repressor	1	aattatccagAAGATGTTGTAaatcaagcgc
	lpdA	Yes	*pdhRp*	**RpoS**, RpoD	**CRP-cAMP**	activator	6	atgtgcacagTTTCATGATTTCAATCAAAACCtgtatggaca<br>caagttgttaAAATGTGCACAGTTTCATGATTtcaatcaaaa<br>taaagtctacATTTGTGCATAGTTACAACTTTgaaacgttat<br>tacatcaaagAAGTTTGAATTGTTACAAAAAGacttccgtca<br>ctttgaaacgTTATATATGTCAAGTTGTTAAAatgtgcacag<br>atatatgtcaAGTTGTTAAAATGTGCACAGTTtcatgatttc
					**Cra (FruR)**	repressor	1	caaaattggtAAGTGAATCGGTTCAATTcggattttta
					PdhR	repressor	1	cacagacatgAAATTGGTAAGACCAATtgacttcggc
					FNR	dual	1	gcacagtttcATGATTTCAATCAAaacctgtatg
			lpdAp	RpoD	**ArcA-Phosphorylated**	repressor	2	tttaaaaattGTTAACAATTTTGTAaaataccgac<br>cgtttgttgtTTAAAAATTGTTAACaattttgtaa
					**CRP-cAMP**	repressor ?	1	gttgtttaaaAATTGTTAACAATTTTGTAAAAtaccgacgga
					FNR	dual		
					Fis	activator	1	gccgtttgttGTTTAAAAATTGTTAacaattttgt
					Fur	dual		
TCA cycle	*gltA*	Yes	*gltAp1*	RpoD	**ArcA-Phosphorylated**	repressor	1	gtaatgtttgTAACAACTTTGTTGAatgattgtca
					**CRP-cAMP**	activator	1	taaatgttgtTATCGTGACCTGGATCACTGTTcaggataaaa
					IHF	activator	1	aatgattgtcAAATTAGATGATTaaaaattaaa
			gltAp2					
	acnA	Yes	*acnAp2*	RpoD	**ArcA-Phosphorylated**	repressor	1	aatttgggttGTTATCAAATCGTTAcgcgatgttt
					**CRP-cAMP**	activator	1	ctcttttatcAATTTGGGTTGTTATCAAATCGttacgcgatg
					FNR	repressor	1	tttatcaattTGGGTTGTTATCAAatcgttacgc
					Cra (FruR)	activator		
					MarA	activator	1	aaggtttctcCTCTTTTATCAATTTGGGTTgttatcaaat
					Rob	activator	1	aaggtttctcCTCTTTTATCAATTTGGGTTgttatcaaat
					SoxS	activator	1	aaggtttctcCTCTTTTATCAATTTGGGTTgttatcaaat
			acnAp1	**RpoS**, RpoD				

Table S2 - Transcriptional control of glycolysis and TCA cycle genes

Pathway	Gene	Detected in this study?	Promoter[1]	Sigma Factor[1, 2]	Transcription factor[1, 3]	Function[1]	Binding sites[1]	Binding sequence[1, 3]
TCA cycle	*acnB*	Yes	*acnBp*	RpoD	**ArcA-Phosphorylated**	repressor	7	gaggcggtagTTTAAATTTTGACTAatcttgggat
								cttgggattcGTTGAGAAAGGTGATtatcaccatg
								ggattcgttgAGAAAGGTGATTATCaccatgcgaa
								ccatgcgaatTAACGAAGTTTTTACggagggaaac
								ttatcaccatGCGAATTAACGAAGTttttacggag
								gattcagccaCTTTTTTATGTTGCTtttttgtaaa
								acttttttatGTTGCTTTTTTGTAAacagattaac
					**CRP-cAMP**	activator	1	acttttttatGTTGCTTTTTTGTAAacagattaac
					Fis	repressor	3	aagaggcggtAGTTTAAATTTTGACtaatcttggg
								tctagaccatCCTTAACGATTCAGCcactttttta
								tttgtaaacaGATTAACACCTCGTCaaaatcctgc
					Cra (FruR)	repressor		
			acnBp2					
	icd	Yes	*icdAp1*	RpoD	**ArcA-Phosphorylated**	repressor	2	gggtagtattGACAAGCCAATTACAaatcattaac
								attgacaagcCAATTACAAATCATTaacaaaaaat
			icdAp2	RpoD	**Cra (FruR)**	repressor	1	gaacgttgcgAGCTGAATCGCTTAACCTggtgatttct
	sucABCD	Yes	*sdhCp*	RpoD	**ArcA-Phosphorylated**	repressor	5	ttgtaacaacTTTGTTGAATGATTGtcaaattaga
								tgttacataaGTTAATCTTAGGTGAaataccgact
								acaaagacctGTTAATCTGGACCTAcagaccatcc
								atgtaggttaATTGTAATGATTTTGtgaacagcct
								ggttaattgtAATGATTTTGTGAACagcctatact
					**CRP-cAMP**	activator	1	taaatgttgtTATCGTGACCTGGATCACTGTTcaggataaaa
					FNR	repressor		
					Fur	activator	1	ctatatgtagGTTAATTGTAATGATTTTGtgaacagcct
			sucAp	**RpoS**	ArcA-Phosphorylated	repressor		
					FNR	repressor		
					IHF	repressor		
	sdhAB	Yes	*sdhCp*	RpoD	**ArcA-Phosphorylated**	repressor	5	ttgtaacaacTTTGTTGAATGATTGtcaaattaga
	sdhCD	No						tgttacataaGTTAATCTTAGGTGAaataccgact
								acaaagacctGTTAATCTGGACCTAcagaccatcc
								atgtaggttaATTGTAATGATTTTGtgaacagcct
								ggttaattgtAATGATTTTGTGAACagcctatact
					**CRP-cAMP**	activator	1	taaatgttgtTATCGTGACCTGGATCACTGTTcaggataaaa
					FNR	repressor		
					Fur	activator	1	ctatatgtagGTTAATTGTAATGATTTTGtgaacagcct
			sdhDp2	RpoD	**CRP-cAMP**	activator	1	tgggcagcttCTTCGTCAAATTTATCATGTGGggcatcctta
	fumA	Yes	*fumAp*	RpoD	ArcA-Phosphorylated	repressor		
					**CRP-cAMP**	activator	1	ttcctgttcaAAGTATTATGCGCAGCACAGCCactctccatt
					FNR	repressor		
	fumB	No	*fumBp*	RpoD	ArcA-Phosphorylated	activator		

Table S2 - Transcriptional control of glycolysis and TCA cycle genes

Pathway	Gene	Detected in this study?	Promoter[1]	Sigma Factor[1, 2]	Transcription factor[1, 3]	Function[1]	Binding sites[1]	Binding sequence[1, 3]
TCA cycle					FNR	activator	3	gcacgatacgCTCACACCAATCAAccccggcaga<br>gcacgcaaagTGCATTTATAAGAAcccgtacatc<br>gcaaagtgcaTTTATAAGAACCCGtacatcgcgg
					Fis	repressor		
					Fur	activator		
			dcuBp		**CRP-cAMP**	activator	1	tcgttaccggCTTTAGCAAATACCTCACAGTGaatattggct
					FNR	activator	1	cagtcacgttCTGTTTTGTATGAActgttttcag
					NarL-Phosphorylated	repressor	6	tggatagtaaATAACATgtgtgaaccc<br>tatcagtattATGATAAgttggatagt<br>agtaaataacATGTGTGaaccctcgcg<br>aaccctcgcgATAATCCtatttaaatt<br>tcacgttctgTTTTGTAtgaactgttt<br>ctttagcaaaTACCTCAcagtgaatat
			dcuBp2		DcuR-Phosphorylated	activator	2	ggtgacataaTAGTTAATTAACTTTTGttagcgtttt<br>ttttgttagcGTTTTGAAATTAAAAACaccgttcacc
	fumC	Yes	*fumCp*		ArcA-Phosphorylated	repressor		
					FNR	repressor		
					Fur	repressor		
					MarA	activator	1	ttcacacagcGGGTGCATTGTGTGAGTTGTAtctgctggaa
					Rob	activator		
					SoxR	activator		
					SoxS	activator	1	ataacaaatgTTTGGTCTTTCGTGCCATgtaaaaaaac
	mdh	Yes	*mdhp1*	RpoD	**ArcA-Phosphorylated**	repressor	1	ctaaactcctTATTATATTGATAAActaagatatg
					**CRP-cAMP**	activator	1	ccacatctcaAGAATGTGTAGTCACGCAAGTTtagcgtttat
					DpiA-P^{asp}	activator		
					FlhDC	repressor		
			mdhp2		DpiA-P^{asp}	activator		
	glcB	Yes	*glcDp*	RpoD	**ArcA-Phosphorylated**	repressor	2	tcttggttaaCTCAATGTTAAATTGatgtaacata<br>taactcaatgTTAAATTGATGTAACataatcactt
					GlcC-Glycolate	activator	1	cagaaaaattGGTCCTACCTGTGCAcgaggtccgg
					IHF	activator	1	ggttaactcaATGTTAAATTGATgtaacataat
			glcBp					

[1] The information was extracted from RegulonDB Version 7.0 (http://regulondb.ccg.unam.mx) [1]. (The database will be updated on a regular basis)

[2] Sigma factor σ^S (RpoS) is written in bold letters for better recognition.

[3] Cra (FruR), CRP-cAMP, and ArcA-Phosphorylated (ArcA-P) are written in bold letters for better recognition (only included when predicted binding site was found).

[1] Gama-Castro S, et al., RegulonDB version 7.0: transcriptional regulation of *Escherichia coli* K-12 integrated within genetic sensory response units (Gensor Units). *Nucleic Acids Res* 2011, 39:D98-105.

Table S3 - DksA-ppGpp controlled genes of *E. coli*

Category[1]	Gene[1, 2]	Function[2]	Detected in this study?	Biological function[1]
Transcription and translation	*rpoA*	inhibition	Yes	RNA polymerase
	rpoB	inhibition	Yes	RNA polymerase
	rpoC	inhibition	Yes	RNA polymerase
	rpoZ	inhibition	No	RNA polymerase
	rpoD	inhibition	Yes	Sigma 70 factor
	rplA	inhibition	No	Ribosomal protein
	rplB	inhibition	No	Ribosomal protein
	rplC	inhibition	No	Ribosomal protein
	rplD	inhibition	No	Ribosomal protein
	rplE	inhibition	No	Ribosomal protein
	rplF	inhibition	No	Ribosomal protein
	rplJ	inhibition	No	Ribosomal protein
	rplK	inhibition	No	Ribosomal protein
	rplL	inhibition	Yes	Ribosomal protein
	rplN	inhibition	No	Ribosomal protein
	rplO	inhibition	No	Ribosomal protein
	rplP	inhibition	No	Ribosomal protein
	rplQ	inhibition	No	Ribosomal protein
	rplR	inhibition	No	Ribosomal protein
	rplS	inhibition	No	Ribosomal protein
	rplT	inhibition	No	Ribosomal protein
	rplV	inhibition	No	Ribosomal protein
	rplW	inhibition	No	Ribosomal protein
	rplX	inhibition	No	Ribosomal protein
	rpmC	inhibition	No	Ribosomal protein
	rpmD	inhibition	No	Ribosomal protein
	rpmI	inhibition	No	Ribosomal protein
	rpmJ	inhibition	No	Ribosomal protein
	rpsA	inhibition	Yes	Ribosomal protein
	rpsC	inhibition	No	Ribosomal protein
	rpsD	inhibition	No	Ribosomal protein
	rpsE	inhibition	No	Ribosomal protein
	rpsG	inhibition	No	Ribosomal protein
	rpsH	inhibition	No	Ribosomal protein
	rpsJ	inhibition	No	Ribosomal protein
	rpsK	inhibition	No	Ribosomal protein
	rpsL	inhibition	No	Ribosomal protein
	rpsM	inhibition	No	Ribosomal protein
	rpsN	inhibition	No	Ribosomal protein
	rpsP	inhibition	No	Ribosomal protein
	rpsQ	inhibition	No	Ribosomal protein
	rpsS	inhibition	No	Ribosomal protein
	rpsT	inhibition	No	Ribosomal protein
	rpsU	inhibition	No	Ribosomal protein
	rimM	inhibition	No	Ribosome maturation protein
	rrfA	inhibition	No	Ribosomal RNA
	rrfB	inhibition	No	Ribosomal RNA
	rrfC	inhibition	No	Ribosomal RNA
	rrfD	inhibition	No	Ribosomal RNA
	rrfE	inhibition	No	Ribosomal RNA
	rrfF	inhibition	No	Ribosomal RNA
	rrfG	inhibition	No	Ribosomal RNA
	rrfH	inhibition	No	Ribosomal RNA
	rrlA	inhibition	No	Ribosomal RNA

Table S3 - DksA-ppGpp controlled genes of *E. coli*

Category[1]	Gene[1, 2]	Function[2]	Detected in this study?	Biological function[1]
Transcription and translation	*rrlB*	inhibition	No	Ribosomal RNA
	rrlC	inhibition	No	Ribosomal RNA
	rrlD	inhibition	No	Ribosomal RNA
	rrlE	inhibition	No	Ribosomal RNA
	rrlG	inhibition	No	Ribosomal RNA
	rrlH	inhibition	No	Ribosomal RNA
	rrsA	inhibition	No	Ribosomal RNA
	rrsB	inhibition	No	Ribosomal RNA
	rrsC	inhibition	No	Ribosomal RNA
	rrsD	inhibition	No	Ribosomal RNA
	rrsE	inhibition	No	Ribosomal RNA
	rrsG	inhibition	No	Ribosomal RNA
	rrsH	inhibition	No	Ribosomal RNA
	alaT	inhibition	No	tRNA
	alaU	inhibition	No	tRNA
	alaV	inhibition	No	tRNA
	gltT	inhibition	No	tRNA
	gltU	inhibition	No	tRNA
	gltV	inhibition	No	tRNA
	gltW	inhibition	No	tRNA
	ileT	inhibition	No	tRNA
	ileU	inhibition	No	tRNA
	ileV	inhibition	No	tRNA
	thrV	inhibition	No	tRNA
	dusB	inhibition	No	tRNA dihydrouridine synthesis
	trmD	inhibition	No	tRNA methylation
	trmH	inhibition	No	tRNA methylation
	gluQ	inhibition	No	tRNA aminoacylation
	pcnB	inhibition	No	mRNA polyadenylation
	pheS	inhibition	No	Aminoacyl-tRNA synthetases
	pheT	inhibition	Yes	Aminoacyl-tRNA synthetases
	thrS	inhibition	Yes	Aminoacyl-tRNA synthetases
	fusA	inhibition	Yes	Elongation factor
	tufA	inhibition	Yes	Elongation factor
	infA	inhibition	No	Protein chain initiation factor
	infC	inhibition	No	Protein chain initiation factor
	pheM	inhibition	No	Regulation of transcription
	fis	inhibition	No	Transcriptional regulator
	ihfB	inhibition	No	Transcriptional regulator
	dksA	inhibition	Yes	Transcriptional regulator
	spoT	inhibition	No	ppGpp biosynthesis
Amino acid biosynthesis	*argI*	activation	Yes	Amino acid biosynthesis
	hisA	activation	No	Amino acid biosynthesis
	hisB	activation	No	Amino acid biosynthesis
	hisC	activation	Yes	Amino acid biosynthesis
	hisD	activation	Yes	Amino acid biosynthesis
	hisF	activation	Yes	Amino acid biosynthesis
	hisG	activation	Yes	Amino acid biosynthesis
	hisH	activation	No	Amino acid biosynthesis
	hisI	activation	No	Amino acid biosynthesis
	hisL	activation	No	Amino acid biosynthesis
	thrA	activation	No	Amino acid biosynthesis
	thrB	activation	Yes	Amino acid biosynthesis

Table S3 - DksA-ppGpp controlled genes of *E. coli*

Category[1]	Gene[1, 2]	Function[2]	Detected in this study?	Biological function[1]
Amino acid biosynthesis	*thrC*	activation	No	Amino acid biosynthesis
	thrL	activation	No	Amino acid biosynthesis
Others	*livJ*	activation	Yes	Branched chain amino acid ABC transporter
	recG	inhibition	No	Response to DNA damage stimulus
	secY	inhibition	No	Intracellular protein transmembrane transport
	folk	inhibition	No	Tetrahydrofolate and folic acid biosynthetic process
	dnaG	inhibition	No	DNA replication, synthesis of RNA primer

[1] The information, nomenclature and classification are according to EcoCyc database [1] (http://ecocyc.org/).

[2] The information was extracted from RegulonDB Version 7.0 (http://regulondb.ccg.unam.mx) [2]. (The database will be updated on a regular basis)

[1] Keseler, I.M., et al., EcoCyc: a comprehensive database of *Escherichia coli* biology. Nucleic Acids Res, 2011. 39(Database issue): p. D583-90.

[2] Gama-Castro S, et al., RegulonDB version 7.0: transcriptional regulation of *Escherichia coli* K-12 integrated within genetic sensory response units (Gensor Units). *Nucleic Acids Res* 2011, 39:D98-105.

Table S4 - RpoS controlled genes of *E. coli*

Category[1]	Gene[1, 2]	Sigma factor[2]	Detected in this study?	Biological function[1]
Central carbon metabolism	*glk*	RpoS, RpoD	No	Upper glycolysis
	pgi	RpoS, RpoD	No	Upper glycolysis
	pfkA	RpoS, RpoD	Yes	Upper glycolysis
	pfkB	RpoS	Yes	Upper glycolysis
	glpX	RpoS	No	Upper glycolysis
	fbaA	RpoS, RpoD	Yes	Upper glycolysis
	fbaB	RpoS	Yes	Upper glycolysis
	tpiA	RpoS, RpoD	Yes	Upper glycolysis
	gapA	RpoS, RpoD	Yes	Lower glycolysis
	pgk	RpoS, RpoD	Yes	Lower glycolysis
	gpmA	RpoS, RpoD	Yes	Lower glycolysis
	gpmM	RpoS	No	Lower glycolysis
	eno	RpoS, RpoD	Yes	Lower glycolysis
	pykA	RpoS, RpoD	Yes	Lower glycolysis
	pykF	RpoS, RpoD	Yes	Lower glycolysis
	aceE	RpoS, RpoD	Yes	Pyruvate decarboxylation to acetyl-CoA
	aceF	RpoS, RpoD	Yes	Pyruvate decarboxylation to acetyl-CoA
	lpdA	RpoS, RpoD	Yes	Pyruvate decarboxylation to acetyl-CoA and TCA cycle
	dhaK	RpoS	Yes	Glycerol metabolism
	dhaL	RpoS	No	Glycerol metabolism
	dhaM	RpoS	No	Glycerol metabolism
	talA	RpoS	Yes	Pentose phosphate pathway
	tktB	RpoS	Yes	Pentose phosphate pathway
	acs	RpoS, RpoD	Yes	By-product metabolism
	adhE	RpoS, RpoD	Yes	By-product metabolism
	poxB	RpoS, RpoD	Yes	By-product metabolism
	acnA	RpoS, RpoD	Yes	TCA cycle
	fumC	RpoS	Yes	TCA cycle
	sucA	RpoS	Yes	TCA cycle
	sucB	RpoS	Yes	TCA cycle
	sucC	RpoS	Yes	TCA cycle
	sucD	RpoS	Yes	TCA cycle
Electron transport chain	*appB*	RpoS, RpoD	No	Aerobic electron transport chain; Oxidative phosphorylation
	appC	RpoS, RpoD	No	Aerobic electron transport chain; Oxidative phosphorylation
	frdA	RpoS, RpoD	No	Anaerobic respiration; Electron transport chain
	frdB	RpoS, RpoD	No	Anaerobic respiration; Electron transport chain
	frdC	RpoS, RpoD	No	Anaerobic respiration; Fumarate metabolic process
	frdD	RpoS, RpoD	No	Anaerobic respiration; Fumarate metabolic process
	ynfG	RpoS, RpoD	No	Electron transport chain
Biosynthetic process	*speC*	RpoS	No	Cellular amino acid metabolic process; Spermidine and putrescine biosynthetic process
	adrA	RpoS	No	Cyclic nucleotide biosynthetic process
	folK	RpoS, RpoD	No	Folic acid biosynthetic process; Tetrahydrofolate biosynthetic process
	fau	RpoS, RpoD	No	Folic acid-containing compound biosynthetic process
	glgC	RpoS	No	Glycogen biosynthetic process
	cfa	RpoS, RpoD	No	Lipid and fatty acid biosynthetic process
	lpxM	RpoS	No	Lipopolysaccharide biosynthetic process
	luxS	RpoS	Yes	Methionine biosynthetic process; Generation of a signal involved in cell-cell signaling
	panZ	RpoS	No	Pantothenate biosynthetic process

Table S4 - RpoS controlled genes of *E. coli*

Category[1]	Gene[1, 2]	Sigma factor[2]	Detected in this study?	Biological function[1]
Biosynthetic process	*yegS*	RpoS	No	Phospholipid biosynthetic process
	ppk	RpoS	No	Polyphosphate biosynthetic process
	speB	RpoS	No	Putrescine spermidine & putrescine biosynthetic process
Catabolic process	*astA*	RpoS	Yes	Arginine catabolic process
	astB	RpoS	Yes	Arginine catabolic process
	astC	RpoS	No	Arginine catabolic process
	astD	RpoS	Yes	Arginine catabolic process
	astE	RpoS	No	Arginine catabolic process
	gadA	RpoS, RpoD	No	Glutamate metabolic process
	gadB	RpoS, RpoD	No	Glutamate metabolic process
	ldcC	RpoS	No	Lysine catabolic process
	puuE	RpoS	No	Putrescine catabolic process; Gamma-aminobutyric acid metabolic process
	puuA	RpoS	No	Putrescine catabolic process; Glutamine biosynthetic process
	puuD	RpoS	No	Putrescine catabolic process; Glutamine biosynthetic process
	mtn	RpoS, RpoD	No	Nucleoside catabolic process; L-methionine biosynthetic process
	xapA	RpoS, RpoD	No	Purine nucleoside catabolic process
	hofM	RpoS	No	Utilization of DNA as a carbon source
	hofN	RpoS	No	Utilization of DNA as a carbon source
	hofO	RpoS	No	Utilization of DNA as a carbon source
	hofP	RpoS	No	Utilization of DNA as a carbon source
	murQ	RpoS, RpoD	No	Amino sugar catabolic process
	yibQ	RpoS	No	Carbohydrate metabolic process; Putative nucleoside (IDP) diphosphatase
	yadE	RpoS	No	Carbohydrate metabolic process
	galE	RpoS, RpoD	No	Galactose catabolic process; Colanic acid biosynthetic process
	galT	RpoS, RpoD	No	Galactose catabolic process; Colanic acid biosynthetic process
	galK	RpoS, RpoD	No	Galactose catabolic process
	galM	RpoS, RpoD	No	Galactose catabolic process
	gabT	RpoS, RpoD	Yes	Gamma-aminobutyric acid catabolic process
	glgP	RpoS	No	Glycogen catabolic process
	rssA	RpoS	No	Lipid & phosphatidylcholine metabolic process
	lsrF	RpoS	No	Metabolic proces
	ppx	RpoS	No	Polyphosphate catabolic proces
	treF	RpoS	No	Trehalose catabolic process
	dacB	RpoS	No	Peptidoglycan catabolic process; Cell wall organization
	sohB	RpoS, RpoD	No	Proteolysis
	yegQ	RpoS, RpoD	No	Proteolysis
Transport system	*actP*	RpoS, RpoD	No	Acetate & glycolate transmembrane transport
	araF	RpoS, RpoD	No	Arabinose ABC transporter
	araG	RpoS, RpoD	No	Arabinose ABC transporter
	araH	RpoS, RpoD	No	Arabinose ABC transporter
	murP	RpoS, RpoD	No	Carbohydrate transport (PTS system)
	sgrT	RpoS, RpoD	No	Carbohydrate transport (PTS system); Regulating the activity of PtsG
	mglA	RpoS, RpoD	No	Galactose ABC transporter
	mglB	RpoS, RpoD	No	Galactose ABC transporter
	mglC	RpoS, RpoD	No	Galactose ABC transporter
	setA	RpoS, RpoD	No	Glucose & lactose transport; Response to stress
	fadL	RpoS	Yes	Long-chain fatty acid and lipid transport

Table S4 - RpoS controlled genes of *E. coli*

Category[1]	Gene[1, 2]	Sigma factor[2]	Detected in this study?	Biological function[1]
Transport system	*gabP*	RpoS, RpoD	No	Amino acid transmembrane transport; Response to DNA damage stimulus
	ansP	RpoS	No	Amino acid transmembrane transport
	gadC	RpoS, RpoD	No	Amino acid transmembrane transport
	proP	RpoS	No	Amino acid transmembrane transport
	artM	RpoS	No	Arginine ABC transporter
	artP	RpoS	No	Arginine ABC transporter
	artQ	RpoS	No	Arginine ABC transporter
	artI	RpoS	No	Arginine ABC transporter
	proV	RpoS, RpoD	No	Proline ABC transporter; Response to osmotic stress
	proW	RpoS, RpoD	No	Proline ABC transporter; Response to osmotic stress
	proX	RpoS, RpoD	No	Proline ABC transporter; Response to osmotic stress
	yddG	RpoS	No	Phenylalanine tryptophan & tyrosine transport
	lsrA	RpoS	No	Autoinducer-2 ABC transporter
	lsrB	RpoS	No	Autoinducer-2 ABC transporter
	lsrC	RpoS	No	Autoinducer-2 ABC transporter
	lsrD	RpoS	No	Autoinducer-2 ABC transporter
	pstB	RpoS, RpoD	No	Phosphate ABC transporter
	pstC	RpoS, RpoD	No	Phosphate ABC transporter
	pstA	RpoS, RpoD	No	Phosphate ABC transporter; Response to DNA damage stimulus
	pstS	RpoS, RpoD	No	Phosphate ABC transporter; Response to DNA damage stimulus
	btuF	RpoS, RpoD	No	Vitamin B12 ABC transporter
	znuA	RpoS	No	Zn^{2+} ABC transporter
	mdtA	RpoS	No	Drug transmembrane transport
	mdtB	RpoS	No	Drug transmembrane transport
	mdtC	RpoS	No	Drug transmembrane transport
	mdtK	RpoS, RpoD	No	Drug transmembrane & sodium ion transport; Response to reactive oxygen species
	mdtE	RpoS	No	Multidrug transport system; Response to antibiotic
	mdtF	RpoS	No	Multidrug transport system; Response to antibiotic
	narU	RpoS	No	Nitrite transmembrane transport
	xapB	RpoS, RpoD	No	Nucleoside transport & xanthosine transport
	nhaA	RpoS	No	Sodium ion transmembrane transport
	yabI	RpoS	No	Conserved inner membrane protein
	yadS	RpoS, RpoD	No	Conserved inner membrane protein
	yjcH	RpoS, RpoD	No	Conserved inner membrane protein
	ygiB	RpoS	No	Conserved outer membrane protein
	pqiB	RpoS, RpoD	No	Integral to plasma membrane
	tolC	RpoS	Yes	Outer membrane porin
	yccB	RpoS, RpoD	No	Small outer membrane protein
	csgE	RpoS, RpoD	No	Protein transmembrane transport; Single-species biofilm formation
	csgG	RpoS, RpoD	No	Protein transmembrane transport; Single-species biofilm formation
	osmF	RpoS	No	Transport
	blc	RpoS	No	Transport; Response to DNA damage stimulus
	htrE	RpoS	No	Transport; Response to stress; Pilus organization
	fliY	RpoS	No	Transport (ABC superfamily)
	yehW	RpoS	No	Transport (ABC superfamily)
	yehX	RpoS	No	Transport (ABC superfamily)
	yehY	RpoS	No	Transport (ABC superfamily)
	yjjK	RpoS	Yes	Transport (ABC superfamily)
	ddpA	RpoS	No	Predicted peptide ABC transporter protein
	ddpB	RpoS	No	Predicted peptide ABC transporter protein

Table S4 - RpoS controlled genes of *E. coli*

Category[1]	Gene[1, 2]	Sigma factor[2]	Detected in this study?	Biological function[1]
Transport system	*ddpC*	RpoS	No	Predicted peptide ABC transporter protein
	ddpD	RpoS	No	Predicted peptide ABC transporter protein
	ddpF	RpoS	No	Predicted peptide ABC transporter protein
	ydhU	RpoS	No	Predicted cytochrome b-containing integral membrane protein
	ychE	RpoS	No	Predicted inner membrane protein
	yhhT	RpoS	No	Predicted inner membrane protein
	yigM	RpoS	No	Predicted inner membrane protein
	yhjG	RpoS	No	Predicted outer membrane biogenesis protein
	mdtD	RpoS	No	Predicted transmembrane transport
	yjcC	RpoS	No	Integral to membrane; Predicted c-di-GMP-specific phosphodiesterase
	yqiG	RpoS	No	Putative membrane protein
	yhiD	RpoS, RpoD	No	Putative ATP dependent transporter
Regulation of transcription	*glgS*	RpoS	No	Regulation of carbohydrate metabolic process
	fic	RpoS	No	Regulation of cell division
	mpl	RpoS, RpoD	Yes	Regulation of cell shape and division; Peptidoglycan biosynthetic process
	gadY	RpoS	No	Regulation of gene expression
	cyaR	RpoS, RpoD, RpoE	No	Regulation of mRNA stability
	rraA	RpoS	No	Regulation of RNA metabolic process; RNA catabolic process
	puuR	RpoS	No	Regulation of transcription; Putrescine catabolic process
	rhaR	RpoS, RpoD	No	Regulation of transcription; Rhamnose metabolic process
	rhaS	RpoS, RpoD	No	Regulation of transcription; Rhamnose metabolic process
	ilvY	RpoS, RpoD	No	Regulation of transcription; Amino acid biosynthetic process
	evgA	RpoS	No	Regulation of transcription; Phosphorelay signal transduction system
	evgS	RpoS	No	Regulation of transcription; Phosphorelay signal transduction system
	gadE	RpoS	No	Regulation of transcription; Phosphorelay signal transduction system
	rssB	RpoS	No	Regulation of transcription; Phosphorelay signal transduction system
	rpoH	RpoS, RpoD	No	Regulation of transcription; Response to stress
	bolA	RpoS, RpoD	No	Regulation of transcription
	csgD	RpoS, RpoD	No	Regulation of transcription
	csiE	RpoS, RpoD	No	Regulation of transcription
	dhaR	RpoS	No	Regulation of transcription
	gadW	RpoS	No	Regulation of transcription
	hdfR	RpoS	No	Regulation of transcription
	ihfA	RpoS	No	Regulation of transcription
	ihfB	RpoS	No	Regulation of transcription
	mlrA	RpoS, RpoD	No	Regulation of transcription
	murR	RpoS, RpoD	No	Regulation of transcription
	nhaR	RpoS	No	Regulation of transcription
	pdhR	RpoS, RpoD	No	Regulation of transcription
	rsd	RpoS	No	Regulation of transcription
	yeiL	RpoS	No	Regulation of transcription
	csrA	RpoS, RpoD	No	Regulation of translational initiation
	yhaJ	RpoS	No	Predicted transcriptional regulator
	yiaG	RpoS	No	Predicted transcriptional regulator
	gmr	RpoS	No	Posttranscriptional regulation of gene expression; Cyclic nucleotide biosynthetic process
Response to stress and damage	*exoX*	RpoS	No	DNA mismatch repair
	alkB	RpoS, RpoD	No	DNA repair; DNA demethylation
	gadX	RpoS, RpoD	No	Response to DNA damage stimulus; Regulation of transcription
	iraD	RpoS, RpoD	No	Response to DNA damage stimulus; Regulation of transcription
	yciT	RpoS	No	Response to DNA damage stimulus; Regulation of transcription

Table S4 - RpoS controlled genes of *E. coli*

Category[1]	Gene[1, 2]	Sigma factor[2]	Detected in this study?	Biological function[1]
Response to stress and damage	*treA*	RpoS	Yes	Response to DNA damage stimulus; Trehalose catabolic process
	glgA	RpoS	No	Response to DNA damage stimulus; Glycogen biosynthetic process
	yqiH	RpoS	No	Response to DNA damage stimulus; Cell wall organization
	dnaN	RpoS, RpoD	No	Response to DNA damage stimulus
	ada	RpoS, RpoD	No	Response to DNA damage stimulus & DNA repair; Regulation of transcription
	alkA	RpoS, RpoD	No	Response to DNA damage stimulus & DNA repair
	phr	RpoS	No	Response to DNA damage stimulus & DNA repair
	xthA	RpoS	No	Response to DNA damage stimulus & DNA repair
	yjiY	RpoS	No	Response to DNA damage stimulus & Starvation
	mutS	RpoS	No	Response to DNA damage stimulus and mismatch repair
	recF	RpoS, RpoD	No	SOS response & DNA synthesis involved in DNA repair
	yafN	RpoS, RpoD	No	SOS response & DNA repair; Regulation of transcription
	yafO	RpoS, RpoD	No	SOS response & DNA repair; Regulation of translation
	yafP	RpoS, RpoD	No	SOS response & DNA repair
	dinB	RpoS, RpoD	No	SOS response & DNA synthesis involved in DNA repair
	dkgB	RpoS	No	Oxidation-reduction process; Methylglyoxal catabolic process
	gabD	RpoS, RpoD	Yes	Oxidation-reduction process; Gamma-aminobutyric acid catabolic process
	gor	RpoS, RpoD	Yes	Oxidation-reduction process; Glutathione metabolic process
	puuB	RpoS	No	Oxidation-reduction process; Putrescine catabolic process
	puuC	RpoS	No	Oxidation-reduction process; Putrescine catabolic process
	epd	RpoS, RpoD	No	Oxidation-reduction process; Pyridoxal phosphate biosynthetic process
	hyaA	RpoS, RpoD	No	Oxidation-reduction process; Aerobic and anaerobic respiration
	hyaB	RpoS, RpoD	No	Oxidation-reduction process; Aerobic and anaerobic respiration
	hyaC	RpoS, RpoD	No	Oxidation-reduction process; Aerobic and anaerobic respiration
	ynfH	RpoS, RpoD	No	Oxidation-reduction process; Anaerobic electron transport chain
	ynfE	RpoS, RpoD	No	Oxidation-reduction process; Cellular respiration
	ynfF	RpoS, RpoD	No	Oxidation-reduction process; Cellular respiration
	lsrG	RpoS	No	Oxidation-reduction process; Quorum sensing
	katE	RpoS	No	Oxidation-reduction process; Response to DNA damage stimulus
	ahr	RpoS	No	Oxidation-reduction process
	lhgO	RpoS	No	Oxidation-reduction process
	osmC	RpoS	No	Oxidation-reduction process
	yciE	RpoS	No	Oxidation-reduction process
	ydhV	RpoS	No	Oxidation-reduction process
	oxyR	RpoS, RpoD	Yes	Oxidation-reduction process & response to DNA damage stimulus; Regulation of transcription
	aldB	RpoS	Yes	Oxidation-reduction process & response to DNA damage stimulus
	yciF	RpoS	No	Oxidation-reduction process & response to DNA damage stimulus
	hmp	RpoS	No	Oxidation-reduction process & response to nitrosative stress
	dps	RpoS, RpoD	Yes	Oxidation-reduction process & response to starvation; Chromosome condensation
	sodC	RpoS	No	Removal of superoxide radicals
	ariR	RpoS, RpoD	No	Response to hydrogen peroxide & acid; Biofilm formation
	aidB	RpoS, RpoD	No	Response to stress and oxidation-reduction process; Regulation of transcription
	yggE	RpoS	No	Response to reactive oxygen species
	appY	RpoS	No	Response to phosphate starvation; Regulation of transcription
	appA	RpoS, RpoD	No	Response to phosphate starvation and anoxia
	ddpX	RpoS	No	Response to starvation; Proteolysis; Peptidoglycan biosynthetic process
	phoU	RpoS, RpoD	No	Response to starvation, heat, acid, antibiotic; Regulation of gene expression
	sgrS	RpoS, RpoD	No	Response to stress; Regulation of transcription

Table S4 - RpoS controlled genes of *E. coli*

Category[1]	Gene[1, 2]	Sigma factor[2]	Detected in this study?	Biological function[1]
Response to stress and damage	*yadV*	RpoS	No	Response to stress; Cell wall and pilus organization
	pqiA	RpoS, RpoD	No	Response to stress
	uspB	RpoS	No	Response to stress & ethanol
	hchA	RpoS	No	Response to stress & acid; Methylglyoxal catabolic process; Lactate biosynthetic process
	ecnB	RpoS	No	Response to toxic substance
	yqiI	RpoS	No	Response to methylglyoxal; Cell adhesion
	ydbD	RpoS	No	Response to methylglyoxal
	ycgZ	RpoS, RpoD	No	Response to acid; Biofilm formation
	asr	RpoS, RpoD	No	Response to acid
	hdeA	RpoS, RpoD	No	Response to acid
	hdeB	RpoS, RpoD	No	Response to acid
	yhiM	RpoS	No	Response to acid
	osmB	RpoS, RpoD	No	Response to osmotic stress
	osmE	RpoS, RpoD	No	Response to osmotic stress
	osmY	RpoS, RpoD	No	Response to osmotic stress
	otsA	RpoS	Yes	Response to osmotic stress; Trehalose biosynthetic process
	otsB	RpoS	No	Response to osmotic stress; Trehalose biosynthetic process
Others	*ymgA*	RpoS, RpoD	No	Biofilm formation
	ymgC	RpoS, RpoD	No	Biofilm formation
	csgA	RpoS, RpoD	No	Single-species biofilm formation; Amyloid fibril formation
	csgB	RpoS, RpoD	No	Single-species biofilm formation; Amyloid fibril formation
	csgF	RpoS, RpoD	No	Single-species biofilm formation
	envC	RpoS	No	Cell cycle and division; Autolysis
	ftsA	RpoS, RpoD	No	Cell cycle and division
	ftsB	RpoS	No	Cell cycle and division; Cytokinesis by binary fission
	ftsQ	RpoS, RpoD	No	Cell cycle and division; Cytokinesis by binary fission
	ftsZ	RpoS, RpoD	Yes	Cell cycle and division; Cytokinesis by binary fission
	insC-5	RpoS	No	DNA recombination; Transposition
	insD-5	RpoS	No	DNA recombination; Transposition
	topA	RpoS, RpoD	No	DNA topological change; DNA strand elongation
	hrpB	RpoS	No	DNA-dependent DNA replication; pilus organization
	gyrB	RpoS, RpoD	Yes	DNA-dependent DNA replication
	insI-1	RpoS	No	Transposition; DNA integration
	insO-1	RpoS	No	Transposition; Putative transposase
	insCD-5	RpoS	No	Transposition; DNA recombination
	insN-1	RpoS	No	Transposition; DNA recombination
	sra	RpoS	No	30S ribosomal subunit protein S22
	ssrS	RpoS, RpoD	No	6S RNA
	tam	RpoS	No	Methylation
	pcnB	RpoS, RpoD	No	mRNA polyadenylation and transcription
	glyS	RpoS	Yes	Protein biosynthesis; Arginyl-tRNA & glycyl-tRNA aminoacylation
	cbpA	RpoS	Yes	Protein folding; DNA binding
	slyD	RpoS	Yes	Protein peptidyl-prolyl isomerization; Peptidyl-proline modification
	hyaD	RpoS, RpoD	No	Proteolysis & protein processing
	cbpM	RpoS	No	Regulation of catalytic activity; Enzyme inhibitor activity
	rhlE	RpoS	No	Ribosome assembly (RNA helicase)
	dmsD	RpoS, RpoD	No	Signal sequence binding; Protein binding
	arrS	RpoS, RpoD	No	Small RNA

Table S4 - RpoS controlled genes of *E. coli*

Category[1]	Gene[1, 2]	Sigma factor[2]	Detected in this study?	Biological function[1]
Others	*ydhX*	RpoS	No	Iron-sulfur cluster binding; Predicted electron transfer protein
	ydhY	RpoS	No	Electron carrier activity; Predicted ferredoxin-like protein
	yihG	RpoS	No	Metabolic process; Predicted endonuclease
	csgC	RpoS, RpoD	No	Putative curli production protein
	ybhB	RpoS	No	Not known; Predicted kinase inhibitor
	ybjP	RpoS	No	Not known; Predicted lipoprotein
	csiD	RpoS	No	Not known
	dsrB	RpoS	No	Not known
	hyaE	RpoS, RpoD	No	Not known
	hyaF	RpoS, RpoD	No	Not known
	msyB	RpoS	No	Not known
	ybgA	RpoS	No	Not known
	ybiI	RpoS	No	Not known
	yciG	RpoS	No	Not known
	yciZ	RpoS	No	Not known
	ydhT	RpoS	No	Not known
	ydhW	RpoS	No	Not known
	ydjO	RpoS, RpoH	No	Not known
	yffO	RpoS	No	Not known
	yffP	RpoS	No	Not known
	ygaU	RpoS	Yes	Not known
	ygiA	RpoS	No	Not known
	ygiC	RpoS	No	Not known
	yobB	RpoS	No	Not known
	ytfK	RpoS	No	Not known

[1] The information, nomenclature and classification are according to EcoCyc database [1] (http://ecocyc.org/).

[2] The information was extracted from RegulonDB Version 7.0 (http://regulondb.ccg.unam.mx) [2]. (The database will be updated on a regular basis)

[1] Keseler, I.M., et al., EcoCyc: a comprehensive database of *Escherichia coli* biology. Nucleic Acids Res, 2011. 39(Database issue): p. D583-90.

[2] Gama-Castro S, et al., RegulonDB version 7.0: transcriptional regulation of *Escherichia coli* K-12 integrated within genetic sensory response units (Gensor Units). *Nucleic Acids Res* 2011, 39:D98-105.
